# Integrating cardiovascular healthcare screening into a community pharmacy vaccination service: a scoping review to identify opportunities for patient engagement and service expansion

**DOI:** 10.1136/bmjopen-2025-108381

**Published:** 2026-03-23

**Authors:** Jason Tang, Gemma Mansell, Piotr Merks, Alison Jones, Jay Badenhorst, Mark Koziol, Daljit Sandhu, Chris Langley

**Affiliations:** 1 College of Health and Life Sciences, Aston University, Birmingham, UK; 2 The Pharmacists’ Defence Association, Birmingham, UK; 3 Department of Pharmacology and Clinical Pharmacology, Faculty of Medicine, Collegium Medicum, Cardinal Wyszyński University in Warsaw, Warsaw, Poland; 4 Priory Community Pharmacy, Dudley, UK

**Keywords:** Blood Pressure, Cardiovascular Disease, Health, Health Services, Quality in health care, Patient-Centered Care

## Abstract

**Abstract:**

**Objectives:**

Community pharmacies are increasingly recognised as accessible providers of public health services. Integrating cardiovascular health checks and behaviour change strategies with the community pharmacy vaccination service has the potential to improve population health outcomes. This scoping review aims to identify potential pharmacist-led cardiovascular-related services suitable for such integration, and to explore the acceptability and feasibility of pharmacy-led services among both service users and providers.

**Data sources:**

MEDLINE, EMBASE, CINAHL and Web of Science were systematically searched for UK-based empirical studies published between January 2013 and December 2024.

**Eligibility criteria:**

Primary studies reporting on pharmacist-led cardiovascular-related interventions (eg, blood pressure monitoring, cholesterol screening, smoking cessation) delivered to adults (≥18 years) in community pharmacy settings and reporting on clinical outcomes, feasibility or acceptability were included.

**Data extraction and synthesis:**

Data were extracted using a standardised form on Excel and synthesised narratively. Key domains of interest included intervention characteristics, facilitators, barriers, perceived outcomes, delivery mode and intervention content including behavioural change strategies or components to support implementation.

**Results:**

Of 8322 records screened, 53 studies met the inclusion criteria. Cardiovascular-related interventions were broadly feasible and acceptable to both patients and pharmacy staff. High patient satisfaction was attributed to the accessibility and convenience of pharmacy locations, as well as the ability of pharmacy staff to establish rapport during interactions. Facilitators of service delivery included private consultation spaces, structured training and access to digital screening tools (eg, devices for atrial fibrillation detection). Barriers included workload constraints and limited public awareness of pharmacy services. Five studies described successful integration of lifestyle interventions within pharmacy-based settings, but the long-term clinical outcomes produced by the intervention were rarely reported. Patients valued the convenience and trusted relationships with pharmacists, though concerns about privacy were raised. Pharmacists reported the need for clearer clinical protocols, and multidisciplinary support and training to improve their confidence in delivering health checks as part of their routine work.

**Conclusions:**

Community pharmacies offer an optimal setting for integrating cardiovascular-related screening interventions with the vaccination service delivered within community pharmacy. Successful implementation will require attention to the identified facilitators including the quality of staff training, competing priorities and optimisation and utilisation of pharmacy space. Future research should prioritise definitive controlled trials and cost-effectiveness analyses to assess long-term health outcomes. Policy action is also needed to support service integration and expand pharmacists’ public health roles within the wider National Health Service.

**Strengths and limitations of this study**

- This review provides a comprehensive synthesis of UK-based pharmacist-led cardiovascular-related services delivered within community pharmacy.
- Heterogeneity in study designs, intervention content and outcome measures limited comparability across studies.
- A scarcity of studies including long-term outcome data limited conclusions to be drawn on sustained effectiveness over time.

## Background

 In the UK, health inequalities are widening across socioeconomic groups and geographic regions.^1 2^ General practice (GP) surgeries and emergency departments are operating at full capacity, overwhelmed by mounting pressures on the National Health Service (NHS). These challenges stem from workforce shortages and the lingering impact of the COVID-19 pandemic.^3^ Influenza vaccines have been available from community pharmacies since September 2015 and since the COVID-19 pandemic, community pharmacies have also administered COVID-19 vaccinations. The COVID-19 pandemic not only highlighted the potential of community pharmacies in delivering vaccinations but also prompted the legal and structural expansion of pharmacists’ roles internationally. Merks *et al* documented these changes, showing how legislative reforms enabled pharmacists to assume broader public health responsibilities, laying the foundation for the integration of wider preventative services.^4^

This pharmacy-led vaccination service was highly successful, with community pharmacies contributing to nearly half of the national vaccination effort in 2021 alone.^5^ Despite their proven success and potential for service expansion, there is currently no clear strategy within the NHS to continue pharmacy-led services.

Community pharmacies provide an optimal setting for delivering accessible and convenient healthcare services to the public. In addition to dispensing medications, they already provide a range of NHS-commissioned services, such as Pharmacy First, which enables pharmacists to manage common conditions like sore throat, shingles and impetigo.^6^ Healthy Living Pharmacies (HLPs) also play a key role in health promotion through initiatives like Making Every Contact Count, whereby patient-centred conversations are used to encourage lifestyle changes and facilitate referrals to relevant health services.^7^ Improving public uptake of these existing services and interventions could significantly improve prevention efforts and population health outcomes.

Community pharmacies in the UK are now established as key providers of vaccination services.^5^ Integrating this pharmacy-led routine service with a broader range of brief health checks could further increase public uptake of these important preventative interventions. This is because the vaccination process itself provides an opportunity to engage patients in discussions about additional health concerns, offering advice or referrals to local services where needed. Such an approach would enable early identification of potential health risks that might otherwise go unnoticed due to competing priorities within the healthcare system. Early detection of disease risk would not only benefit individuals but could also reduce unnecessary GP visits, emergency department attendances and hospital admissions, thereby optimising the use of NHS resources.

An integrated health check and vaccination service, particularly one focused on cardiovascular health, could have a substantial impact on population health. This is because cardiovascular disease (CVD) remains a major public health concern in the UK, accounting for 28% of all deaths and costing the economy an estimated £19 billion annually.^8^,^10^ According to the British Heart Foundation, approximately 7.6 million people in the UK are living with CVD.^11^ Identifying at-risk individuals early—or those already living with CVD—is a critical public health priority, ensuring that limited resources, including referrals to appropriate treatments, are directed to those who need them the most.

Taking advantage of the community pharmacy setting to offer opportunistic cardiovascular-related health checks during vaccination appointments has the potential to boost uptake of these health check services. However, there is limited guidance on which combination of evidence-based health checks should be included in such an integrated service and whether they can be feasibly delivered in a community pharmacy context. Given the gaps in reporting within community pharmacy research, the pressing need to address this public health issue, and the lack of reviews on evidence-based community pharmacy cardiovascular interventions in the UK, a scoping review was warranted. As such, this review aimed to identify and summarise cardiovascular interventions that could be effectively integrated into community pharmacy-led vaccination services.

This scoping review examines cardiovascular-related interventions across community pharmacy settings to identify transferable approaches, components and delivery features that may support integration into vaccination services, rather than restricting inclusion to studies that have already been integrated within vaccination services.

The review findings aim to guide the development of a more comprehensive and coherent pharmacist-led integrated health check and vaccination service, designed for pragmatic implementation within community pharmacy settings. Additionally, these findings could inform future service enhancements and policy recommendations aimed at expanding the scope of pharmacist-led healthcare interventions in the community.

## Methods

### Study design

This scoping review was conducted in accordance with the Preferred Reporting Items for Systematic Reviews and Meta-Analyses Extension for Scoping Reviews guidelines.^12^ The framework proposed by Arksey and O’Malley was adopted to guide the review process, which consisted of five stages: (1) identifying the research question, (2) identifying relevant studies, (3) selecting studies, (4) charting the data and (5) collating, summarising and reporting the results.^13^

### Stage 1: identifying the research question

The primary research question was: ‘What cardiovascular interventions can be delivered as part of community pharmacist-led vaccination services?’. To address this overarching research question, the review explored the following key questions:

1. What are service users’ experiences of community pharmacist-led cardiovascular-related interventions?
2. What are pharmacists’ experiences of delivering such interventions?
3. Can the effectiveness of these interventions be enhanced through the use of supporting resources? (By supporting resources, we mean any physical or environmental support that can enhance intervention delivery)
4. Is effectiveness influenced by specific delivery modes or formats?

### Stage 2: identifying relevant studies

A comprehensive search strategy was developed using the Population, Concept and Context framework. Free-text search terms were constructed based on three main categories: (1) population (adults aged 18 years or older), (2) concept (cardiovascular-related health interventions) and (3) context (community pharmacy settings).

The search was performed across four electronic databases: CINAHL, MEDLINE, EMBASE and Web of Science, in October 2023, and was last updated in December 2024. The search was limited to publications between November 2013 and December 2024 as during this period, community pharmacies across the UK focused more on health screening and preventative services as opposed to dispensing alone. To ensure comprehensiveness, backward citation searches were conducted using reference lists of the included studies, and forward citation checks were made using Google Scholar. Our search strategy was developed in consultation with a librarian who has expertise in designing search strategies for systematic and scoping reviews. The librarian also assisted in executing the searches across the selected databases to ensure the identification of relevant studies. A detailed search strategy used for all electronic databases is provided in online supplemental file 1.

### Stage 3: selecting studies

Studies were eligible for inclusion if they met the following criteria:

#### Inclusion

1. Published in English.
2. Focused on UK-based pharmacist-led interventions targeting cardiovascular risk factors.
3. Included brief interactive sessions (some form of interaction between pharmacy staff and service users (eg, consultation or discussion via telephone)).
4. Involved adult populations (aged 18 years or older).
5. Reported quantitative or qualitative data on at least one of the following cardiovascular risk factors: body weight, body mass index, waist circumference, blood pressure (BP), cholesterol levels, glucose levels or triglycerides.

#### Exclusion

Studies focused exclusively on non-pharmacy-based interventions or did not report outcomes relevant to cardiovascular health. Additionally, reviews and grey literature including dissertations, conference presentations and reports were excluded. The selection process involved two independent reviewers. The first reviewer (JT) screened titles and abstracts to identify potentially eligible studies. Full-text reviews were then conducted by both reviewers (JT and GM) to confirm eligibility. Any discrepancies were resolved through discussion.

### Stage 4: charting the data

Data extraction was performed using a standardised template developed in Microsoft Excel. Extracted information included study aims, population characteristics, study design, intervention details, setting and outcome measures. The second reviewer (GM) independently verified the accuracy of all extracted data to ensure reliability. Due to the heterogeneity of the included studies in terms of methodology, settings and intervention designs, formal quality assessment was not conducted, which is consistent with similar scoping reviews in healthcare.

### Stage 5: collating, summarising and reporting results

The findings were synthesised narratively to address the research questions. Quantitative and qualitative data were analysed separately to provide a comprehensive overview of the evidence. Key themes related to patient and pharmacist experiences, as well as the effectiveness of supporting resources and delivery formats, were identified, presented at project steering group meetings and discussed with the project team.

### Patient and public involvement

While no patients or members of the public were directly involved in this review, the study formed part of a broader research programme supported by a research advisory group. This group included two patient and public representatives who provided input during the conceptualisation phase of community pharmacy services that could benefit them, ensuring that the review’s aims and purpose aligned with the needs of our key stakeholders.

## Results

**Figure 1:**
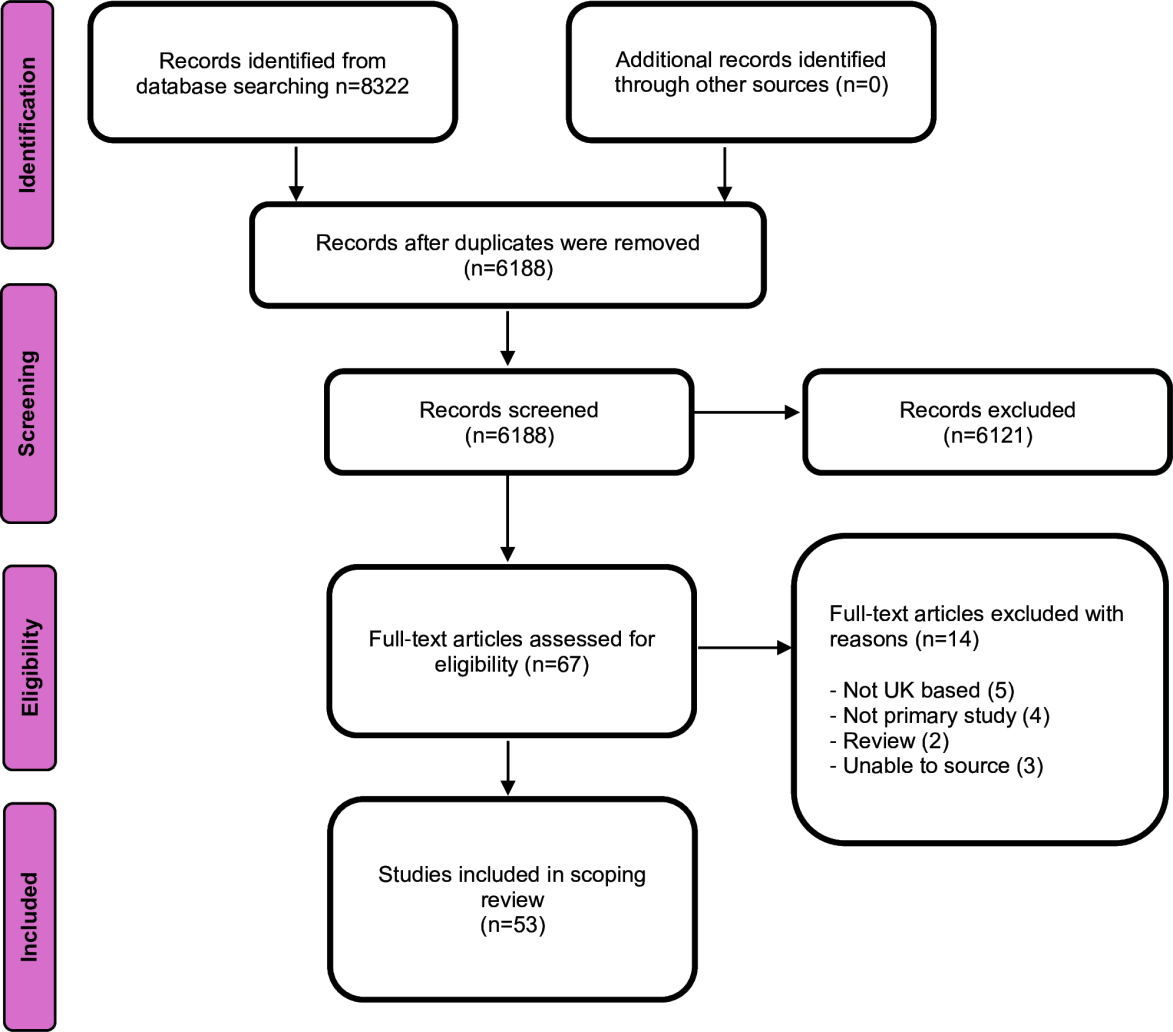
Study selection; PRISMA flow diagram. PRISMA, Preferred Reporting Items for Systematic Reviews and Meta-Analyses.

Our initial database searches identified 8322 records. After removing duplicates and screening of titles and abstracts, 67 articles remained for full-text assessment. Of these, 53 met the eligibility criteria and were included in the final review. Figure 1 illustrates the study selection process, from initial identification to final inclusion. A complete list of excluded full-text articles, along with reasons for exclusion, is available in online supplemental file 2.

### General characteristics of included studies

A summary of the characteristics of all 53 of the included UK-based studies is provided in online supplemental table 1. Studies predominantly included older adults (aged 65 years or older). Most studies targeted populations with cardiovascular-related health conditions, though some included adult participants without specifying whether they had a particular health condition. Of the 53 empirical studies, a range of methods were used, with 14 studies employing mixed methods,^14^,^27^ 10 employing qualitative methods (8 used individual interviews^28^,^35^ and 2 used focus groups).^36 37^ The remaining 29 used quantitative methods (8 used cross-sectional surveys,^38^,^45^ 6 used randomised controlled trials (RCTs),^846^,^50^ 4 used feasibility or pilot studies,^51^,^54^ 1 used longitudinal design,^55^ 2 were cohort studies,^56 57^ 3 used retrospective analysis,^58^,^60^ 2 were economic evaluations^61 62^ and 2 were non-randomised studies).^63 64^

### What cardiovascular interventions can be delivered as part of pharmacist-led vaccination services?

Our scoping review identified a range of evidence-based cardiovascular-related health checks that could be integrated into vaccination services delivered within community pharmacies. These included BP monitoring,^8 25 32 39 44 60^ cholesterol screening,^25 32 33^ diabetes risk assessments,^25 61 62^ lifestyle advice on smoking cessation,^15 16 51^ weight management^14 16^ and alcohol consumption.^14 16 43 50^

Five studies demonstrated the feasibility and acceptability of delivering brief services within UK community pharmacies, with overall promising results on patient engagement and outcomes.^3252^,^54 65^ These studies demonstrate the potential of delivering targeted public health services within community pharmacy settings.

In an observational pilot study, Jumbe *et al* evaluated the fidelity of the STOP (Smoking Treatment Optimisation in Pharmacies) intervention for smoking cessation, which was delivered by pharmacy staff across five community pharmacy sites.^54^ The STOP training intervention, which included behaviour change techniques, a patient-centred approach and provision of education on the health consequences of smoking, was assessed using six simulated smokers. Findings demonstrated that pharmacists who had completed STOP training demonstrated improvements in their consultation style and increased their use of intervention-related materials. Further, staff reported increased knowledge and self-efficacy in providing smoking cessation advice. Although the service duration was unclear, the findings highlight the importance of structured training in enhancing pharmacists’ readiness to deliver such behavioural interventions. The authors advocated for further testing in community pharmacy settings to support their findings as well as to inform wider implementation strategies.

Poole *et al* conducted a qualitative study to explore men’s experiences of a 12-week, multicomponent lifestyle intervention delivered by nine community pharmacies.^32^ The intervention, designed for men with prostate cancer, included health assessments, fitness evaluations, lifestyle feedback and telephone-based support. Framework analysis identified two key themes (1) the creation of teachable moments and (2) the social process of making lifestyle changes. Participants valued the accessibility and informality of the community pharmacy setting, though some reported that the advice was less tailored than that offered in primary care. Further, two participants reported smoking cessation as a direct result of the intervention, suggesting that community pharmacies could be effective in supporting lifestyle change in men with prostate cancer.

In a prospective cohort study, Wright *et al* evaluated the feasibility of a pharmacy-based chronic obstructive pulmonary disease (COPD) support service, which included smoking cessation, medication adherence support and general lifestyle guidance.^65^ Data were collected from 66 community pharmacies providing free BP checks. Results showed that the intervention was associated with significant improvements in quality of life and reductions in COPD assessment test scores, as well as increased uptake of rescue packs and reported reductions in smoking. Although the service duration was unclear, these findings support the role of community pharmacists in managing chronic respiratory conditions through an integrated, person-centred approach.

Jalal *et al* conducted a pilot-controlled trial examining the impact of a pharmacist-led motivational interviewing intervention on adherence to secondary prevention medications among patients recently discharged from hospital following a coronary event.^53^ The intervention, delivered in person or via telephone within the New Medicine Service (NMS) and Medicines Use Review (MUR) frameworks, consisted of a 15–20 min consultation with patients Statistically significant improvements in medication adherence were observed in the intervention group at 3 months (mean=7.7, SD=0.56) compared with controls (mean=7.0, SD=1.85; p=0.026), and this effect was maintained at 6 months. Although no significant differences were found in systolic BP or low density lipoprotein (LDL) cholesterol (which the authors stated may be due to missing data), the intervention was considered both feasible and acceptable. In turn, the authors recommended further testing of the service in a larger-scale trial to evaluate its effectiveness.

In a mixed-methods feasibility study, Holland-Hart *et al* investigated the implementation of a pharmacist-led referral service for urgent chest X-rays in socioeconomically deprived communities.^52^ Data were obtained through interviews with patients, pharmacists and general practitioners, as well as focus groups involving healthcare professionals and members of the public. The key findings showed that the pharmacy service was viewed as acceptable and potentially feasible with familiarity and ease of access highlighted as key strengths. However, limited public awareness of the service and concerns regarding pharmacists’ expertise were also found to be barriers to service uptake. Low referral rates highlighted the need for enhanced training, better public promotion and improved integration with secondary care. The authors concluded that a standardised approach to training and service delivery is necessary before considering wider implementation and further testing in an RCT.

Not all findings, however, from this group of studies, addressing this research question, were positive. Cheema *et al* conducted a small-scale RCT assessing whether structured verbal and written information on hypertension and antihypertensive medications would improve BP control in patients starting BP medication.^8^ While the intervention group achieved significantly greater reductions in systolic BP at 4 weeks, these effects were not sustained at 26 weeks, with both groups experiencing similar clinical outcomes towards the end of the study. These results highlighted that provision of information interventions may only result in short-term benefits and need further intervention for sustained long-term outcomes.

Chua *et al* discrete choice experiment study suggested that there was growing support for pharmacist-led cardiovascular screening services, including lipid profiling and diabetes risk assessments.^41^ Specifically, community pharmacies were valued for their accessibility, convenience and how they are embedded within local communities, particularly in underserved areas. However, Holland-Hart *et al* pointed out that the effectiveness and acceptability of such services depended on staff availability, the physical environment (eg, adequately sized consultation rooms where sensitive discussions can take place privately), and public awareness of service availability.^52^

#### Reported referral pathways

Some studies reported onward referral as a component of their intervention,^16 19 38 52 58 59 62^ though the level of detail reported varied across studies. Referral routes included referral from pharmacist to GP during medicines support,^38^ follow-up with the GP,^62^ rapid referral for diagnostic investigation^52^ and referral into behavioural or support services.^16 19 58 59^ Albasri *et al* reported that 5895 (4.5%) of patients were referred to GP within 2 weeks of starting an antihypertensive,^38^ while Corlett and Krska reported that 25 (23%) patients accepted a weight-management referral and 14 (22%) patients attended follow-up. Boardman and Avery also included scheduled follow-ups but reported substantial attrition by 3 months.^58^ Collectively, these findings indicate that referral and follow-up are feasible within pharmacy-led delivery, but are inconsistently reported across studies.

### What are service users’ experiences of community pharmacist-led services? (18 primary studies)

18 studies were identified that explored service users’ experiences of community pharmacist-led interventions, including qualitative interviews, surveys, focus groups and mixed-methods evaluations^1516 19 21 22 24 25 27 28 30^,^33 36 43^ (see online supplemental table 2). These studies involved a range of interventions including smoking cessation, alcohol brief intervention (BI), weight management and chronic disease support. Key themes identified across the studies included: Convenience, accessibility and familiarity; trust and rapport with pharmacists; barriers to engagement; personalisation of services; and behavioural change.

#### Convenience, accessibility and familiarity

Service users generally expressed high satisfaction with pharmacist-led services, mainly because of their convenience, accessibility and the pharmacist’s non-judgmental approach to conversation about health. For example, Krska and Mackridge found that 80% of surveyed participants felt comfortable discussing their alcohol use with pharmacists, indicating also that familiarity and trust were essential to the uptake of such services.^19^ The pharmacy’s location and extended working hours were also reported as strengths, particularly within underserved areas.^52^ Similarly, Saramunee *et al* also highlighted that proximity to the patient’s home or workplace, extended opening hours (specifically Saturday opening hours), and continuity of care, whereby patients interact with the same pharmacist over time, were highly valued by pharmacy service users.^44^ However, not all findings were positive, as some also commented that time constraints of pharmacy staff and lack of privacy during consultations occasionally undermined their experience.^19^

#### Trust and rapport with pharmacists

Trust and rapport between service users and pharmacists was also highlighted as an important facilitator of service use. For example, Mackridge *et al* highlighted the importance of pharmacists’ perceived credibility and approachability, and Saramunee *et al* reported that 89.6% of surveyed service users trusted pharmacists to maintain their confidentiality.^21 44^ However, some service users reported that they were uncomfortable when discussing sensitive topics (eg, alcohol or weight) unless pharmacists adopted a collaborative, empathetic tone.^30^ In the mixed-methods study by Krska *et al*, patient participants also valued the confidentiality and professionalism of pharmacists during alcohol screening and BIs.^19^ Some preferred though consulting with their GP for more complex health issues, because of their greater perceived authority and expertise.^28^

#### Barriers to engagement

Despite generally positive views of pharmacy services, there were some barriers to engagement that need to be addressed. Privacy concerns still emerged as a key issue, with some participants expressing discomfort in discussing sensitive topics, such as alcohol use and mental health, in open pharmacy environments.^21 52^

#### Personalisation of services

Service users expressed the importance of tailored and relevant advice to improve engagement. Specifically, Poole *et al* found that prostate cancer survivors appreciated interventions that linked lifestyle advice to their medical conditions (eg, prostate cancer),^32^ while Savickas (2020) found that personalised atrial fibrillation (AF) screening using digital tools (eg, smartphone-enabled ECGs) enhanced participation.^45^

#### Behavioural change

While behavioural changes were modest, interventions like alcohol screening and smoking cessation prompted reflection and incremental improvements to behaviour.^14 22^ For example, 65.58% of participants in Sturrock *et al* intended to change oral hygiene habits post-intervention.^27^ However, Dhital *et al* found that without follow-up resources, motivated patients struggled with sustained change.^36^ Perceptions of pharmacists’ roles also varied, with some perceiving them as holistic health advisors,^25^ while others viewed them as mainly dispensers of medicines.^24^

### What are pharmacists’ experiences of delivering such services? (Nine primary studies)

Nine studies were identified that explored pharmacists’ experiences of delivering public health and clinical services in community pharmacies^1415 19^,^22 27 28 52^ (see online supplemental table 3). These studies explored pharmacists’ perspectives in relation to the delivery of a range of services including smoking cessation, alcohol BI, chronic disease management and preventative health screening. While many pharmacists expressed enthusiasm for expanding their professional role, challenges such as workload pressures, training gaps and time constraints prevented them from undertaking additional responsibilities. Key themes identified included workload pressures and time constraints, environmental and physical barriers, and training and resource requirements.

#### Workload pressures and time constraints

Findings showed that the workload pressure faced by pharmacists often affected the quality of intervention delivery and reportedly discouraged the adoption of new services into routine work. Atkin *et al* and Brown *et al* reported that pharmacists found it challenging to holistically address patients’ health needs due to existing competing priorities such as dispensing, walk-in consultations and administrative tasks.^15 28^ Their findings suggested that interventions like MURs were formulaic and driven primarily by contractual obligations rather than focusing on patient-centred care. Similarly, the qualitative findings by Krska and Mackridge and Mackridge *et al* also suggested that staffing shortages and the community pharmacies’ fast-paced, busy environment often limited proactive engagement with patients, which is particularly important for services that require open discussion about sensitive topics such as alcohol misuse.^19 21^ In contrast, Lemanska *et al* study findings demonstrated that the delivery of a structured lifestyle intervention, whereby community pharmacists supported patients in making a behavioural change to their lifestyle to improve health, was both feasible and acceptable, and so success of a service could well depend on well-designed programmes with clear protocols.^20^

#### Environmental and physical barriers

The physical environment of community pharmacies also had an impact on the delivery of routine services. In Holland-Hart *et al*, for instance, pharmacists pointed out that the open pharmacy layouts discouraged patients from engaging in open and sensitive discussions, with 66% of participants expressing privacy concerns in such open spaces.^52^ Similarly, Mackridge *et al* also highlighted the importance of private consultation spaces to perform alcohol screening,^21^ while Brown *et al* highlighted the importance of strong leadership, support from the local healthcare commissioning organisation, and establishing good rapport with patients as key factors in enabling successful implementation of HLPs.^14^

#### Training and resource requirements

Pharmacists’ confidence in delivering interventions depended on the availability and quality of training. Brown *et al* and Lemanska *et al* highlighted that targeted training can improve pharmacists’ communication skills and job satisfaction, enabling them to address health behaviours like smoking and physical activity well in their consultations.^14 20^ However, Atkin *et al* and Krska and Mackridge highlighted that some pharmacists were uncomfortable when engaging in discussions around lifestyle behaviours (eg, alcohol use), attributing this to insufficient staff training as the primary issue, and indicating the importance of ongoing professional development that is relevant to their role and activities.^19 28^ In a similar vein, Sturrock *et al* demonstrated that quality training improved pharmacists’ knowledge of oral health which in turn had a positive impact on patient outcomes.^27^

### Can the effectiveness of these services be enhanced through the use of supporting resources? (Seven primary studies)

None of the studies directly assessed whether supporting resources could enhance service effectiveness using an RCT design. Lemanska *et al*, however, evaluated the acceptability and feasibility of a community pharmacy lifestyle intervention for men living with prostate cancer.^20^ The intervention included lifestyle change resources such as exercise DVDs (video recordings), pedometers, resistance exercise bands and provision of information including lifestyle advice and goal setting. Findings demonstrated that these supporting tools increased adherence to recommended healthy lifestyle behaviours (ie, diet and physical activity). Particularly, an increase of 34 min (95% CI 6 to 62) of moderate to vigorous physical activity at 3 months (on average). These changes, however, were not sustained beyond 6 months. Nonetheless, these findings are promising, and the authors advocated for a definitive trial to evaluate the effectiveness of their refined intervention more widely.

Seven other studies were identified that examined how supporting resources such as educational materials, decision aids and digital tools improved the quality of community pharmacy interventions.^17^,^1935 39 41 42^ For example, Dhital demonstrated that structured training for alcohol BIs resulted in marked improvements in pharmacists’ knowledge and skills (median 6-point increase in alcohol-related knowledge scores after training) and improved patient engagement during service delivery.^17^ While Barrett and Hodgkinson found that more reliable screenings could be achieved within pharmacies by using validated BP monitors and standardised protocols.^39^

Pharmacists equipped with tools like Alcohol Use Disorders Identification Test for Consumption (AUDIT-C), a brief alcohol screening tool, and who had the space/rooms to conduct consultations privately were important in initiating open alcohol-related discussions.^19^ Douglas *et al* also reported that pharmacists who did not receive nutrition training felt unprepared to confidently deliver services well.^18^

Privacy assurances also encouraged patients to engage with alcohol interventions, but the study did not directly link whether this contributed to reported long-term behavioural lifestyle changes.^19^ Barrett and Hodgkinson’s cross-sectional survey highlighted that the visibility of BP screening services within community pharmacies as well as ease of access in terms of its location led to increased public uptake and engagement.^39^ The study also highlighted significant barriers, including equipment calibration issues, lack of public awareness of the service and standardised training which could impact on effectiveness of service delivery and implementation.

### Is effectiveness influenced by specific delivery modes or formats? (Three primary studies)

Three studies also examined how delivery modes or formats (eg, in-person consultations, digital tools, group sessions) could have an impact on the success of community pharmacy-led interventions^45 50 61^ (see online supplemental table 1). Elliott *et al* evaluated the NMS, delivered via telephone and in-person with follow-up telephone calls, and found that this was highly effective in improving patient outcomes and reducing costs.^61^ Specifically, the NMS produced a mean increase of 0.04 quality-adjusted life-years (QALYs) per patient and reduced costs by £139 per patient compared with usual care. The incremental cost-effectiveness ratio of −£3166 indicated that the NMS were more cost effective than standard practice, with a 96% probability of being cost-effective at a £20 000/QALY threshold. Findings from this evaluation suggested that structured, personalised consultations using either telephone or in-person delivery mode or both can potentially improve medication adherence as well as economic outcomes.

In contrast, Savickas *et al* examined the use of digital technology, specifically the Kardia Mobile Device (KMD), for AF screening in community pharmacies.^45^ The study found that pharmacist-led AF screening using KMD was more accurate than pulse palpation, with 24 out of 26 AF cases correctly identified. Participants rated their experience highly, and 99% expressed willingness to participate in annual screenings. The digital format not only improved detection rates but also facilitated cost-effective integration into routine practice. Further, the study highlighted that relying solely on traditional methods (eg, pulse palpation) led to higher false-positive rates (7.8% vs 2.6% for KMD) in diagnoses. Although not directly evaluating the delivery mode per se, Stewart *et al*’s pilot trial evaluating the feasibility of the Medicines and Alcohol Consultation (MAC) intervention, which involved integration of alcohol discussions into medicine reviews, indicated a reduction in alcohol consumption (−7.23 units, 95% CI −17.87 to 2.99) at 2 months, but these findings were non-significant.^50^ They did, however, demonstrate the feasibility of implementing the MAC in community pharmacy with high consent (94%) and follow-up rates (92%). The findings indicated that in-person mode of delivery could be successful during the initial stages of engagement, but improvements and sustained changes may depend on use of additional components (eg, notifications, follow-up calls).

## Discussion

This scoping review synthesised evidence from 53 UK-based empirical studies that examined community pharmacist-led cardiovascular screening interventions, with a focus on both pharmacists’ and service users’ experiences, as well as the contextual factors influencing service effectiveness. Overall, the review found that pharmacist-led interventions were generally well-received by both providers and users, particularly those focused on BP monitoring, cholesterol screening, diabetes risk assessments, lifestyle advice on smoking cessation, weight management and alcohol consumption. Similarly, Merks *et al* evaluated a pharmacist-led intervention in Poland aimed at improving adherence among AF patients initiating dabigatran. The study demonstrated significantly improved patient understanding and medication adherence compared with standard pharmacy practice, highlighting the role of pharmacists in early-phase chronic cardiovascular treatment.^66^

Key facilitators identified across the studies included accessibility of community pharmacies, the patient and pharmacist relationship, and the use of supporting resources such as validated digital tools, tailored educational materials and protocols. Conversely, workload pressures, limited privacy, insufficient training and lack of resources were found to be barriers to effective implementation. Seven studies highlighted the potential of supporting resources to improve service quality, though no studies formally assessed this using large RCT designs. Five studies did, however, demonstrate the feasibility of integrating lifestyle and screening services with existing pharmacy services, highlighting its potential for widespread implementation.

Some of the identified studies also demonstrated the feasibility and positive impact of integrating additional clinical healthcare interventions within community pharmacy settings. For instance, Jumbe *et al* found that training programmes such as STOP enhanced pharmacy staff’s knowledge and self-efficacy in delivering smoking cessation advice, leading to improved patient engagement.^54^ Similarly, Poole *et al* showed that multicomponent lifestyle interventions targeting cancer survivors prompted participants to consider lifestyle changes, with some even reported to have stopped smoking.^32^ In line with these findings, Wright *et al* showed significant improvements in quality of life and reductions in COPD assessment test scores through a community pharmacy-based support service.^65^

The included studies were diverse in their methods employed and intervention type. There was also considerable variability in terms of sample sizes, intervention duration and outcome measures, which limited comparisons across studies. Further, many studies did not include long-term follow-up or validated behavioural outcome measures, meaning that it was not possible to conclude whether the short-term improvements resulting from service use were sustained over time. There is a need for larger-scale, RCTs with embedded process evaluation studies to assess the sustained impact and cost-effectiveness of community pharmacist-led cardiovascular-related screening services.

Despite the promising findings, there are a few challenges that need to be borne in mind. Cheema *et al* highlighted that while structured information on hypertension led to short-term improvements in systolic BP, this effect was not maintained at 26 weeks.^8^ This suggests that ongoing support mechanisms or continuity of care need to be considered in conjunction with service use in order to maintain long-term benefits. Privacy concerns also emerged as a recurring theme, particularly for interventions involving sensitive topics like alcohol use or weight management.^19 44^ Specifically, participants feared that discussions might be overheard in open pharmacy environments and so advocated for private consultation spaces. This has also been identified in other studies as an important factor in the design of effective community pharmacy services in underserved communities.^67^

Pharmacists reported barriers such as insufficient time, heavy workloads and inadequate remuneration.^47^ Training gaps were another concern, with many pharmacists feeling underprepared due to limited education in nutrition and lifestyle counselling. Additionally, because some pharmacies did not have dedicated private consultation rooms as a resource, services which relied on person-centred and confidential conversations with patients were limited as it discouraged open dialogue on ways in which patients can improve their cardiovascular health.

Across most of our identified studies, it was unclear what occurs after screening or BI. Specifically, the referral routes, their uptake, and follow-up completion and outcomes thereafter. While some studies described mechanisms such as GP referral/communication, referral to diagnostic investigation, or referral into lifestyle and specialist support services,^16 19 38 52 58 59 62^ reporting of referral completion and longer-term follow-up outcomes was limited. This limits synthesis across studies, and so future evaluations should strive to report the referral mechanism, referral uptake and follow-up processes, including whether referrals were completed and any further outcomes.

Supporting resources played a crucial role in enhancing the effectiveness of pharmacist-led interventions. Educational materials, decision aids and electronic tools improved the quality and consistency of services. For example, Lemanska *et al* demonstrated increased adherence to recommended behaviours through the use of exercise DVDs, pedometers and dietary guides.^20^ Similarly, other recent studies within a pharmacy setting have also shown that embracing digital innovations, such as mobile applications and point-of-care testing devices, can improve service efficiency and accuracy, improve engagement and empower both pharmacists and patients. In particular, the use of advanced technologies that use artificial intelligence and data-driven analytics has considerable potential to personalise services for better health outcomes for patients.^68^ Additionally, in a related review, Merks *et al* proposed a national model for implementing a pharmacist-led NMS in Poland, highlighting the cost-effectiveness of such interventions for chronic disease management. The review supports the integration of structured educational resources and digital tools to improve adherence and health outcomes at scale.^69^

Our findings also indicated that the mode of service delivery could have an impact on outcomes. Face-to-face consultations were preferred for building rapport and addressing complex issues, whereas telephone follow-ups and digital platforms offered flexibility for ongoing support. Stewart *et al* successfully piloted a medicines and alcohol consultation programme combining face-to-face training with remote peer support via WhatsApp groups, achieving high participant retention rates and positive feedback.^50^ Offering flexible training schedules and on-site sessions could also address operational barriers faced by busy pharmacy teams, ensuring sustainability without hindering routine work.

### Strengths and limitations

A key strength of this review is the comprehensive synthesis of empirical evidence specific to the UK, which provides a contextualised understanding of the current landscape of community pharmacist-led cardiovascular screening interventions. This scoping review highlights the versatility of community pharmacies and their potential to deliver integrated care. The inclusion of both service user and pharmacists’ perspectives further strengthens the current feasibility findings. Several limitations should be borne in mind. First, the heterogeneity in study design, outcome measures and quality limited the comparisons across studies. Second, few studies systematically evaluated implementation fidelity or cost-effectiveness, which are crucial for informing policy. Third, while many studies assessed feasibility and acceptability, only some evaluated behavioural or clinical outcomes using objective measures at long-term follow-up.

## Conclusions

In conclusion, community pharmacies hold substantial promise for delivering additional brief cardiovascular healthcare screening interventions alongside vaccination services. While the integration of these services is facilitated by the accessibility and convenience of community pharmacies, the availability of consultation spaces and adequate staffing levels is critical for successful implementation. Supporting resources and appropriate delivery modes could further enhance the effectiveness of these interventions. Addressing the identified barriers will be essential to optimise community pharmacies in promoting public health and delivering cardiovascular-related and prevention services. Future research should focus on larger trials and longitudinal studies to evaluate existing community pharmacy-led services focusing on how existing services can be optimised and integrated to improve their uptake and produce better long-term health outcomes.

## Supplementary material

10.1136/bmjopen-2025-108381 online supplemental file 1

10.1136/bmjopen-2025-108381 online supplemental file 2

10.1136/bmjopen-2025-108381 online supplemental table 1

10.1136/bmjopen-2025-108381 online supplemental table 2

10.1136/bmjopen-2025-108381 online supplemental table 3

## Data Availability

Data sharing is not applicable as no datasets were generated and/or analysed for this study. All data relevant to the study are included in the article or uploaded as supplementary information.
