# Supplementary material for: Integrating cardiovascular healthcare screening into a community pharmacy vaccination service: a scoping review to identify opportunities for patient engagement and service expansion
Source: BMJ Open. 2026 Mar 23;16(3):e108381. doi: 10.1136/bmjopen-2025-108381 (PMC13034389; doi:10.1136/bmjopen-2025-108381)
Supplement: online supplemental file 1 [file bmjopen-16-3-s001.pdf]

Scoping Review

Search strategy

Search terms (Ovid medline/Embase/Web of Science/CINAHL Ultimate). For database CINAHL Ultimate and Web of Science replace with wildcards * or # or N or W.

1. (BLOOD ADJ PRESSURE ADJ MONITOR$3).TI,AB.
2. (AMBULATORY ADJ BLOOD ADJ PRESSURE ADJ MONITOR$3).TI,AB.
3. (AMBULATORY ADJ MONITOR$3).TI,AB.
4. CARDIO-CHECK.TI,AB.
5. (CARDIO ADJ CHECK).TI,AB.
6. CARDIOCHEK.TI,AB.
7. CHOLESTECH.TI,AB.
8. QRISK$1.TI,AB.
9. MECC.TI,AB.
10. (MAKE ADJ EVERY ADJ CONTACT ADJ COUNT).TI,AB.
11. (LIFESTYLE ADJ ADVICE).TI,AB.
12. (NHS ADJ HEALTH ADJ CHECK).TI.AB.
13. (HEALTH ADJ CHECK).TI,AB.
14. DIABET$2.TI,AB.
15. (T2 ADJ DIABET$2).TI,AB.
16. (SMOKALYSER ADJ TEST$1).TI,AB.
17. (ALCOHOL ADJ BRIEF ADJ INTERVENTIONS ADJ SCRATCH ADJ CARD$1).TI,AB.
18. (ALCOHOL ADJ SCRATCH CARD$1).TI,AB.
19. (KNOW ADJ YOUR ADJ NUMBER$1).TI,AB.
20. (LIPID ADJ CHECKS).TI,AB.
21. (LIPID ADJ MONITORING).TI,AB.
22. (LIPID ADJ ANALYSIS).TI,AB.
23. SPHYGMOMANOMETER.TI,AB.
24. MANOMETER.TI,AB.
25. (BLOOD ADJ PRESSURE ADJ DIAR$3).TI,AB.
26. ELECTROCARDIOGRA$2.TI,AB.
27. (HEALTH ADJ EDUCATION).TI,AB.
28. (HEALTH ADJ PROMOTION).TI,AB.
29. (SMOKING ADJ CESSATION).TI,AB.
30. DIET.TI,AB.
31. PHYSICAL ADJ ACTIVIT$3.TI,AB.
32. (ASSIGN ADJ SCORE$).TI,AB.
33. (TROPONIN ADJ TEST).TI,AB.
34. (HEALTHY ADJ HEART).TI,AB.
35. (BRIEF ADJ INTERVENTION$1).TI,AB.
36. (BRIEF ADJ SERVICE$1).TI,AB.
37. (REFERRAL ADJ PATHWAY$1).TI,AB.
38. (REFERRAL ADJ TOOL$1).TI,AB.
39. **1 OR 2 OR 3 OR 4 OR 5 OR 6 OR 7 OR 8 OR 9 OR 10 OR 11 OR 12 OR 13 OR 14 OR 15 OR 16 OR 17 OR 18 OR 19 OR 20 OR 21 OR 22 OR 23 OR 24 OR 25 OR 26 OR 27 OR 28 OR 29 OR 30 OR 31 OR 32 OR 33 OR 34 OR 35 OR 36 OR 37 OR 38.**
40. (PHARMACIST ADJ SERVICE$1).TI,AB.

41. (VACCINATION ADJ SERVICE$1).TI,AB.
42. (PHARMACEUTICAL ADJ CARE ADJ SERVICE$1).TI,AB.
43. (PHARMACEUTICAL ADJ CARE).TI,AB.
44. (PHARMACY ADJ SERVICE$1).TI,AB.
45. (COMMUNITY ADJ PHARMAC$3).TI,AB.
46. PHARMAC$3.TI,AB.
47. **40 OR 41 OR 42 OR 43 OR 44 OR 45 OR 46.**
48. CARDIOVASCULAR.TI,AB.
49. DIABET$2.TI,AB.
50. PREDIABET$2.TI,AB.
51. SEDENTARY.TI,AB.
52. OVERWEIGHT.TI,AB.
53. (OVER ADJ WEIGHT).TI,AB.
54. OBES$3.TI,AB.
55. PATIENT$1.TI,AB.
56. (SERVICE ADJ USER$1).TI,AB.
57. (ATRIAL ADJ FIBRILLATION).TI,AB.
58. ADULT$1.TI,AB.
59. HYPERTENSION.TI,AB.
60. (HIGH ADJ BLOOD ADJ PRESSURE).TI,AB.
61. (CHRONIC ADJ OBSTRUCTIVE ADJ PULMONARY ADJ DISEASE).TI,AB.
62. (CORONARY ADJ HEART ADJ DISEASE).TI,AB.
63. (HEART ADJ DISEASE).TI,AB.
64. (CARDIAC ADJ REHABILITATION).TI,AB.
65. (LOW ADJ SOCIOECONOMIC ADJ STATUS).TI,AB.
66. (LOW ADJ SOCIODEMOGRAPHIC ADJ STATUS).TI,AB.
67. (SOCIALLY ADJ DEPRIVED).TI,AB.
68. (DEPRIVED ADJ GROUPS).TI,AB.
69. (DEPRIVED ADJ AREA$1).TI,AB.
70. (INCOME ADJ DEPRIVED ADJ COMMUNIT$3).TI,AB.
71. **48 OR 49 OR 50 OR 51 OR 52 OR 53 OR 54 OR 55 OR 56 OR 57 OR 58 OR 59 OR 60 OR 61 OR 62 OR 63 OR 64 OR 65 OR 66 OR 67 OR 68 OR 69 OR 70.**
72. RCT.TI,AB.
73. (RANDOMI$4 ADJ CONTROL$3 TRIAL$1).TI,AB.
74. TRIAL$1.TI,AB.
75. (QUASI ADJ EXPERIMENT$2).TI,AB.
76. OBSERVATIONAL.TI,AB.
77. COHORT.TI,AB.
78. (SYSTEMATIC ADJ REVIEW).TI,AB.
79. (META ADJ ANALYSIS).TI,AB.
80. META-ANALYS*.TI,AB.
81. METAANALYS*.TI,AB.
82. (SCOPING ADJ REVIEW).TI,AB.
83. (RAPID ADJ REVIEW).TI,AB.
84. REVIEW ADJ OF ADJ REVIEWS.TI,AB.
85. (OVERVIEW ADJ OF ADJ REVIEWS).TI,AB.
86. META-ETHNOGRAPHY.TI,AB.

87. **72 OR 73 OR 74 OR 75 OR 76 OR 77 OR 78 OR 79 OR 80 OR 81 OR 82 OR 83 OR 84 OR 85 OR 86.**
88. WEIGHT.TI,AB.
89. BMI.TI,AB.
90. (BODY ADJ MASS ADJ INDEX).TI,AB.
91. (WAIST ADJ CIRCUMFERENCE).TI,AB.
92. (WAIST ADJ TO ADJ HIP).TI,AB.
93. BLOOD PRESSURE READING$1.TI,AB.
94. MILLIMETRES OF MERCURY.TI,AB.
95. (TOTAL ADJ CHOLESTEROL).TI,AB.
96. CHOLESTEROL.TI,AB.
97. GLUCOSE.TI,AB.
98. TRIGLYCERIDE$.TI,AB.
99. **88 OR 89 OR 90 OR 91 OR 92 OR 93 OR 94 OR 95 OR 96 OR 97 or 98.**
100. **39 AND 71 AND 87**
101. **39 AND 71 AND 87 AND 99**
102. **39 AND 47 AND 71 AND 87 AND 99**
103. **37 AND 45 AND 69 AND 85**
104. **39 AND 47 AND 71**
105. **39 AND 71 and 99**
