# Supplementary material for: Integrating cardiovascular healthcare screening into a community pharmacy vaccination service: a scoping review to identify opportunities for patient engagement and service expansion
Source: BMJ Open. 2026 Mar 23;16(3):e108381. doi: 10.1136/bmjopen-2025-108381 (PMC13034389; doi:10.1136/bmjopen-2025-108381)
Supplement: online supplemental table 1 [file bmjopen-16-3-s003.docx]

| **Table 1. Overview of characteristics of included studies** | | | | | | |
| --- | --- | --- | --- | --- | --- | --- |
| **First Author,**  **Year** | **Type of study,**  **Intervention mode,**  **Intervention** | **Study aim** | **No of participants & sample type** | **Key outcomes measured** | **Reported intervention content.**  **Who delivered by,**  **referral routes. Duration** | **Results and conclusions** |
| Albasri et al (2018) ^38^ | Cross-sectional  Online  Online Referral system | To identify factors linked with the referral of patients from community pharmacies to general practice within the first two weeks of starting an antihypertensive medication program. | N= 131,419  Sample: patients presenting to a community pharmacy in England with a newly diagnosed or with pre-existing hypertension who are starting new medication.  Females= 53%  Mean age= 65 years old  Predominately White= 85% | Factors relating to patient referral from the community pharmacy to General Practice within the first two weeks of beginning a new antihypertensive medication regime.  Referral occurrences | Exploring referral routes from pharmacy.  Any ‘referral’ occurrences  marked on the pharmacist-completed New Medicine Service  form.  Service duration: Not reported | During the first 2 weeks of starting a new antihypertensive medication, 5895 patients (4.5%) were referred to a GP by a pharmacist.  Patients reporting side effects from their new medication were most likely to be referred to their GP (adjusted OR 11.60, 95% CI 10.85 to 12.41). Other patient-reported factors significantly associated with referral included negative feelings towards the antihypertensive drug (adjusted OR 3.29, 95% CI 3.01 to 3.60) and reports of uncertainty of the efficacy of the new medication (aOR 1.50, 95% CI 1.35 to 1.68). In contrast, patients informed about their new medication by the pharmacist (aOR 0.45, 95% CI 0.39 to 0.52) and those taking their medications as prescribed (aOR 0.20, 95% CI 0.18 to 0.21) were less likely to be referred to their GP. Ethnicity nor age were significant predictors of pharmacy referral to general practice but females were more likely to be referred than males (aOR 0.85, 95% CI 0.80 to 0.91). Alpha-blockers were associated with pharmacy referral but not prescribed angiotensin II receptor blockers.  The study concluded that referrals from community pharmacies to general practice during the initiation of antihypertensive medications were driven primarily by patient-reported factors such as side effects, negative perceptions of the medication, and uncertainty about its effectiveness.  These findings highlighted the critical role of pharmacists in identifying and addressing early concerns among patients starting antihypertensive therapy, ensuring timely interventions and appropriate management. |
| Atkin et al (2020) ^28^ | Qualitative study involving semi-structured interviews and observation of routine practice including medication use reviews.  In-person  Explore the current services offered by pharmacies. Particularly, the Healthy Living Pharmacy framework. | Examine how a governmentality that focuses on the privatisation of risk translates itself within community pharmacy and to determine to what extent ‘the pharmacy gaze’ is being extended. | Observation: N= 14 community pharmacists  Sample: 10 North of England pharmacies  Interviews: N= 25 (16 men, 9 women)  Sample: people who drank alcohol and on multiple medicines;  Female= 36%  Mean age= 64 years old. | Patient perspectives on healthy living activities promoted by pharmacies including use of medicines, alcohol, pharmacy use, medicines review and the inclusion of alcohol as part of the review.  Observations to understand the working environment, recruitment of patients and conducting medicines reviews.  Qualitative data collected over a 5-year period. | Healthy living advice whilst conducting a medicines review.  Service duration: Not reported | Community pharmacists struggled to provide meaningful healthy living advice during medication use reviews, often reducing their role to an "essentialised enactment of consumerist healthcare."  The study concluded that adding public health roles to pharmacists' responsibilities without adequate support risks diluted the perceived value of these interventions. Instead of enhancing professional status, these "add-on" roles sometimes led to superficial engagement with patients. The findings demonstrated the need for better training, private consultation spaces, and integration of public health activities into pharmacists' core workflows to ensure meaningful and effective delivery of healthy living advice. This study highlighted the tension between expanding pharmacists' roles and the practical limitations of their working environments. |
| Barrett et al (2019) ^39^ | Cross-sectional survey  In-person  Blood pressure screening services | To examine the accuracy (calibration and validation status) of digital blood pressure (BP) monitors that are used within community pharmacy and assess the overall quality of BP screening services. | N= 500 pharmacies  Sample: Selected from community pharmacies across England. Community pharmacies contributing to the NHS business services authority.  Female= 50%  Mean age= Not reported | Number of pharmacies providing blood pressure screening and whether they use validated clinical metres. | Blood pressure device and service provision; associated training; instructions given to patients  Service duration: Not reported | Of the 500 surveyed pharmacies, 109 responded, with 61% (n=66) offering free BP checks to patients. Most of these pharmacies (97%; n=61) used automatic BP monitors, while only 3% relied on semi-automatic devices, and none used manual sphygmomanometers. However, only 61% of pharmacies reported using validated clinical meters, with 9% using monitors that had failed validation and 30% providing insufficient information about their device’s status. Additionally, 92% of respondents reported receiving some form of training, though the quality varied significantly. On average, each pharmacy screened between 1 to 10 people monthly, with annual screenings ranging from 10 to 2,000 individuals. Concerningly, many of the BP monitors in use were outdated, raising questions about their accuracy.  The study concluded that while BP screening services were widely available in community pharmacies, inconsistencies in device validation and training highlighted significant gaps in service quality. The variability in training methods – ranging from informal discussions to structured education – suggested a lack of standardisation, which could impact the reliability of BP measurements. Furthermore, the wide range of annual screenings (10 to 2,000) indicated inconsistent utilisation of the service. These findings highlighted the need for improved guidelines, regular equipment maintenance, and standardised training to ensure accurate and effective BP monitoring within community pharmacy. |
| Boardman & Avery (2014) ^59^ | Retrospective analysis  In-person  Weight management | Evaluation of a community pharmacy weight management intervention to support obese individuals to lose weight. | N= 281  Sample: Adults aged at least 18 years with at least one risk factor for coronary heart disease (hypertension, hyperlipidaemia, T2D, increased waist circumference (≥ 102cm in males, ≥88 cm in females; ≥90cm in Asian males, ≥80cm in Asian females) and a BMI of 30 to 38 kg/m².  Exclusion: Pregnant/breastfeeding women; those considered in too poor health  Mean age= 53 years old | Weight loss= kg  Waist circumference= cm  Blood pressure= mmHg | Pharmacist led weight management programme including approaches to motivate behaviour change in patients. Service use through GP referral or self-referral. Patients invited to attend a follow up session 2 weeks later and subsequent monthly meetings for a total of 12 visits. Content included calorie restricted diet plans and PA targets along with other health advice such as smoking cessation.  Service duration: 6 months | Among 281 participants, the programme included calorie-restricted diet plans, physical activity targets, and lifestyle advice such as smoking cessation, delivered over six months through GP referrals or self-referrals. At three months, participants achieved significant weight loss, with an average reduction of -3.07 kg (SD± 3.49) and a waist circumference decrease of -3.87 cm (SD± 5.01). Around 26 participants (approximately 10%) achieved clinically significant weight reductions of 5% or more. These outcomes were sustained at six months, with greater improvements observed, including an average weight loss of -4.59 kg (SD± 4.74), a waist reduction of -4.79 cm (SD ± 5.37), and notable decreases in systolic (-9.5 mmHg, SD± 20.1) and diastolic blood pressure (-4.7 mmHg, SD± 9.0).  The study concluded that community pharmacies could effectively deliver weight management programmes, leading to measurable reductions in weight, waist circumference, and blood pressure among participants. However, the programme faced challenges, including a high loss to follow-up, with 61% of participants dropping out at 3 months. Despite this, the findings demonstrated that such pharmacy-led interventions could contribute to sustainable health improvements for obese individuals. Retention issues need to be addressed though as well as ensuring adequate support mechanisms to sustain long-term adherence and outcomes. |
| Brown et al (2014a) ^14^ | Descriptive and  Semi-structured Interviews  In person  Healthy Living Pharmacies (HLPs) | Assess whether participation in a HLP scheme – including the number of participating pharmacies and services available as compared with pharmacies not participating within this scheme, has an impact on engagement.  Interviews with pharmacy staff to understand the factors contributing to successful delivery of HLP pharmacies. | Interviews N= 38 (from 32 pharmacies)  Sample: All community pharmacy staff participating in a HLP scheme.  N= 281 patients  Sample= patients attending the pharmacist-led weight management programme.  Female= 77%  Male= 23%  Mean age= Not reported | Number of patients seen for a particular service and where appropriate interventions are made. | HLP programme includes:  Medication Use Reviews (MURs) targeting respiratory conditions  Smoking cessation programmes  Alcohol awareness service  Emergency hormonal contraception (EHC)  Weight management service (26-week programme)  Service duration: 6 months | Healthy Living Pharmacies (HLPs) conducted more targeted Medication Use Reviews (MURs) for respiratory conditions and completed nearly all follow-up MURs. Over a third of these patients reported improvements in their asthma symptoms. HLPs also enrolled significantly more patients in smoking cessation programs, achieving higher quit rates at both four and 12 weeks compared to non-HLPs. Additionally, 70% of HLPs participated in alcohol awareness campaigns, compared to only 42.1% of non-HLPs. While the uptake of emergency hormonal contraception services remained unchanged, weight management services showed promise – specifically, the 26-week programme that included counselling sessions with HLP staff.  Overall, participation in the HLP program had a positive impact on pharmacy teams. Staff reported increased enthusiasm, a stronger sense of belonging, and a shift toward patient-centered care rather than primarily focusing on medications, and this has benefited service users. Further research is required to determine the impact and success of HLP approach. |
| Brown et al (2014b) ^15^ | In depth focused face to face interviews with pharmacists, and service users.  Online surveys with service users.  Alcohol screening and Emergency Hormonal Contraception (EHC) | Evaluate the acceptability of the results of an evaluation on the impact of the Alcohol Use and Disorders Identification Test (AUDIT) tool on alcohol use of those accessing emergency contraception within pharmacy and the feasibility of the provision of alcohol advice. | Pharmacist interviews: N= 14  Patient interviews: N= 22 interviews with low-risk drinkers  Online acceptability survey with the low-risk drinkers and an additional 53 risky drinkers.  “Risk” identified by the AUDIT tool – Low risk if scored 7 or less; Risky if score of 8-19  Mean age= 20 years old | Examine views on the  delivery of screening and advice, and to explore perspectives on the usefulness of the “teachable moment”. | Alcohol screening  using the AUDIT tool. The AUDIT questionnaire categorises levels of risk of drinkers.  The AUDIT increases awareness of levels of alcohol consumption in service users and the type of brief advice provided by pharmacists.  Service duration: Not reported | Among participants, 73% who received brief advice were drinking above recommended guidelines. The intervention was generally well-received, with service users finding it acceptable to discuss alcohol consumption with pharmacists and not perceiving it as embarrassing. However, pharmacist uptake of the service was low, with some reporting challenges such as insufficient time, unfamiliarity with the AUDIT tool, and competing priorities in busy pharmacy settings. Pharmacists who used the tool more frequently reported increased confidence in delivering the service.  The study concluded that while it was feasible for pharmacists to deliver alcohol screening and brief advice, the targeted approach limited participation, leaving some pharmacists feeling they lacked sufficient experience with the AUDIT tool. Although no significant differences were observed in total AUDIT scores between intervention and control groups, both groups showed a significant reduction in the AUDIT consumption sub-scale score over time. This suggested that even minimal engagement with the tool may encourage reflection on drinking habits. Future studies should address barriers such as training availability and consider how to integrate alcohol interventions into routine pharmacy practice effectively. However, findings showed that service users found it acceptable to be asked about alcohol consumption by pharmacists and did not find it embarrassing. |
| Chalati et al (2020) ^40^ | Survey questionnaire  In-person  Stop Smoking Service (SSS) and Emergency Hormonal Contraception (EHC) | Determine whether community pharmacy (CP) delivery of SSS and EHC service using levonorgestrel  met the needs of the population. Also, to explore whether such community pharmacy services were cost‑effective. | N= 42  Public health leads of 42 local authorities.  Mean age= Not reported | CP SSS= total reach out of needs; total success out of needs; quit rates (at 4, 52 weeks, and at lifetime); pharmacy reach and success out of needs, and pharmacy share in success.  EHC patient group direction= need for EHC, as well as uptake of EHC through CP. Outcomes from four RCT assessing observed pregnancy rates in women who used levonorgestrel within 120hr of unprotected sex.  Costs of both services and cost-utility analysis was conducted. | Stop Smoking Service and emergency hormonal contraception patient group.  Delivered by community pharmacist in local authorities.  SSS intervention also involved behavioural support  Service duration: 12 weeks | The study found that smoking prevalence was significantly associated with higher levels of deprivation, yet SSS failed to help 5% or more of UK smokers quit. Among those who set a quit date, only 49.9% self-reported quitting, and 68.5% of these were confirmed via CO-validation, representing just 34.2% of all quit attempts – lower than the government-recommended 35% CO-validated quit rate. Community pharmacy SSS provision per 25,000 population moderately correlated with deprivation (rho= 0.4) and ethnic minority proportions but not with smoking prevalence. Pharmacies in areas with higher needs dealt with more smokers setting quit dates annually and achieved a higher number of quitters per year (rho = 0.31, p = 0.046). For EHC, despite a higher concentration of pharmacies in areas with elevated teenage pregnancy rates, provision did not match demand, leaving gaps in service accessibility.  The study concluded that increasing community pharmacy involvement in SSS could enhance both reach and success rates, particularly in deprived areas with greater needs. However, challenges such as insufficient awareness of available services and financial constraints limited the potential impact. Regarding EHC, the mismatch between pharmacy distribution and need highlighted inefficiencies in service allocation. Cost-utility analyses from four randomised controlled trials assessing levonorgestrel use within 120 hours of unprotected sex showed no significant differences in observed pregnancy rates. Overall, while community pharmacies demonstrated potential for delivering public health interventions, addressing systemic barriers like resource allocation, training, and public awareness was deemed essential for optimising their contribution to reducing smoking and unplanned pregnancies. |
| Cheema et al (2018) ^46^ | Randomised Controlled Trial  In-person  Provision of information/education | Determine whether structured information provided verbally and in writing by community pharmacy on high BP will be linked to improved BP control and maintenance by patients. | N= 64 (31 in intervention arm, 33 in control)  Sample: Adults starting BP medication  Mean age= 65 years old (intervention), 60 years old (control); 55% female (intervention), 45% (control); 78% White ethnicity (intervention), 76% (control) | Patients’ awareness of BP risks; knowledge of new BP medicine.  Three readings of systolic and diastolic BP were recorded  for both intervention and control group participants during four visits at weeks 0, 2, 4 and 26. BP was recorded electronically by trained pharmacy staff using a British Hypertension Society (BHS)-approved Omron BP monitor. | Received personalised information sheets containing structured advice on BP and their anti-hypertensive medication that was based on the National Institute for Health and Care Excellence (NICE) guidance. These information sheets were distributed by a trained pharmacist during three face-to-face sessions at week 0, 2 and 4 over a period of 6 months.  Delivered by a trained pharmacist.  Service duration: Unclear | At the 4-week follow-up, the intervention group experienced a significantly greater reduction in Systolic Blood Pressure (SBP) compared to the control group. Specifically, the intervention group achieved an average reduction of 8 mmHg (95% CI: 2.1 to 13.3), while the control group showed a reduction of 6 mmHg (95% CI: 0.6 to 11.7). However, this initial benefit was not sustained at the 26-week follow-up, as both groups demonstrated similar reductions in SBP: the intervention group had a mean reduction of 4.5 mmHg (95% CI: 1.2 to 7.7), and the control group had a reduction of 5 mmHg (95% CI: 1.3 to 8.8).  The findings suggested that verbal advice provided by pharmacists was as effective as written advice in supporting reductions in both systolic and diastolic blood pressure among hypertensive patients starting new BP-lowering medications. However, the provision of structured verbal and written education was associated with a significant improvement in patients' knowledge of hypertension, which was sustained beyond six months. Despite this improvement in knowledge, it did not translate into better long-term blood pressure control. |
| Chua et al (2022) ^41^ | Survey developed from discrete choice experimental design  In-person  Community pharmacy-based CVD health check | Identify the preferences and value  for a community pharmacy led CVD health check for the public. The specific objectives were to (i) establish the importance of different attributes associated with a community pharmacy led CVD health check; (ii) quantify, financially, trade-offs between such attributes and the value that consumers have in accessing a community pharmacy led CVD health check; and (iii) predict the probabilities of uptake of different community pharmacy led CVD health checks. | N= 407  Sample: Adults within the community  Male= 57%  Mostly aged 25-44 (54%); mostly educated to degree level or above (65%) | Identifying the type of health check service desired | Examined whether a health check service is desired and the kind of health check service that service users would like to be offered.  Service duration: 30 or 45 minutes | The survey, completed by 407 adults, revealed strong public interest in accessing CVD health checks within community pharmacies, with service characteristics significantly influencing uptake. Key attributes such as the comprehensiveness of the check (e.g., lipid profiles and diabetes screening), duration (30 or 45 minutes), and cost were evaluated. Respondents were willing to pay an average of £20–£30 for a 45-minute comprehensive check, which included detailed assessments, compared to £10–£15 for a basic 30-minute check. The findings highlighted that convenience, accessibility, and trust in pharmacists were key drivers of preference, particularly among males (57%), younger adults aged 25–44 (54%), and those educated to degree level or higher (65%).  The study concluded that integrating advanced CVD health checks into community pharmacies could enhance preventive healthcare delivery, particularly in impoverished areas. However, barriers such as insufficient private consultation spaces and staffing levels could hinder implementation. While participants valued the accessibility of pharmacies, concerns about privacy during sensitive discussions, such as alcohol consumption or weight management, were noted. Overall, the study demonstrated the potential for community pharmacies to play a pivotal role in early detection and prevention of CVD, providing that structural and financial challenges are addressed. These insights could inform policymakers on designing feasible and appealing health check services tailored to public preferences. |
| Corlett & Krska (2015) ^16^ | Mixed methods design involving interviews and survey questionnaire  In person  Pharmacy NHS Health Checks | Determine the frequency of  lifestyle advice and referrals following a pharmacy NHS Health Check, attendance at general practices for follow-up, and views of attenders of the service and their self-reported lifestyle change. | N= 190, 66 of whom also completed a survey of their experiences, and 19 of these respondents also completed a qualitative interview  Sample: Adults from the community  Service user demographics (n=190)  Mostly aged 40-55 (80%)  Female= 58%  Ethnicity= White (53%)  Survey responder demographics (n=66):  Mostly aged 40 to 55 (77%)  Female= 79%  Ethnicity= White (70%) | Demographics data and perspectives obtained from interviews.  Pharmacists recorded whether advice was provided on alcohol intake, PA, smoking cessation and weight management, whether referrals to other healthcare professionals for follow-up or for lifestyle support were offered, and patient acceptability of these services. | Brief advice provided on alcohol intake, PA, smoking cessation and weight management. No information on how checks were done and by which tools.  Service duration: Not reported | Pharmacists most frequently provided advice on exercise, followed by weight management. Among the participants, 23% (25 individuals) of those who were overweight or obese accepted a referral for weight management, while 16% (18 individuals) opted for a physical exercise referral. A total of 66 service users (35% of the sample) completed and returned the survey. Most respondents were white (70%) and female (68%). Notably, the proportion of participants who self-reported high cholesterol, increase in waist measurements, BMI, or overall cardiovascular disease (CVD) risk was lower compared to the total population of service users who received a health check.  Almost all respondents viewed their health check as a positive experience, highlighting the accessibility and convenience of the pharmacy setting. Most felt they had sufficient time and opportunity to ask questions. Only two participants reported not understanding everything discussed, while six mentioned having unanswered questions. However, these queries often extended beyond the scope of a typical health check, which primarily focused on interpreting cardiovascular risk scores.  Most respondents (56; 86%) recalled receiving verbal information about lifestyle changes, but fewer (36; 55%) were given written materials. Forty-five participants (68%) reported making at least one lifestyle change: 31 (47%) increased their exercise levels, 30 (45%) improved their diet, three (5%) reduced their alcohol intake, and five (8%) reduced or quit smoking. One participant joined Weight Watchers, and another claimed to have lost weight. Additionally, 18 participants (28%) reported being advised to complete further testing, with 14 (22%) attending follow-up sessions.  Nineteen interviews were conducted to explore participants' experiences. Reasons for using pharmacies included their convenience and accessibility. Interviewees generally felt that the health checks met or exceeded their expectations. While much of the information provided was not new, participants found it useful as a prompt to consider lifestyle changes. Most participants did not report significant lifestyle changes, although two successfully quit smoking.  The study concluded that community pharmacies effectively delivered NHS Health Checks, with participants appreciating the accessibility and convenience of the service. However, concerns about privacy and the depth of advice were noted, as some participants felt the information provided lacked the detail offered during consultations with doctors or nurses. While two participants stopped smoking as a direct result of the intervention, most did not make significant lifestyle changes. The findings suggest that community pharmacies can play a pivotal role in promoting public health initiatives. To enhance service effectiveness and patient outcomes, improvements in training, resource provision, and addressing privacy concerns are necessary. |
| Cox et al (2019) ^57^ | Non-randomised prospective cohort study with between subject design  In-person  E-cigarettes | Exploratory study on the effectiveness of offering an E-Cigarette (EC) on smoking cessation within a community pharmacy setting in Hertfordshire compared with the usual care of Nicotine Replacement Therapy offered by pharmacies. | 115 smokers  Sample: Female= 74%;  Mean age= 46.37.  Self-reported smoker verified by CO 7 ≥ parts per million (ppm) and aged 18 years or over | Self-reported total smoking abstinence at 4 to 6 weeks follow up. CO validation was taken where possible to back up self-report claims. | E-cigarette  Nicotine Replacement Therapy  Service duration: Unclear | At 4 to 6 weeks follow-up, self-reported smoking abstinence was significantly higher among those using EC alone (23/37; 62.2%) and EC combined with NRT (8/13; 61.5%) compared to NRT alone (22/65; 34.8%). The odds of quitting increased by 3.23 for EC alone and by 3.14 for EC+NRT compared to NRT alone. These results suggested that e-cigarettes were more effective than traditional NRT in achieving short-term smoking abstinence in this setting.  The study concluded that offering e-cigarettes within community pharmacies could be an effective strategy for smoking cessation, as they demonstrated improved outcomes to NRT in the short term. For every one person who successfully quit using NRT, 3.23 quit using e-cigarettes within 4 to 6 weeks. However, the authors noted that further research is needed to determine whether these promising effects can be sustained over the long term. While the findings highlight the potential of e-cigarettes as a cessation tool, uncertainties remain regarding their broader implementation and effectiveness beyond the initial intervention period. |
| Dewsbury et al (2015) ^42^ | Survey questionnaire  (by post)  In-person  Health check services | Two specific aims: (1) to obtain information about the provision of public health services and community pharmacists’ perspectives on providing these services in England; and (2) to assess whether pharmacy ownership, pharmacist role and the number of Primary Care Trusts-reported commissioned pharmacy public health services influenced service provision and perspectives. | N= 778 community pharmacists in 16 areas across England and across 151 primary care trusts. 206 pharmacists responded to the questionnaire.  Sample: Male= 55.2%  Mean age= Not reported  Half the respondents had qualified within the previous ten years, two-thirds were managers, 10% owners and 62% were working in multiples (defined as > 6 pharmacies). Twenty-nine (14.1%) indicated their pharmacy was designated as a Healthy Living Pharmacy (HLP), but 34 (16.5%) did not know . The 29 HLPs were across areas with differing levels of commissioned services and 23 were in multiple ownership. | Perspectives of community pharmacists on services provided, decisions about services, support, promotion and future developments. | Service provision offered by pharmacies including blood pressure monitoring and smoking cessation as well as non-NHS-funded services such as influenza vaccination, weight management support, cholesterol testing and travel vaccination.  Service duration: Not reported | Pharmacists reported offering a broader range of services than what was reflected in NHS data. The number of self-reported services, however, did not correlate with the number of NHS returns. Their perspectives on service provision were not influenced by the number of services offered, pharmacy ownership, or Healthy Living Pharmacy (HLP) status, although HLPs were relatively few in number. Over 50% of pharmacies provided services such as smoking cessation, blood pressure monitoring, and supervised drug consumption. The most commonly offered service was emergency hormonal contraception (EHC).  Blood pressure monitoring was highly valued by customers but was rarely offered as an NHS-funded service. Pharmacists were motivated to provide public health services primarily for professional reasons rather than financial gain, especially in independent pharmacies. However, only 35% of pharmacists reported being personally involved in decisions about which services to offer, and less than 20% had used local public health reports to guide these decisions. While most pharmacists expressed positive attitudes toward public health services and anticipated their expansion in the future, opinions were mixed regarding local and national support for service promotion and delivery. Many respondents selected neutral responses when asked about the promotion, support, and future of pharmacy public health services. This neutrality may stem from a lack of awareness about local priorities, the content of relevant documents, and the potential impact of restructuring on service commissioning.  The diversity of services provided and the optimistic outlook among pharmacists regarding the expansion of public health services were critical factors for the future development of these initiatives. Greater involvement in decision-making processes and increased awareness of local community needs could empower pharmacists to better tailor services to their populations. To ensure the effective commissioning, provision, and advancement of public health services in community pharmacies, both national and local organisations must provide stronger support.  The study concluded that extending public health services without adequate training or preparation is inadvisable, particularly for interventions targeting alcohol consumption or behaviour change. While both intervention and control groups showed significant reductions in AUDIT consumption sub-scale scores, this was likely due to general awareness rather than the intervention itself. Pharmacists identified challenges such as lack of time, competing priorities, and insufficient familiarity with tools like the AUDIT questionnaire, which hindered service delivery. Conversely, frequent use of these tools increased pharmacists' confidence. The findings underscored the need for better training and support to enhance the effectiveness of public health roles in community pharmacies. |
| Dhesi et al (2022) ^60^ | Retrospective analysis  In-person  Medication Use Reviews (MUR) | Evaluate the effect of a ‘real-world’ respiratory-focused MUR + service that was conducted in the Milton Keynes area in the UK over a 2-year period targeting patients with asthma or COPD. | 1152 patients in PharmOutcomes who received the baseline MUR+ service  Sample: 191 patients with asthma and 81 with COPD attended a follow-up service. No participants had a combination of both.  Mean age of asthma cohort= 51 years old  Mean age for COPD cohort= 66 years old | Primary outcome= disease control  Factors influencing changes in asthma or COPD control.  Whether referral to a smoking cessation service was different between patients with asthma versus COPD.  Annual follow-up. | Medication Use Reviews + Asthma or COPD. Included review of patient demographic data, last GP/nurse review date, current medication  Including consultation or participation in a smoking cessation programme.  Any medication-related issues were entered into a standardised form. Inhaler technique and device cleaning were assessed.  Service duration: Not reported | Among asthma patients, significant improvements were observed in Asthma Control Test (ACT) scores, with the mean score increasing by +1.2 points (95% CI, 0.6 to 1.8; P < 0.01). However, no significant improvement was found in the mean COPD Assessment Test (CAT) scores for COPD patients, which changed by only −0.2 points (95% CI, −1.4 to 1.0; P = 0.77). Additionally, no significant differences in outcomes were observed based on the timing of follow-ups for either group.  The study concluded that the targeted MUR+ service demonstrated potential benefits in improving asthma control, particularly for patients with poorer baseline control, but did not yield meaningful improvements for COPD patients. The small sample size and limited clinical significance of the findings suggest that further research is needed to confirm these results and explore their broader applicability. While the service showed promise for asthma management, its impact on COPD control remains uncertain, highlighting the need for tailored approaches to address the distinct needs of these patient groups. |
| Dhital et al (2022) ^36^ | Focus groups  In-person; telephone  Alcohol treatment and recovery groups | Understand service users’ perspectives and experiences of relapse prevention medication, perspectives of a telephone behavioural modification intervention delivered by pharmacists, and contingency management to support acamprosate adherence following assisted alcohol withdrawal. | N= 26  One focus group for each site from the four regions was conducted: site 1 (London) = 10 participants; Site 2 (West Midlands) = 6; Site 3 (Wessex) =5 and Site 4 (Yorkshire & The Humber) = 5).  Sample: Mean age= 48 years old.  Of the 26 participants, 13 participants had been prescribed acamprosate previously and others had not.  Mainly white British or Irish, n= 23.  Female= 38% | Four focus groups to understand service users’ experiences of relapse prevention medication, a telephone behavioural modification intervention, and sustained use of contingency management to support use of acamprosate following assisted alcohol withdrawal. | Relapse prevention medication; telephone behavioural modification intervention (involving incentives to reinforce positive behaviours) using Contingency Management to support acamprosate adherence.  Service duration: 6 months | Many participants expressed concerns about the inconsistent and limited support available for acamprosate treatment, partly due to a lack of understanding about the medication. They also held strong negative views about the concept of Contingency Management, a strategy used to encourage adherence. Despite this, most participants were positive about the role pharmacists could play in supporting sustained acamprosate use through telephone-based interventions.  When exploring whether pharmacists could help patients adhere to alcohol relapse prevention medications, opinions were mixed. Some participants felt that adequate support was already provided by clinic keyworkers or GPs, although these claims were not backed by specific examples of frequent contact with such professionals. Others reported negative experiences, stating they received little or no after-care following assisted alcohol withdrawal from clinics.  Additionally, participants demonstrated a limited understanding of how acamprosate works, which hindered their ability to adhere to the treatment. This knowledge gap suggests that pharmacists, with their expertise, could play a valuable role in educating patients and supporting adherence to acamprosate. |
| Dhital et a (2015) ^47^ | Randomised controlled trial  In-person  Brief intervention (10 minutes duration) on alcohol intake. | Tests whether a brief alcohol intervention delivered by community pharmacists is effective in reducing harmful drinking among pharmacy service users at three-month follow-up compared to a non-intervention leaflet-only control condition. | N= 407 pharmacy service users from 16 community pharmacies  Pharmacies had to have an NHS contract and be within the London Borough of Hammersmith and Fulham area with a consultation room.  Sample: Adult participants who accessed services within the 16 participating pharmacies were included in the trial. 407 were randomised: 202 to BI, 202 to Control  Overall demographics: Mean age= 40 years old  Female= 45.7%  Ethnicity= White (53.8%) | Change in total Alcohol Use Disorders Identification Test (AUDIT) scores from recruitment to 3 months follow-up, and the proportions remaining hazardous or harmful drinkers (scoring 8 or higher on AUDIT) at follow-up.  Secondary outcomes were change in AUDIT subscales scores (for alcohol consumption, problems, and dependence), and general health status assessed using the EQ-5D(27) at follow-up. | Participants allocated to the brief intervention group were offered a discussion with the pharmacist of up to 10 minutes duration.  Content of the brief intervention focused on encouraging participants to think further about their drinking and whether they should reduce it, and discuss how if they were ready to do so.  Included a conversation on how drinking fitted in with their lives, explored ambivalence and provoked their personal evaluation of their drinking including any associated problems.  The conversation was closed by either the participant or the pharmacist providing a summary of the conversation. Participants were also given the “Units and You” booklet, a “Unit/Calorie Calculator Wheel” and an alcohol services leaflet.  Control: participants were not informed they were control participants, and immediately after the envelope was opened were provided a leaflet by the pharmacist. This was, entitled “Alcohol: The Basics” and included information about alcohol.  Service duration: 10 minutes | At three-month follow-up, there was no significant difference in total Alcohol Use Disorders Identification Test (AUDIT) scores between the groups, with baseline AUDIT scores of 11.93 (SD± 3.24) in the intervention group and 11.53 (SD± 3.19) in the control group, reducing to 11.80 (SD± 5.88) and 10.77 (SD± 5.54), respectively. Additionally, the odds ratio (OR) for the effect of the intervention on hazardous or harmful drinking was not statistically significant in either unadjusted or adjusted models. While both groups showed significant reductions in the AUDIT consumption sub-scale score, these changes were not attributable to the intervention itself.  The study concluded that extending community pharmacy services to include brief alcohol interventions without adequate training or preparation is unlikely to be effective for addressing harmful drinking or initiating behaviour change. Despite some reductions in alcohol consumption observed in both groups, the lack of a significant intervention effect suggested that simply providing brief advice might not be sufficient. The findings highlighted the need for more robust training and support for pharmacists to enhance their capacity to deliver impactful public health interventions targeting alcohol misuse. |
| Dhital et al (2013) ^17^ | Mixed methods: questionnaire and focus groups  In-person  Brief Intervention (BI) (alcohol) | This study aimed to evaluate: (1) pharmacists’ attitudes towards hazardous/harmful drinkers prior to training and after delivering BI, and change in attitudes between these time points; (2) alcohol knowledge before and after training, and following BI delivery and changes between time points; (3) experience of BI training; (4) if these variables were linked to the number of BIs delivered; and (5) the training and support needs of pharmacists. | N= 29 pharmacists  Sample: Pharmacists recruited from 27 sites within inner London.  Mean age= 38 years old  Mean time in community pharmacy 12 years (SD 10) | Pharmacists’ attitudes towards hazardous harmful drinkers (validated Short Alcohol and Alcohol Problems Perception Questions)  Pharmacists’ alcohol misuse and BI knowledge  Using three brief questionnaires covering (1) drinking limits (2) drinking behaviour patterns and consequences (3) alcohol metabolism; (4) alcohol BI; and (5) assessment and treatment for alcohol misuse.  Pharmacists’ experiences of using training pack and project support (validated questionnaire and focus groups) | A one-day BI training course lasting for 8 hours for pharmacists. Educational materials were developed from the Drinkless program.  Service duration: 8 hr training course for pharmacists | Nineteen pharmacists, classified as the "recruiter group," delivered a total of 139 brief interventions (BIs) over five months. Among them, two pharmacists conducted between 26 and 38 interventions, accounting for 46% of the total, while five pharmacists completed 6 to 12 interventions (32%), and 12 pharmacists conducted 1 to 5 interventions (22%). In contrast, 10 pharmacists did not deliver any interventions and were categorised as the "non-recruiter group."  Significant changes in alcohol-related knowledge were observed across three time points: pre-training, post-training, and post-BI (W = 17.24, d.f. = 2, *p*≤ 0.001). The median percentage alcohol knowledge score increased by 6 points from pre-training to post-training, a change that was statistically significant (*p* = 0.029) among the 21 pharmacists analysed. However, there was a notable decline in knowledge, with a median decrease of 20 points from post-training to post-BI, which was also highly significant (*p* ≤ 0.001).  Pharmacists demonstrated a significant improvement in their overall positive attitudes toward working with drinkers, with a median increase of 0.3 from pre-training to post-BI (*p* = 0.044). Additionally, there was a highly significant improvement in role adequacy, with a median increase of 1.3 (*p* = 0.001), and a significant reduction in role legitimacy scores, with a median decrease of 1.0 (*p* = 0.049). When comparing motivation levels, recruiter pharmacists exhibited significantly higher motivation to work with drinkers than non-recruiters both before training (U = 45, *p* = 0.021) and after delivering BIs (U = 20, *p* = 0.008). Recruiter pharmacists also reported significant increases in role adequacy (*p* = 0.004) and work satisfaction (*p* = 0.046) from pre-training to post-BI. Conversely, non-recruiters showed no significant improvements and experienced a significant reduction in role legitimacy (i.e., how they perceive their knowledge and competency) scores toward working with drinkers (*p* = 0.024).  The majority of pharmacists (n = 27; 93%) found the training pace adequate and reported that it met their overall learning needs. No significant differences were observed in how recruiter and non-recruiter pharmacists rated the BI training.  In conclusion, the study demonstrated that pharmacists without prior experience in delivering brief alcohol interventions can be effectively trained to do so. While there was a marked increase in alcohol-related knowledge immediately following training, this knowledge declined significantly by the end of the study, suggesting that knowledge retention may diminish over time without reinforcement. If brief alcohol interventions are implemented as a regular service, ongoing or additional training may be necessary to sustain pharmacists' knowledge and skills. |
| Douglas et al (2019) ^18^ | Mixed methods including  survey questionnaire with qualitative data  In-person  Nutrition education intervention for pharmacists | The aims of this study were to:  (1) undertake a preliminary mapping of Northern Ireland pharmacists’ attitudes and practices relating to diet-related health promotion and disease prevention.  (2) Develop and pilot a nutrition education intervention for pre-registration pharmacists. | N= 306 responses to questionnaires  Sample: 165 were community pharmacists from Northern Ireland  Females= 72%  Mean age= Not reported | Knowledge, Attitude, and Practice (KAP) scores at three time points | Two-day workshop to train pharmacists to deliver nutrition education.  Key objectives included highlighting the role of a pharmacist in nutrition and health and providing a comprehensive overview of current national nutritional policy recommendations as well as their practical application.  A spiral learning approach revisited the topics on day 3 to build on the consolidation of basic concepts.  Service duration: 2-day intervention for pharmacists | The assessment of Attitude and Practice questions revealed that most community pharmacists in Northern Ireland felt unprepared to deliver nutrition education effectively. Qualitative responses further highlighted that, while pharmacists acknowledged health promotion and disease prevention as integral to their professional role, they recognised significant gaps in their education and training in these areas.  Overall, community pharmacists perceived nutrition education as insufficient, a sentiment that was also evident in their practice. Although pharmacists valued the importance of nutrition in public health, a lack of confidence hindered their ability to implement effective nutritional practices. This lack of confidence likely stems from low self-efficacy, driven by gaps in knowledge and skills, which can be traced back to inadequate education and training opportunities.  Qualitative feedback indicated that pharmacists would welcome an educational intervention, particularly if it were delivered by independent nutritional experts in an interprofessional setting. Such training, focusing on key areas identified in the survey, could enhance the knowledge, attitudes, and practices of pre-registration pharmacists, ultimately improving their capacity to deliver nutrition-related services.  The study concluded that Northern Ireland community pharmacists generally lacked sufficient training and skills to effectively deliver nutrition-related interventions, which was reflected in both their attitudes and practice. However, qualitative feedback suggested that an educational intervention delivered by independent nutritional experts within an interprofessional framework would be well-received and could improve knowledge, attitudes, and practices among pre-registration pharmacists. This lack of confidence and readiness highlighted the need for targeted training programs to address these gaps and empower pharmacists to contribute more effectively to public health initiatives related to nutrition. |
| Elliot et al (2016) ^48^ | Randomised controlled trial involving a parallel group design.  In-person or by telephone  The New Medicine Service (NMS) to improve medicines adherence | The aim of this study was to evaluate the effectiveness of the NMS compared with normal practice in changing medicines-taking behaviour in community pharmacies in England. | N= 504 participants (NMS= 251; Usual care= 253)  Sample: Recruited from 46 community pharmacies in England.  Control receives normal practice  Female= 52%  Mean age= 59 years old | Self-reported adherence at 10 weeks from the initiation of the intervention.  Patients were followed up at 10 weeks which is expected to be the minimum time required to demonstrate any behavioural changes from the intervention.  Assessed by the Morisky Eight Item Medication Adherence Scale (MMAS-8) | Repeated follow up in the short term, to increase effective medicine taking for the treatment of a long-term condition.  A free service offering advice to use medicines safely and to best effect. Involves three consultation appointments of around 10 to 15 minutes where the pharmacist asks about adherence and experience with the medicine. Could be f2f or telephone-based. Pharmacist invites patient 121 consultation 7-14 days after initial prescription for new medication (“intervention”); “follow-up” 14-21 days after that  Service duration: 21 days | Patients were followed up at 10 weeks, which was considered sufficient time to observe behavioural changes. Adherence was assessed using the Morisky Eight Item Medication Adherence Scale (MMAS-8). The results showed that the NMS significantly increased the proportion of patients reporting adherence to their new medication, with an improvement of 10.2–70.7% compared to 60.5% in the usual care group. These findings were consistent across two adherence measures.  The study concluded that the NMS was effective in enhancing medication adherence for patients starting a new treatment for long-term conditions. While the cost to the NHS of paying pharmacists to deliver the NMS was offset by small reductions in other healthcare-related costs, the effectiveness of the service varied depending on pharmacy ownership and location, suggesting potential inconsistencies in implementation. Although the results were promising, the authors noted that further research is needed to confirm the validity of these findings and explore factors influencing the variability in outcomes. Overall, the study demonstrated the potential of community pharmacy-led interventions to improve adherence and patient outcomes. |
| Elliot et al (2017) ^62^ | Economic evaluation  In-person or by telephone  The New Medicine Service (NMS) to improve medicines adherence. | Cost effectiveness evaluation of the NMS compared with normal practice to change medicine-taking behaviour. | N= 504  Sample:  Female= 51.6%  Age= 59 to 60 years old  Hypertension= 49.4%  Type 2 diabetes= 18.8%  Asthma/COPD= 23.2% Antiplatelet/anticoagulant= 8.5% | Economic evaluation  The researchers collected outcomes at 10 weeks. | Face-to-face  consultation 7 to14 days later, with a follow-up 14 to 21 days thereafter. The whole period lasting 5 weeks.  Pharmacists’ will also ask about adherence at follow up points. Involves patient-centred identification of problems and targeted treatment and support or action.  Service duration: 21 days | Results showed that the NMS produced better patient outcomes than usual care, with a mean increase of 0.04 Quality-Adjusted Life Years (QALYs) per patient compared to normal practice. Additionally, the NMS was associated with reduced costs, averaging -£139 per patient, with probabilistic means of 0.05 QALYs (95% CI: 0.00–0.13) and -£144 (95% CI: -769 to 73). The incremental cost-effectiveness ratio was –£3166 (probabilistic mean: -£2638), indicating that the NMS dominated normal practice.  The study concluded that the NMS had a high probability (0.96) of being cost-effective compared to normal practice at a willingness-to-pay threshold of £20,000 per QALY. There was a 78% probability that the NMS would dominate normal practice by providing better health outcomes at lower costs. While additional costs were incurred through remunerating community pharmacists, these were offset by small reductions in other NHS contact-related costs in the short term. Overall, the findings demonstrated that the NMS not only improved patient outcomes but also reduced long-term healthcare costs, supporting its implementation as a cost-effective intervention for enhancing medication adherence in community pharmacy settings. |
| Evans & Wright (2020) ^56^ | Long-term service evaluation.  In-person  Waistaway: a Very Low Calorie Diet (VLCD) programme | Presentation of findings from an effective, long-term, community pharmacy-led weight management private service. | N= 1023  Sample: Adults aged 18 to 75 years old with over 6.35kg of excess weight and BMI of above 25  Female= 85.2%  Mean age= Not reported | Weight (kg)  BMI ((kg/m2) | Includes a weekly weight monitoring appointment. Initial assessment identified patient weight loss goals, medical conditions and past dieting history. The VLCD was provided on a weekly basis to support compliance and sustain adequate and necessary monitoring. Two different options including the flexible plan to lose less than 6.35 kg and the sure plan for those who need to lose more than 6.35 kg. Resources include food diary assessment, portion control, and partial food replacement.  Service duration: Unclear | Overall, the cohort achieved a mean percentage weight loss of 10.1%, with individuals diagnosed with type 2 diabetes or hypertension achieving similar results (10.4% and 10.6%, respectively). Among the 555 participants who adhered to the programme without discontinuation, 382 were followed up post-weight loss, showing a mean weight regain of only 1.4 kg over an average of 132 days, equating to a modest 1.8% weight regain.  The study concluded that the provision of a nutritionally complete VLCD through private community pharmacy services could significantly contribute to addressing the obesity epidemic without incurring costs to state-funded health systems. However, the service duration was not clearly defined, and the intervention relied on participant payment, raising questions about its inclusivity and sustainability in publicly funded healthcare models. Supported by trained pharmacy assistants, the programme demonstrated the potential for community pharmacies to deliver effective weight management interventions, though the authors proposed further research was needed to explore scalability and long-term adherence. |
| Farley et al (2017) ^52^ | Feasibility study  In person  Behavioural support  VS  self-help resources  (Halving calories consumption by two weeks). | Feasibility study testing whether community pharmacists can opportunistically recruit smokers into nicotine assisted smoking reduction services. | N= 68 service users recruited across 18 pharmacies.  Smokers not currently receiving therapy for tobacco dependence  Mean age= 44 years old  Mostly White ethnicity, started smoking around age 16, most had made a serious quit attempt previously | Feasibility of implementation of the smoking reduction programme in pharmacies.  Outcomes included: (1) percentage of pharmacists that agree to participate, and participating pharmacies that passed an assessment of competence (2) how many smokers recruited to the programme on a monthly basis (3) proportion of smokers who transitioned to a cessation pathway or who dropped out (4) fidelity of pharmacists to the treatment conditions (5) completion rates of monthly questionnaire and of those contacted by phone (6) NRT used by trial arm (7) number of adverse events (8) participants who recommend the intervention to other smokers. Qualitative outcomes include pharmacists’ and smokers views of the intervention. | Primary aim: To reduce daily cigarette consumption by at least 50%. In the behavioural support arm, pharmacists suggested that learning a new pattern of smoking would stop consumption increasing again by disrupting learnt associations between certain cues and smoking behaviour. 3 method of reduction: A timer method was used whereby participants used a timer (could be on mobile phone) to signal times where they could smoke. The time between smoking lengthened each time to help reduce smoking behaviour; Smoke-free period method divided the day into hours and participants agreed not to smoke in the designated smoke-free hours (which lengthened over time); Behavioural support, where participants returned at least 8 occasions (typically baseline, 2, 6, 10, 16, 22, 28 and 34 weeks) for further support after baseline and each session lasted for around 10 minutes.  In the self-help arm, the protocol was same as above except all information was provided on a written booklet.  Service duration: 9 months | The study recruited 68 smokers across 18 pharmacies, with participants randomised into either behavioural support or self-help resource groups. The programme aimed to reduce daily cigarette consumption by at least 50% over nine months, incorporating methods such as timer-based smoking schedules and smoke-free periods. However, the trial faced significant challenges, with only 20% of participants remaining engaged by the 12-month follow-up. On average, participants remained in the programme for six weeks instead of the intended nine months, highlighting issues with retention and adherence.  The study concluded that the trial design was unappealing to both pharmacists and participants, making a full-scale rollout of the programme unfeasible. Barriers included patient preferences for cessation rather than reduction, the complexity of paperwork, and reluctance to be randomised. Pharmacists were also deterred by bureaucratic enrolment processes, while smokers often did not return for additional support. These findings suggested that shorter, simpler programmes may be more acceptable in community pharmacy settings. Overall, while the concept of smoking reduction in pharmacies showed potential, significant changes to the design, delivery, and engagement strategies are needed to improve feasibility and uptake. |
| Firth et al (2015) ^29^ | Qualitative interview study  In person  Healthy Living Pharmacy framework | Explore the barriers towards implementation and progression of the Healthy Living Pharmacy framework, from the perspective of the pharmacy and commissioner, and to identify whether barriers and enablers of HLP perceived by pharmacies are shared with commissioners. | N=  11 community pharmacists  11 Healthy Living Champions (providers)  4 commissioners of HLPs  From HLPs in the North of England.  Sample: All those interviewed were working in Healthy Living Pharmacies. 4 were in areas of low deprivation; 4 in areas of medium deprivation; 3 in areas of high deprivation  Mean age= Not reported | Understanding perspectives of working in healthy Living Pharmacies. | HLP programme includes:  Medication Use Reviews (MUR) targeting respiratory conditions; Smoking cessation; alcohol awareness campaign; emergency hormonal contraception; weight management.  The main themes of the tiers of service are health promotion (Level 1), health prevention (Level 2) and health protection (Level 3).  Services are thus commissioned according to these activities with smoking, alcohol and weight management being key areas of focus.  Service duration: Not reported | Qualitative interviews revealed four key advantages of the Healthy Living Pharmacy (HLP) framework, as identified by providers, though these themes were not acknowledged by commissioners. Firstly, pharmacy staff reported enhanced professional development and capacity, which they attributed to the training provided through the HLP framework. Secondly, the study found that interactions between pharmacy workers and service users improved significantly, driven by the increased knowledge and expertise of staff, which was a result of the broader range of services offered at HLPs. Thirdly, the transition to becoming an HLP was seen as a motivating and encouraging process for pharmacy teams, fostering a sense of purpose and engagement. Lastly, the smoking cessation program was widely regarded as a particularly valuable component of the HLP framework.  However, several barriers to the implementation of the HLP framework were identified. These included insufficient financial incentives, and the significant time commitment required to deliver HLP services. Additionally, a lack of public awareness about the HLP framework limited patient uptake of relevant services, which in turn affected the financial viability of the programme for community pharmacies. Implementing health checks in deprived areas was also considered challenging, as recruitment processes and the checks themselves were seen as time-consuming. Moreover, many potential participants were ineligible due to pre-existing heart disease, further complicating efforts to expand the programme.  Firth (2020) reinforces the importance of addressing structural and organisational barriers to ensure the successful integration of HLP services into routine practice. The study highlights the need for better communication strategies to raise public awareness and emphasises the role of commissioners in supporting pharmacies through adequate funding and streamlined processes.  In conclusion, the HLP framework was viewed as a valuable initiative by providers, contributing to workforce development and motivating pharmacy teams to engage more proactively with patients on health-related matters. Despite its potential, challenges such as financial constraints, time pressures, and low public awareness hindered its full implementation. The authors proposed that addressing these barriers through targeted support, improved communication, and greater collaboration between providers and commissioners was essential to optimise the impact of the HLP framework and enhance its sustainability in promoting public health. |
| Fitzgerald et al (2015) ^43^ | Cross-sectional study  In person intervention  Community pharmacists’ role in reducing alcohol consumption | Understand the Scottish general public's  perspectives on the role and involvement of community pharmacists in reducing alcohol  consumption amongst service users and alcohol-related harm. | N= 1573 people completed the survey  Scottish adults sampled from the electoral register.  Mean age 57 years old.  59% male  98% White ethnicity | Health professions which could potentially advise on safer alcohol consumption (recommended alcohol consumption limits)  Areas of safer alcohol consumption on which pharmacists could advise.  Attitudes towards pharmacist involvement.  Demographics. | Advice and education on alcohol consumption provided by community pharmacists.  Service duration: N/A | Findings showed that 56% agreed that pharmacists could provide advice on safer alcohol consumption, with over two-thirds expressing confidence and trust in pharmacists discussing alcohol-related issues. However, 78% preferred discussing such matters with their doctor, and 64% were concerned about privacy within pharmacies. Around one-third of participants reported that a Fast Alcohol Screening Test (FAST) score indicated harmful or hazardous drinking, highlighting the potential for pharmacists to identify at-risk individuals.  The study concluded that while there is public acceptability for pharmacist involvement in reducing alcohol consumption, further research is needed to evaluate the efficacy of such interventions before widespread implementation. The findings suggested that pharmacists are seen as accessible and trusted healthcare providers, but concerns about privacy and the need for clear referral pathways to specialist services must be addressed. These results indicated that integrating alcohol interventions into community pharmacy services could be feasible, provided these barriers are overcome and pharmacists receive adequate training and support. |
| Holland-Hart et al (2021) ^53^ | Feasibility study employing mixed methods including interviews and descriptive analysis.  In person  Community-based pharmacy referral service to encourage earlier symptomatic referral for chest X-rays to check for lung cancer. | Testing the feasibility of community pharmacies in the rapid symptomatic diagnosis of lung cancer within socioeconomically deprived locations as well as to determine whether there is potential for a future RCT. It also aimed to identify preferences regarding potential awareness campaigns to promote such a service. | Interviews:  N=  4 patients; 7 pharmacy professionals; 1 GP for stakeholder interviews.  Four focus groups  N= 6 health care professionals participating in one focus group; 13 members of the public participating in three focus groups.  Sample: 4 female and 9 male public members.  Mean age= 64 years old  Conducted within area of high levels of socioeconomic deprivation in terms of lung cancer incidence and outcomes.  The public focus group included one group of smokers; one group of former smokers; and one combined group of current smokers and former smokers | Understanding the barriers and enablers to pharmacy referral for lung cancer symptoms in socioeconomically deprived areas.  Examined acceptability and feasibility of the pharmacy service and recommendations for encouraging symptomatic presentation to the pharmacy.  Demographic and clinical information including:  Age, Gender, symptoms, smoking status, history of COPD or asthma, whether the patient had previously spoken to health professional about their symptoms, length of consultation, whether the patient declined referral during consultation and whether smoking cessation advice was offered. Data regarding the number and outcome of chest X-rays and time to patients receiving the results were derived from patient’s hospital records. | The pharmacy referral service pathway entailed community pharmacists assessing patients with lung cancer symptoms and to refer eligible patients to hospital for a rapid chest X-ray based on NICE guidelines. This service was provided between May 2019 and March 2020.  Service duration: N/A | Participants generally viewed the pharmacy aspect of the service as acceptable and potentially feasible, reporting familiarity and ease of access to pharmacists as key enablers, particularly in deprived and rural populations. However, low referral rates were observed during the feasibility study, with barriers including a lack of awareness about the service and concerns regarding pharmacists' credentials to deliver it. The study highlighted the need for better promotion and integration with secondary care to improve uptake.  The study concluded that while the concept of a pharmacy-based referral service for chest X-rays was promising, a range of improvements are needed before wider implementation or testing in a randomised controlled trial (RCT). A joined-up and standardised approach to training and service delivery for pharmacists, counter staff, and primary and secondary care providers was deemed essential to ensure timely communication of chest X-ray results. Although the service showed potential, the low number of referrals emphasised the importance of raising public awareness and addressing operational challenges. Overall, the findings suggested that community pharmacies could play a valuable role in early detection of lung cancer, provided these barriers are addressed. |
| Jalal et al (2016) ^54^ | Prospective feasibility/pilot-controlled trial  In person or by telephone  Referral and  Motivational interviewing | Assess the potential impact of a pharmacy care intervention involving hospital pharmacy referral to community pharmacy services on outcomes and the use of motivational interviewing on adherence to secondary prevention medication among recently discharged coronary heart disease patients. | N= 71  Sample: Discharged  participants from a London Heart Attack Centre following treatment for a coronary event.  Mean age= Not reported | Adherence to cardiovascular medication | Intervention was designed to include elements of motivational interviewing and to be integrated into the existing NMS/MUR services so pharmacists could continue to claim for their work. Motivational interviewing consultation took place around 2 weeks after hospital discharge either f2f or via phone and lasted 15-20 minutes. discussion on how to improve protective cardiovascular medicine taking.  Service duration: 15 to 20 minutes | The intervention, integrated into existing services like the New Medicine Service (NMS) and Medicines Use Review (MUR), consisted of a 15–20-minute consultation conducted either face-to-face or via telephone. At 3 months, there was a statistically significant improvement in adherence in the intervention group (M=7.7, SD=0.56) compared to the control group (M= 7.0, SD= 1.85; p= 0.026). This difference persisted at 6 months, with the intervention group maintaining higher adherence (M= 7.5, SD= 1.47) compared to the control group (M= 6.1, SD= 2.09; p=0.004). However, no significant differences were observed in systolic blood pressure or LDL cholesterol levels, potentially due to missing data.  The study concluded that the intervention was both feasible and acceptable, demonstrating positive clinical outcomes and high patient acceptability. While the results indicated a significant impact on medication adherence, the authors cautioned against overinterpretation due to the pilot nature of the study and the need for further testing in larger trials. The findings suggested that integrating motivational interviewing into community pharmacy services could enhance adherence to secondary prevention medications for CHD patients, but mentioned that additional research was necessary to confirm its broader applicability and long-term effectiveness. |
| Jumbe et al (2019) ^55^ | Observational pilot study  In person  STOP (Smoking Treatment Optimisation in Pharmacies) intervention: Smoking cessation advice | Evaluation of the intervention  fidelity of simulated smokers (actors) to assess  smoker engagement and enactment of key intervention  components by STOP trained staff. | N= 5 community pharmacies  6 Actors  (i.e., simulated smokers)  Female= 50%  Age range= 22 to 58 years old  Never smoked, 4 being ex-smokers.  Sample: 20 pharmacy staff including counter assistant (10),  dispensing chemist (4),  Trainee pharmacist (2),  Pharmacist (2),  Pharmacist, technician (1)  Business manager (1).  Community pharmacies in North  East London Boroughs area | Pharmacy staff recruitment  Fidelity assessment outcomes; Visits to community pharmacies.  Quit rates | The STOP training programme involves training sessions on health consequences of stopping smoking, how to perform health behaviour change through modelling and role play, adaptation of STOP to local circumstances, and seeking local champions. Focused on teaching of BCTs and having a patient-centred approach  Service duration: Unclear | Findings demonstrated that pharmacy staff trained in STOP exhibited improved consulting styles and increased use of intervention materials, such as educational resources and tools for smoking cessation. Quantitative analysis of field notes confirmed the availability and use of these materials, while results highlighted enhanced knowledge and self-efficacy among staff in delivering smoking cessation advice.  The study concluded that the methods used to assess fidelity were effective in practice, providing evidence that key intervention components were enacted during interactions with simulated smokers. However, the service duration was unclear, and further testing in real-world community pharmacy settings is necessary to validate these findings. Overall, the study demonstrated that training programmes like STOP can enhance the quality of smoking cessation services, but highlighted that additional research was needed to refine and scale up such interventions for broader implementation. |
| Jumbe et al (2022) ^49^ | Pragmatic, parallel-group, controlled trial  In person  Complex intervention to improve uptake of the Smoking Treatment Optimisation in Pharmacies (STOP) programme  Vs  Usual practice as control | Evaluation of the effectiveness of a complex intervention to increase service uptake and retention of the STOP. | Participants:  N= 631 adult smokers in the STOP intervention  N= 641 adult smokers in the control  Pharmacy staff:  N= 37 advisors; 30 support staff in STOP in the intervention arm  N= 44 advisors; 7 support staff in control arm.  Community pharmacy staff  N- 60 community pharmacies involved  Mean age= 46 years old (intervention); 45 years old (control) | The primary outcome was the treated smoker throughput measured using routinely collected data provided by service commissioners including the number of smokers who participated in the stop smoking service, those who set a firm date and completed at least one consultation on or before the quit date.  Secondary outcomes include four-week retention rate; four-week quit rate; continuous abstinence; STOP intervention acceptability; Pharmacy staff self-efficacy; Smokers satisfaction of a pharmacist-led SSS. | A theory-based consultation skills training for pharmacy staff with environmental prompts (badges, calendars and behavioural cues).  Service duration: Unclear | No significant differences were found between the intervention and usual practice groups in terms of setting a quit date, retention rates, or quit rates. Specifically, 631 smokers in the intervention group set a quit date compared to 641 in the usual practice group, resulting in a rate ratio of 0.75 (95% CI: 0.46 to 1.23) after adjustments for site and prescription numbers. At the 4-week mark, retention rates were 68% (432/631) in the intervention group and 78% (500/641) in the usual practice group (odds ratio: 0.80, 95% CI: 0.41 to 1.55). Similarly, quit rates at 4 weeks were comparable, with 42% (265/631) in the intervention group and 43% (276/641) in the usual practice group (odds ratio: 0.96, 95% CI: 0.65 to 1.43).  Pharmacy staff responded positively to the intervention, with 90% (56/62) reporting that the training had improved their skills. Sixty-eight percent of staff stated they would strongly recommend the training to others, although no significant difference in self-efficacy for service delivery was observed between the intervention and control arms. Among service users, 53% (70/131) did not complete the 6-month follow-up assessment, highlighting potential challenges with long-term engagement. However, among those who completed the follow-up (55/61), satisfaction was high, with 90% reporting they were satisfied or very satisfied with the service. Additionally, all service users in the usual practice group (n=33) and all but one in the intervention group (n=27) indicated they would recommend the service to other smokers.  In conclusion, while the intervention led to high levels of retention and acceptable quit rates within the NHS pharmacy stop smoking service, it did not significantly enhance service throughput or outcomes compared to usual practice. Pharmacy staff valued the training and reported improvements in their skills, but these enhancements did not translate into measurable improvements in service delivery or smoker outcomes. The lack of impact on key metrics such as quit rates and retention suggested that other factors – such as systemic barriers, resource limitations, or external influences – may currently limit the capacity of community pharmacies to help smokers quit. Future efforts should focus on addressing these broader constraints and exploring additional strategies to optimise the integration and effectiveness of smoking cessation services in community pharmacy settings. |
| Khan et al (2013) ^64^ | Pre- and post- experimental design (5 months)  In person  Alcohol Brief Intervention | Pre- and  post-experimental design to: (1) assess uptake of the  community pharmacy alcohol BI service; (2) establish whether alcohol consumption for hazardous drinkers has changed based on  post-BI scores; (3) assess service users’ acceptability of the service; and (4) conduct a preliminary  economic evaluation of the service through establishing  whether pharmacy-based alcohol BI affected health and  social care costs, including lost employment costs, and  whether it was cost-effective. | N= 141  Participants (drinkers) from 26 community pharmacists from South London.  Sample:  Male= 63%  Reason for visit not alcohol-related= 48%  Age= mostly between 25 to 64 years old. | Alcohol use risk level  Overall week alcohol unit total.  Service utilisation data | Full BI from the pharmacist, based upon the Feedback, Listen, Advice, Goals and Strategies (FLAGS) technique. AUDIT-C tool was administered to identify hazardous drinkers. Following receipt of the service, all customers received an alcohol unit wheel calculator, information about specialist alcohol services, and were followed up 1 week and 3 months later to establish changes in alcohol use  Service duration: Not reported | Most service users (73%) who received the brief intervention (BI) reported drinking above recommended alcohol consumption guidelines. Overall, participants responded positively to the alcohol BI delivered by community pharmacists, with most indicating they would recommend the service to others. A preliminary cost analysis indicated that the community pharmacy-based alcohol BI was a relatively low-cost intervention and did not significantly impact the utilisation of other health and social care services.  Further, hazardous drinkers demonstrated statistically significant reductions in both the number of drinking days and the total units of alcohol consumed three months post-intervention. These findings align with the Khan (2013) study, which highlighted the potential effectiveness of alcohol BIs delivered by community pharmacists. Additionally, the study emphasised that the intervention was well-received and valued by service users, with some suggesting improvements such as better advertising of the service, reducing consultation length, and providing more detailed information during the session.  The study concluded that community pharmacy-based alcohol BIs were relatively low-cost and did not significantly impact the use of other health and social care services. Service users responded positively to the intervention, with many appreciating the advice and support provided by pharmacists. Despite these positive outcomes, participants suggested improvements, such as better advertising of the service, reducing consultation length, and providing more detailed information. Overall, the findings indicated that alcohol BIs delivered by community pharmacists are feasible, acceptable to customers, and may effectively reduce alcohol consumption among hazardous drinkers, though they mentioned that further research was needed to confirm long-term effectiveness and its potential for wider implementation. |
| Krska & Mackridge (2014) ^19^ | Mixed methods study involving interviews and survey questionnaire.  In person  Pharmacy-based alcohol screening and advice services. | Three aims including: (1) explore the views of community pharmacy staff, the general public and other stakeholders towards pharmacy-based alcohol screening and advice services (2) involve all relevant stakeholders in designing an acceptable and feasible pharmacy based alcohol screening and advice service (3) evaluate a pilot service from the user perspective. | N= 10 telephone interviews with users of pharmacy screening.  N= 5 pharmacist interviews  N= 4 pharmacist staff  N= 2 with other stakeholders.  N= 150 survey questionnaires completed by service users.  Sample:  High deprivation areas: 53%  53% aged between  36 to 65 years old.  Low-risk drinkers= 53% | Professional and public perspectives of the service.  Number of service users offered a screening  Screening scores and interventions offered. | Key elements of the service included:  pharmacy-specific promotional materials and methods to supplement standard materials, no targeted specific groups, with selection left to individual pharmacy teams, involvement of pharmacy support staff, using AUDIT-C as a prescreen, discussion of full AUDIT score to take place in private area, with pharmacist, and direct referral to the local alcohol treatment service (SATINS)  Delivered by community pharmacists  Service duration: Not reported | Survey participants expressed positive attitudes toward pharmacy-based alcohol services, with 80% (120) reporting they were comfortable or very comfortable discussing alcohol consumption with a pharmacist. This level of comfort was comparable to discussions about smoking (127; 85%) and diet (120; 80%). The most highly regarded services included supporting individuals in reducing their alcohol consumption (138; 92%), providing written advice (136; 91%), offering information about additional services (135; 90%), and facilitating referrals to other services (134; 89%). In contrast, fewer participants viewed the provision of medications (117; 78%) or verbal advice (117; 78%) as beneficial. Further, age, gender, and drinking status did not significantly influence these perceptions. Most survey participants (127; 85%) were comfortable or unconcerned about pharmacists providing advice on safer alcohol consumption when requested, but only 66% (99) felt okay with pharmacists proactively raising the topic, whereas pharmacists themselves preferred responding to customer requests.  Key factors identified by respondents as important for encouraging the use of pharmacy alcohol services included privacy (144; 96%), confidentiality (141; 94%), a friendly pharmacist (143; 95%), and well-trained staff (123; 82%). Concerns primarily centered around the fear of conversations being overheard in open pharmacy environments rather than concerns about staff disclosing personal information. Pharmacist interviewees emphasised the need for adequate private consultation spaces, as the open layout of many pharmacies posed a barrier to delivering alcohol-related services effectively. Additionally, long waiting times (120; 80%) and the busyness of pharmacies (102; 68%) were highlighted as significant issues by both the public and pharmacy staff, underscoring the importance of involving counter staff in screening processes to alleviate workload pressures on pharmacists.  Stakeholders, pharmacists, and the public all agreed that training was essential to ensure staff had sufficient knowledge and that pharmacy services were integrated into broader healthcare systems. Promotion of services was also deemed critical, with participants preferring a mix of promotional methods. Posters in GP surgeries (129; 86%) and pharmacy windows (114; 76%) were the most preferred options, while advertising in places where alcohol is sold received less support, regardless of participants’ drinking status. These findings highlight the need for tailored promotion strategies to maximise service uptake and engagement.  This study demonstrated that while pharmacy-based alcohol services are viewed positively by the public, several barriers, such as concerns about privacy, lack of private consultation spaces, and high pharmacy workloads, must be addressed to optimise service delivery. Effective promotional strategies and staff training were proposed to be essential to optimise service uptake and ensure successful integration into routine care. By addressing these challenges, community pharmacies can play a vital role in supporting public health initiatives aimed at reducing alcohol-related harm, which should enhance the accessibility and impact of preventive healthcare services. |
| Lemanska et al (2019) ^20^ | Mixed methods design  In person  Physical activity lifestyle health assessment and home-based programme | Assess the feasibility and acceptability of a community pharmacy lifestyle intervention to improve physical activity and cardiovascular health of men with prostate cancer, and to refine the interventions based on findings. | N= 116  Male participants selected from 9 community pharmacies who completed treatment for non-metastatic prostate cancer.  Sample:  Mean age= 70 years old  Ethnicity= 98% White | Rate of participant recruitment  Consent  Retention and adverse events | Personalised lifestyle intervention that included exercise, nutrition and psychosocial elements, guided by a functional and anthropometric personal assessment. Men received tailored home-based exercise and dietary advice with a support pack (including an educational DVD, booklet, pedometer and resistance exercise bands). Men were supported with two phone calls from a pharmacist and a follow-up appointment at 3 months after the first assessment to reassess goals, reinforce personalised lifestyle advice and support sustainability of lifestyle change.  Service duration: 12 weeks | The community pharmacy-based lifestyle intervention was found to be both feasible and acceptable to both providers and men who had received a diagnosis of prostate cancer. The study reported a 15% attrition rate, with follow-up completed by 86% of participants at one week and 71% at six weeks. These findings confirm that such an intervention is well-received by men living with or beyond cancer and demonstrate its feasibility across three different community pharmacies. A key strength of the recruitment process was the use of invitation letters sent by oncology consultants. The primary reasons for non-participation included being too busy, feeling unwell, or already being physically active.  Qualitative insights from participant interviews and focus groups with pharmacy teams revealed a common misconception among participants, that they assumed pharmacists had access to their NHS hospital and primary care records. This highlighted the need for clearer communication about data privacy in future interventions.  The study highlighted the potential for community pharmacies to play a vital role in supporting cancer survivorship initiatives within primary care. By offering accessible, personalised lifestyle interventions on the high street, pharmacies can contribute significantly to improving health outcomes. New evidence from this research suggested that pharmacy-led lifestyle interventions could benefit men living with and beyond prostate cancer by promoting healthier lifestyles.  Participants showed improvements in physical activity levels, other lifestyle factors, and cardiovascular risk scores. However, the study also noted that additional support may be necessary to help participants sustain these benefits over the long term.  The study concluded that the community pharmacy-based lifestyle intervention was both feasible and acceptable to both providers and participants. It highlighted the potential of community pharmacies to deliver accessible, personalised health interventions for cancer survivors, particularly in underserved areas. However, challenges such as misconceptions about pharmacists' access to NHS records and the need for better promotion of services were noted. Qualitative feedback emphasised the importance of tailoring interventions to individual needs and ensuring adequate follow-up support. Overall, the findings provided strong evidence for the role of community pharmacies in supporting cancer survivorship initiatives, though they stated that further research was required to refine and sustain the intervention’s impact over time. |
| Lemanska et al (2022) ^58^ | non-randomised, single-group study  In person  3-month lifestyle intervention: TrueNTH Exercise and Diet | To report patient activation, which is the knowledge, skills, and confidence in self-managing health conditions, and patient-reported outcomes of men after prostate cancer treatment from a community pharmacy lifestyle intervention. | N= 116  Male participants selected from 9 community pharmacies who completed treatment for prostate cancer.  Same population as above study.  Mean age= 70 years old | 26 prostate cancer-related functional outcomes measured using the Expanded Prostate Cancer Index Composite Short Form (EPIC-26) tool  Quality of life was evaluated with the Euro quality of life 5-dimension 5-level (EQ5D-5L) questionnaire.  Diet was assessed with the 14-item Mediterranean Diet Adherence Screener (MEDAS) questionnaire.  Self-reported physical activity was assessed by the Godin  leisure-time exercise questionnaire (GLTEQ)  Knowledge, role and confidence in managing one’s own health was measured by a 13-item version of the Patient Activation Measure (PAM). | Personalised lifestyle intervention that included exercise, nutrition and psychosocial elements, guided by a functional and anthropometric personal assessment.  Men received tailored home-based exercise and dietary advice with a support pack (including an educational DVD, booklet, pedometer and resistance exercise bands).  Men were supported with two phone calls from a pharmacist and a follow-up appointment at 3 months after the first assessment to reassess goals, reinforce personalised lifestyle advice and support sustainability of lifestyle change.  Service duration: 3 months | Significant improvements were observed in patient activation, measured using the Patient Activation Measure (PAM), at the 3-month mark. The PAM score increased by an average of 4 points, from 62 (95% CI 59 to 65) to 66 (95% CI 64 to 69), a change that was both statistically significant (p = 0.001) and clinically relevant, sustained at six months (p=0.008). Physical activity levels also improved significantly, with Godin Leisure-Time Exercise Questionnaire (GLTEQ) scores rising from 36 (95% CI 29 to 43) at baseline to 42 (95% CI 37 to 48) post-intervention (p = 0.010).  In addition to physical activity, participants demonstrated improvements in dietary adherence, as assessed by the Mediterranean Diet Adherence Screener (MEDAS), and other cardiovascular risk factors. However, the study highlighted the need for ongoing support to sustain these benefits in the long term. Qualitative feedback indicated that participants valued the accessibility and convenience of the pharmacy-based intervention but noted challenges such as misconceptions about pharmacists’ access to NHS records. Overall, the findings underscored the feasibility and acceptability of community pharmacies in delivering lifestyle interventions to improve health outcomes for prostate cancer survivors, providing a foundation for broader implementation and further research. |
| Lyons et al (2016) ^50^ | Parallel group randomised controlled trial  In person  The Medicines Advice Service intervention | Test the effectiveness of a tailored,  pharmacist-led centralised advice service to  improve adherence to patients on established  medications. | N= 677 (340 in intervention; 337 in control)  Patients prescribed at least one oral medication for type 2 diabetes and/or lipid regulation  Intervention: Mean age 70 years old  39% female, 96% White  Control: Mean age 70, 44% female, 97% White | Self-reported adherence to medication at 6-month follow-up, collected via a postal questionnaire.  Prescription refill adherence, lipid and glycaemic control and patient satisfaction | The intervention comprised two tailored telephone consultations with a pharmacist, 4–6 weeks apart, plus a written summary of the discussion and a medicines reminder chart. A medicines reminder chart, posted to the patient after the first consultation.  Service duration: 3 months | Ninety-two percent of the intervention group (n = 310) completed the intervention. However, 18 participants (5%) did not receive either telephone consultation, and an additional 12 received the first consultation but failed to complete the follow-up. The most common reasons for non-completion included not answering multiple calls or failing to respond to messages to arrange appointments. The median duration of follow-up consultations was 5 minutes and 36 seconds, with a median interval of 35 days (IQR 28–41 days) between consultations.  At both four weeks and six months post-intervention, the intervention group demonstrated significantly better self-reported adherence (defined as taking ≥90% of medication in the previous seven days) compared to the control group across all analyses.  Analysis of pharmacy dispensing records revealed that over a longer follow-up period, averaging 434 days, 29% of the intervention group were categorised as non-adherent (<90% of medication available), compared to 40.6% (127/313) in the control group. This resulted in an odds ratio (OR) of 1.60 favouring the intervention group (95% CI 1.14 to 2.24).  Six months after the intervention, twice as many participants in the intervention group achieved HbA1c levels below 7% (66.7%, n = 16) compared to the control group (31.3%, n = 5). Although this difference approached statistical significance (p = 0.061), it did not reach the conventional threshold.  Overall satisfaction with the Medicines Advice Service was high, with 91.8% (n = 245) of participants reporting they were satisfied. Only four participants (1.5%) expressed dissatisfaction. Two did not provide a reason, while one participant stated the service was unnecessary as they had no concerns about their medications. Another participant criticised the pharmacy’s services in general rather than the Medicines Advice Service specifically.  The tailored intervention, delivered by pharmacists through spoken advice via telephone and written information sent by post, was well-received by patients. It proved effective in significantly improving medication adherence among participants in a mail-order pharmacy cohort. |
| Mackridge et al (2015) ^21^ | Mix methods study including ethnographic observation and interviews.  In person  alcohol screening and intervention service | This study aimed to develop and apply a model for in-depth scrutiny of CP-based screening and intervention services with feedback to service providers to support development of best practice. The study was undertaken using alcohol identification and brief advice (IBA) services in North West England as a case study | N= 5 interviews with service users  Recruited from 13 pharmacies.  N= 3299 observations  N= 9 attending consultations.  N= 16 follow-up telephone interviews at 2 weeks and 14 of these participated in a further interview 3 months later.  Mostly aged 55 to 64 years old (interviews) | Perspectives from interviews with service users  Observations of interactions at the counter.  Field notes documented. | IBA consultations  Service duration: Unclear | The findings lend further support to the feasibility of implementing an alcohol Identification and Brief Advice (IBA) service within community pharmacy (CP) settings. The feedback model presented offers a practical and potentially valuable framework for evaluating service delivery and driving improvements. Expanding this approach to other services in CPs could help identify key factors contributing to successful implementation and inform the development of best practice models. However, integrating the alcohol IBA service into existing workflows proved challenging for staff. Restrictions associated with commissioned services exacerbated these difficulties. The service was not consistently available across all pharmacies, primarily due to the limited availability of trained staff. Additionally, caps on the number of service episodes allowed per week or month meant that once these limits were reached, the service had to be suspended for the remainder of the period. Such interruptions hindered efforts to promote the service effectively and integrate it into routine practice. Some staff also reported discomfort discussing alcohol consumption with customers, perceiving it as a sensitive topic. This discomfort often led to missed opportunities to initiate conversations, such as during the purchase of products like hangover remedies. This was a challenge similarly observed in chlamydia screening services. Interestingly, service users did not express concerns about discussing alcohol in the pharmacy setting, which is in line with findings from other studies. Addressing staff confidence, attitudes, and skills through targeted training was identified as a potential solution to overcoming these barriers.  Key facilitators for successful implementation included fostering staff confidence and competence, appointing service champions, obtaining necessary materials, and supporting colleagues – via utilisation of promotional materials and private consultation spaces. Despite these facilitators, the overall impact of staff-customer interactions on service outcomes was limited, highlighting the need for further refinement and development of such interventions. |
| Madden et al (2020) ^30^ | Semi-structured interviews  In person  Discussion of AUDIT-C screening scores as part of medicines review | To better understand patient views on the appropriateness of alcohol as a subject for discussion in medication reviews in community pharmacy. | N= 25  Service users who drank alcohol twice a week or more often – identified by AUDIT-C tool as Risky drinkers  64% male  Mean age= 63 years old  100% White ethnicity | Perspectives on discussion of risk of drinking behaviour as part of medicines review. | Conversations around drinking status using the shortened form of the Alcohol Use Disorders Identification Test (AUDIT-C).  Service duration: Not reported | Some patients reported feeling hesitant to disclose their medication use or alcohol consumption during consultations with pharmacists, fearing they might be perceived as engaging in inappropriate behaviour. Others expressed concerns that discussing alcohol with pharmacists could lead to judgment or advice to abstain from drinking altogether. Many participants found it challenging to contextualise the general healthcare advice on alcohol limits into personally meaningful terms. Advice that was perceived as irrelevant or overly critical was met with resistance and considered unwelcome.  These findings highlighted important considerations regarding the willingness of patients to engage in discussions about alcohol during medication reviews with pharmacists. Patients’ perceptions of the pharmacist’s role, the nature of their relationship with the pharmacist, and their prior experiences with alcohol-related conversations are likely to influence their openness to such discussions. Enhancing pharmacists’ understanding of how patients conceptualise alcohol and ‘problem drinking’ could improve the way alcohol-related topics are addressed within medication review services. |
| Madden et al (2021) ^31^ | Qualitative: co-design workshop to co-produce drinking intervention.  In person  Discussion of alcohol as part of medicines review. | Explores sensitivities in discussing alcohol and views on the legitimacy of the Medicines and Alcohol Consultation intervention concept at a pharmacy-user intervention co-design workshop. | N= 14  Service users who drank regularly and took medications for long term conditions. Recruited from UK community pharmacies.  Mean age= 64 years old | Participants discussed and voted (using a red, green or amber ‘traffic light’ system) on how to introduce the subject of drinking, provide the rationale and hold a conversation in this particular context. | Drinking intervention to encourage discussion on risky drinking behaviour during medicines review.  Service duration: N/A | Participants unanimously agreed that discussing alcohol in the context of medication reviews was acceptable, provided pharmacists clearly connected the conversation to the individual’s specific medications and health conditions. Importantly, participants pointed out that the tone of the discussion needed to remain non-judgmental and avoid coming across as lecturing. The workshop highlighted that the concept of alcohol as a drug – and more specifically, the use of the term “drug” itself – requires careful handling during consultations to prevent unintended negative reactions. This sensitivity was noted despite the general perception that discussing alcohol aligns well with the goals of medication reviews. At various points during the workshop, the traditional focus on pharmacists providing safety advice about immediate alcohol-medication interactions overshadowed broader discussions about the long-term risks of alcohol consumption for chronic conditions.  Participants expressed strong support for reframing how alcohol is addressed by community pharmacists. This shift involves moving away from viewing alcohol solely as a lifestyle choice and instead emphasising its direct relevance to medication safety, effectiveness, and the management of underlying health conditions. Such an approach was seen as a positive and necessary step in enhancing the integration of alcohol-related discussions into routine medicines reviews. |
| Morrison et al (2013) ^65^ | Non-randomised prospective evaluation  In person  Counterweight weight management programme adapted for delivery in community pharmacies as part of Keep Well Project. | Evaluate the effectiveness of the Counterweight weight management programme when delivered through community pharmacies in achieving clinically significant weight loss. | N= 458  Overweight or obese adults aged 40 to 64 years old.  75% female  Mean age= 54 years old  Mean BMI= 36.0 kg/m² | Clinically Significant Weight Loss: ≥5% weight loss from baseline weight and at 12 months.  This threshold is considered meaningful for health benefits including risk of cardiovascular disease, improved glycemic control, and better lipid profiles.  Weight loss (kg) over time and percentage of weight loss relative to baseline.  Attendance rates. | Intervention components included:  Patient Education:  Pharmacy staff delivered education on weight management and behaviour change strategies.  Information was communicated using resources such as patient booklets, desktop flip charts, and training manuals.  Weight Loss.  Involved either a prescribed eating plan or a goal-setting approach.  As patients progressed, the focus shifted to weight loss maintenance and preventing weight regain.  Behavioural Support:  Emphasis was placed on strategies to sustain long-term weight loss, including adherence to dietary goals and physical activity recommendations.  Follow-Up Appointments:  Patients were encouraged to commit to 9 appointments over 12 months , including:  6 initial appointments (10–30 minutes each).  Follow-up visits at 6, 9, and 12 months. | The programme aimed to achieve clinically significant weight loss (≥5% of baseline weight) among overweight or obese adults. Between March 2009 and July 2012, 458 patients were enrolled, with a mean age of 54 years (SD 7.4) and a mean BMI of 36.0 kg/m² (SD 5.9). Of the 314 patients who were followed up for at least 12 months, 32 (10.2%) achieved the target weight loss of ≥5% on an intention-to-treat basis, representing 41.6% of those who attended at 12 months. The mean weight loss at 12 months was 4.1 kg, and using the Last Observation Carried Forward (LOCF) method, 15.9% of participants achieved the target weight loss within 12 months.  The study concluded that the Counterweight programme delivered in community pharmacies is as effective as primary care-based weight management programmes, achieving clinically significant weight loss in 10% of participants at 12 months. Despite these positive outcomes, the study highlighted challenges such as high attrition rates, with only 24.5% of participants attending the final follow-up. The findings suggested that while community pharmacies can play a valuable role in weight management, strategies to improve retention and attendance were necessary to enhance long-term success. |
| Nazar et al (2016) ^37^ | 2 focus groups  In person  Healthy Living champions (HLCs) as part of the Healthy living pharmacy project. | To investigate the impact of innovative networking opportunities in supporting HLCs to  function within their role and to explore the network’s potential in promoting sustained HLP participation. | N= 20  Portsmouth Healthy Living Champions from 33 Healthy Living Pharmacies | Perspectives generated from discussions within focus groups around HLP meetings. | Healthy Living Pharmacy initiative  Healthy Living Champions.  Service duration: N/A | The Healthy Living Pharmacy (HLP) initiative has proven to be an effective platform for enhancing the role of community pharmacies in delivering health and well-being services, primarily through the better utilisation of pharmacy staff. This study specifically identified several benefits for Healthy Living Champions (HLCs) who attended networking meetings facilitated and funded by the local commissioner of the service:  *Enhanced Motivation*: HLCs felt enthused and returned to their workplaces more motivated to continue in their roles.  *Collaboration Opportunities*: The meetings provided a forum for HLCs to share ideas and initiate collaborative health promotional activities outside of their regular work.  *Improved Training*: Training sessions during the meetings enhanced the HLCs' understanding of their role in public health services. However, the study also highlighted several barriers:  *Communication Gaps*: The absence of a system for disseminating meeting details and facilitating communication between meetings led to variable HLC attendance.  *Administrative Challenges*: Poor communication and administrative issues were identified as significant barriers to the progress and success of the meetings.  To address these challenges, the HLCs suggested forming a committee to take on leadership roles in managing communications and administrative functions. Additionally, implementing a communication tool that serves the desired functions, as mentioned by the HLCs, and forming a group committee to handle administrative tasks are the next steps towards consolidating the sustainability of the HLP initiative through the motivation and active contribution of HLCs.  The HLP project was introduced to improve patient outcomes by increasing the pharmacy’s role in promoting better health, preventing disease, and managing long-term conditions. Evaluations of the project across the UK showed that the project fostered a strong community of participating pharmacies and had benefited patients via the services provided. |
| Poole et al (2019) ^32^ | Qualitative study: semi-structured telephone interviews  In person  Multi-component lifestyle intervention | Phase II feasibility study of a lifestyle intervention delivered by nine community pharmacies in the UK to inform a larger efficacy study. Qualitative interviews explored how men experienced the intervention and these data are presented here. | N= 33  Males with prostate cancer  Mean age 72 years old  Mostly aged between 70-79 years old (55%) | Framework analysis to identify teachable moments that can be created by a community pharmacy intervention. | Multi-component lifestyle intervention including advice on health, strength, fitness assessment, immediate feedback, lifestyle prescription with telephone support and reassessment 12-weeks later. Measured weight, BMI, waist circumference, hip: waist ratio, blood glucose, cholesterol, blood pressure, QRISK2  Service duration: 12 weeks | Two themes emerged that explained how men experienced the community pharmacy intervention as a teachable moment and the social process of making lifestyle changes.  The findings confirmed that a teachable moment was not merely a serendipitous occurrence but can be intentionally created through an intervention that provided immediate symptomatic feedback, highlighting the need for change. While other studies have recognised the importance of teachable moments in cancer survivorship, the study offered new insights into the underlying mechanisms and identified how healthcare professionals can shape encounters to promote patient readiness to consider lifestyle changes.  The social context was also a significant factor beyond the teachable moment, influenced by social interactional components.  The results emphasised the importance of moving beyond individually focused interventions when addressing dietary patterns and physical activity habits. Carefully targeted interventions that involved families affected by cancer may yield broader health benefits and have implications for defining the target audience for teachable moments. |
| Price et al (2022) ^22^ | Longitudinal mixed-methods evaluation including secondary data analysis, interviews with service users, and interviews with service providers (at 3-month and 12-month follow-up), with an additional text message survey of service users at 12-month follow-up.  In person  pharmacy-supported  smoking cessation scheme | Report on the second pilot scheme of a pharmacy-supported smoking cessation intervention using e-cigarettes aimed at smokers in manual and routine occupations. The evaluation included a 12 month follow up. | N= 871  Service users’ who smoke. Data collected at baseline, 2 weeks and 4 weeks.  Majority females (56%); mostly aged between 45-54 years old (24%); mostly White ethnicity (87%)  Interviews conducted with 26 of the participants at three months post-intervention | Smoking abstinence and/or smoking reduction across 12 months.  Impact and perceived value of the project on current smokers’ behaviour  Experience of delivering the project from a pharmacy perspective, including facilitators and barriers to encouraging participants to stop smoking using an e-cigarette.  Understand the experience of engaging in the project, including facilitators and barriers to quitting  smoking using an e-cigarette. | Pharmacy-led smoking cessation scheme including e-cigarettes, a charger, and  Fluids as well as practical advice and support on smoking cessation and e-cigarette use available throughout the programme.  Service duration: 4 weeks | Findings demonstrated significant reductions in tobacco use among service users. A total of 871 people participated in the scheme, with 18% (confirmed by CO testing) to 26% (confirmed and unconfirmed) of the sample no longer using tobacco at the 3-month follow-up. Among those who remained in contact with the scheme, 55% had quit tobacco at the 12-month follow-up, representing 5% of the full sample.  Up to one-quarter of participants who received a free e-cigarette and fluid, along with advice on how to use it, stopped smoking tobacco within four weeks. Additionally, 61% of those who continued to smoke had reduced their tobacco intake by five cigarettes per day.  Qualitative findings revealed that service users were highly satisfied with the intervention, reporting health and financial benefits, as well as some positive aspects of e-cigarette use. However, key barriers, including the use of smoking as a coping mechanism for poor mental health, the initial adjustment to using e-cigarettes, and concerns over the potential harms of e-cigarettes, were reported.  Pharmacists identified key facilitators for the programme's success, including widespread and effective advertising, the community pharmacy setting, and the provision of e-cigarettes. Key barriers included a lack of commitment from service users, the short timeframe of the study, and a lack of primary care referrals.  Overall, findings indicated that a community pharmacy-based intervention providing free e-cigarettes and advice can effectively reduce tobacco use, with significant quit rates observed at both 3-month and 12-month follow-ups. The intervention was well-received by service users, who reported various health and financial benefits. Addressing identified barriers and focusing on targeting identified facilitators can help sustain and expand the impact of community pharmacy-based smoking cessation services. |
| Quirk et al (2016) ^33^ | Qualitative process study  In person  Community pharmacist brief alcohol intervention delivery. | Explore participants’ engagement with the trial,  so as to identify whether research participation effects may explain why the brief intervention was not  found to be effective. | N= 24  Service users who consumed three or more drinks monthly or more regularly completed the AUDIT and their eligibility to participate depended on AUDIT scores that indicated problem drinking.  Mean age 38 years old; mostly male (54%); mostly White (67%) | Discussion on possible impacts of trial participation. | The purpose of the brief intervention was to encourage participants to think further about their drinking and whether they should reduce it, and if they are ready to do so, to discuss how. Participants allocated to the intervention condition were offered a discussion of approximately 10-min duration. This intervention contains a number of structured components. The conversation began by building rapport through asking questions about participants’ experience of answering the AUDIT screening questions. Participants were then encouraged to talk about how drinking fitted in with their lives, explore any ambivalence and elicit their evaluation of their drinking, including any problems associated with it. The conversation closed by either the participant or the pharmacist providing a summary of the conversation.  Service duration: 10 minutes | Participation in the trial prompted reflection on alcohol consumption, with AUDIT screening serving as a significant motivator for enrolment, particularly for participants seeking insight into their drinking patterns. This aligned with the AUDIT’s original purpose as a tool to facilitate behavioural contemplation. A quarter of interviewees reported joining the study to assess their personal drinking risk profiles.  A key theme emphasised the critical role of trust and pre-existing rapport between participants and pharmacists, highlighting the importance of the doctor-patient relationship. Approximately half of the intervention group noted no change in attitudes or behaviours, as they did not initially perceive a drinking problem. However, others indicated that the intervention had “got them thinking” about their alcohol use. While pharmacist-led discussions and trial procedures encouraged reflection, there was no discernible difference in the depth of this reflection between intervention and control groups, suggesting limited added value from the structured 10-minute consultation.  Participants perceived pharmacists as credible health advisors comparable to general practitioners, highlighting potential for pharmacist-led interventions in promoting behavioural health changes.  In conclusion, the study highlighted the role of structured interventions in prompting reflection on alcohol use, particularly through AUDIT screening. While trust in pharmacists’ mirrors that in general practitioners, the intervention’s impact was inconsistent, with half of participants remaining unmoved. These findings showed the need for tailored, relationship-based approaches to enhance engagement and effectiveness in pharmacist-delivered alcohol counselling. Despite limitations, the study affirmed the community pharmacists’ potential as trusted agents in health promotion. Interventions should be personalised and integrated with existing patient-pharmacist relationships. |
| Rivas et al (2017) ^23^ | Focused ethnography – mixed methods approach  In person  Communication strategies associated with smoking cessation | To determine communication strategies  associated with smoking cessation in the National Health  Service community pharmacy Stop Smoking programme. | N= 9 stop smoking advisers  N= 16 pairs of smokers.  Median age 41 years old (range 18-67); 34% female; 57% White  Smokers who either quit or did not quit at 4 weeks. Must be part of the NHS Stop Smoking programme. | Content analysis  Stop smoking advisors selectively audio recorded: The first consultation, the consultation at 2 weeks post proposed date, The consultation at 4 weeks. | Community pharmacy NHS Stop Smoking programme  Service duration: Unclear | Three overarching themes influencing cessation outcomes identified**:** (1) negotiating the smoker-adviser relationship, (2) roles of advisers and smokers in quit attempts, and (3) misalignment in perceptions of smoking motivations and relapse triggers. Advisers who used core communication strategies (e.g., personalised advice, empathetic listening) in 50% or more of consultations had higher success rates, with these strategies occurring more frequently in cases where smokers quit at 4 weeks.  The study concluded that effective smoking cessation required tailored communication that aligned with smokers’ individual motivations and addressed relational dynamics. It emphasised the need for training advisers in patient-centered approaches to enhance strategy application and reduce misalignment. The findings highlight the importance of structured communication frameworks in community pharmacy settings to improve quit rates and support long-term behavioural change. |
| Saramunee et al (2016) ^44^ | Cross-sectional survey.  In person  Services related to CVD risk factors including smoking cessation, drinking, losing weight, heart health advice, blood pressure, blood sugar and cholesterol checks. | to identify the pharmacy characteristics perceived as desirable by different sectors of the general public and the promotional methods for pharmacy public health services they consider as likely to influence them. | N= 2661 surveys questionnaires (2047 face-to-face, 301 telephone, 313 paper-based)  Female= 57%, majority aged 65 years and over; 85% White ethnicity  University level education= 40% | Services used including smoking cessation, drinking, losing weight, heart health advice, blood pressure, blood sugar and cholesterol checks which form the basis of a national CVD prevention programme in England, the NHS Health Check.  Characteristics of pharmacies and staff.  Frequency of pharmacy use and demographic questions such as deprivation status. | Identifying the type of services on offer by pharmacies in England.  Service duration: Not reported | The strongest preferences for pharmacy characteristics focused on location, accessibility, and familiarity. A significant majority of respondents (84.7%) prioritised pharmacies near their homes, while 67.8% preferred pharmacies close to their general practitioner’s office. Additionally, 63.6% valued pharmacies open on Saturdays, and among the 1,300 respondents in full- or part-time work, nearly half (45.5%) favoured pharmacies near their workplace. Over half of respondents (56.2%) expressed a preference for using the same pharmacy consistently, and just over a third valued knowing the pharmacist personally (35.4%) or having staff who recognised them (34.4%). Trust in pharmacists and staff was notably high, with 89.6% trusting pharmacists and 84.9% trusting staff to maintain confidentiality.  Demographic variations revealed distinct preferences among different groups. Respondents over 65, retirees, frequent pharmacy users, and those with primary or secondary education were more likely to prefer pharmacies owned by the pharmacist on-site and to avoid supermarket pharmacies. This preference was also shared by individuals with higher socioeconomic status (SES) and those living in areas of low deprivation. Working individuals and non-white respondents were more likely to prioritise pharmacies with extended hours, such as those open on weekends or evenings. Retirees and women were more inclined to request private consultation spaces, while trust in confidentiality was highest among retirees, those over 65, individuals with lower educational attainment, and white respondents.  When it came to promotional strategies, personal recommendations were the most influential. Nearly 90% (89.4%) of respondents were likely or possibly likely to be influenced by recommendations from healthcare professionals, and 86.5% valued recommendations from friends and family. Local visibility also played a key role, with posters in GP offices (76.7%) and pharmacies (71.0%) being highly effective, followed by healthcare websites (65.0%). In contrast, mass-media methods such as newspapers and radio appealed to fewer than 25% of respondents.  For pharmacy public health services to be more widely adopted, pharmacists, pharmacy companies, and service commissioners must align promotional methods with the preferences of their target audiences. The authors mentioned that this included prioritising word-of-mouth recommendations and local visibility over mass-media campaigns. Additionally, addressing demographic-specific needs – such as extended hours for working professionals and private consultation spaces for retirees – can further enhance service uptake and satisfaction.  In conclusion, the study highlighted the importance of understanding public attitudes toward community pharmacy attributes and preferences for promoting public health services. The findings revealed that accessibility, professional competence, and trust were key factors influencing public perception. Additionally, the study emphasises the need for tailored communication strategies to effectively promote pharmacy-based health services. These insights can guide policymakers and pharmacy practitioners in enhancing service delivery and public engagement. Overall, the research demonstrated the potential of community pharmacies as vital hubs for delivering public health initiatives. |
| Saramunee et al (2015) ^24^ | Mixed methods study involving a cross-sectional survey and focus group of survey respondents.  In person  Cardiovascular public health services | Explore in a wide, cross-sectional survey, the  experience of and willingness to use pharmacy public health services among the general  public in England. | N= 908  Pharmacy service users; fewer than half (42%) were working and most were classed as of high socioeconomic status (62.5%).  Mostly aged between 35 to 64 years old (54%)  The proportion of smokers (18.2%). The proportion of respondents who were increasing risk drinkers (39.0%), had a healthy diet (32.3%), were physically active (53.0%) and overweight.  N= 5 (focus group)  Male= 3; four held managerial/professional occupations and one had an unskilled/manual occupation. | Experience of, and willingness to use, the seven pharmacy public health services. Respondents’ experiences of each service were measured using bivariate (yes/no) options.  Willingness to use the seven services in the future was measured categorically (yes, maybe, or no), then responses were dichotomised to positive (answered ‘yes or maybe’) or negative view (answered ‘no’). | Perspectives on public health services offered by pharmacies. Services relating to CVD prevention including four services for health checks (smoking cessation, sensible drinking, losing weight, heart health) and three health checks (blood pressure, blood sugar and cholesterol monitoring).  Service duration: Not reported | Findings showed that only a small proportion of respondents (2.1–12.7%) had previously used any of the seven pharmacy-based public health services. While approximately 40% expressed willingness to use health check services, fewer participants (9.3–26.3%) were open to participate in advisory services. Willingness to use services in general was higher among females, frequent pharmacy visitors, and those in good health (*p*<0.05). Specific advisory services targeting health issues such as smoking, weight management, and alcohol-related problems were more likely to attract individuals facing those challenges (*p*<0.05). Focus group discussions (FGD) identified barriers to service uptake, including frequent staff turnover, the perception of pharmacists primarily as medication providers, and doubts about their competence in delivering public health services.  Findings also revealed that while many respondents were aware of basic pharmacy services, such as medication dispensing, awareness of broader public health services like smoking cessation, weight management, and health screenings was limited. For instance, only 30% of participants were familiar with smoking cessation programmes offered by pharmacies. This lack of awareness highlighted a significant gap in public knowledge about the full scope of services pharmacies can provide.  Public attitudes toward using pharmacies for public health services were also examined. Respondents expressed high levels of trust in pharmacists’ professional competence, with over 70% indicating they would consider using pharmacies for health advice. However, willingness to engage varied by service type, with greater interest in straightforward services like blood pressure monitoring (65% willing) compared to more complex interventions such as alcohol reduction programmes (45% willing). This suggested that while the public values pharmacists’ expertise, they may need more encouragement to adopt certain services.  Barriers to utilising pharmacy public health services were also identified. Key concerns included privacy issues, perceived lack of time during pharmacy visits, and uncertainty about the cost of services. Additionally, some respondents felt that pharmacies were not the appropriate setting for certain health interventions, preferring traditional healthcare providers like doctors. These findings highlighted the need for pharmacies to address misconceptions, improve service accessibility, and enhance communication about the benefits and confidentiality of their public health offerings.  In conclusion, the study highlighted a clear opportunity for pharmacies to expand their role in public health, provided they address existing barriers and increase public awareness. By utilising the high levels of trust in pharmacists and tailoring services to meet public preferences, pharmacies can become more integral to community health initiatives. Strategic marketing, improved privacy measures, and clearer communication about service availability and affordability were essential steps to optimise public engagement. Overall, the study highlighted the potential for pharmacies to play a pivotal role in addressing public health challenges in the future. |
| Savickas et al (2020) ^45^ | Cross-sectional feasibility study  In person  AF screening | Determine the feasibility of general practice–based clinical pharmacists screening the over-65s for AF, using digital technology and a single-time-point screening strategy combined with another annual healthcare intervention | N= 604  Participants across four GP practices in Kent who undertook a heart rhythm check with a clinical pharmacist.  White British= 96.9%  BMI= 26.1; mean age 73 years old (range 69-78); 43% male | ECG (AliveCor Kardia Mobile Device) (KMD)  Heart rate check  Feedback from participants of AF screening by pharmacist. | ECG, Heart rate check, AF screening services  ECG assessed and interpreted by clinical pharmacist and explained to participant. All ECGs emailed to study cardiologist via NHS.net mail with a provisional diagnosis, and were returned within 72 hours. Patient interventions were organised by their GP  Service duration: 16 days in total including follow up ECG test or heart rate check if required. | Clinical pharmacists, using the Kardia Mobile Device (KMD), accurately identified 24 out of 26 potential Atrial Fibrillation (AF) cases, closely matching the diagnoses confirmed by a cardiologist. Participants diagnosed with AF were more likely to have co-existing or multiple health conditions, including hypertension, renal disease, diabetes, and heart failure. Both newly detected and previously known AF cases were found to have a high stroke risk, making them suitable candidates for oral anticoagulant (OAC) therapy. The study also highlighted that AF screening for individuals over 65, when combined with other healthcare interventions and the use of the KMD, was cost-effective and financially beneficial compared to no screening. Importantly, relying solely on pulse palpation (which measures seven pulse areas) led to a higher rate of false-positive AF diagnoses (7.8%) compared to the KMD (2.6%).  Participants (n=422) provided feedback through a questionnaire, with all respondents rating their overall experience as "good" or "very good." They expressed high satisfaction with the pharmacist-led screening process and the information they received. Nearly all (99%) indicated a willingness to participate in annual AF screening, and the majority (96%) considered routine AF screening to be "very important" or "important." However, a significant proportion (53%) were unaware of AF as a condition prior to the screening.  The study demonstrated that pharmacist-led AF screening in general practice settings was feasible, cost-effective, and well-received by participants. The use of single-lead electrocardiograms (SLECGs) by clinical pharmacists proved to be more sensitive and specific for AF detection compared to pulse palpation alone. Future research should focus on identifying and addressing barriers to implementing national AF screening programs to enhance early detection and treatment. |
| Seston et al (2020) ^25^ | Mixed methods involving a before and after design, interviews.  In person  Greater Manchester Community Pharmacy Care Plan (GMCPCP) service | Investigate the impact of patients participating in the GMCPCP service on a number of clinical and process measures (see outcomes) | N= 382  Service users  Mean age 63 years old | Patient activation (PAM-13)  NHS resource use and clinical outcomes such as blood pressure, high density lipoprotein (HDL) cholesterol ratio and blood glucose  The nature of goals set, and the extent to which patients were able to achieve these.  Patient satisfaction and acceptability of the service  Quality of life, as measured by EQ-5D (EQ-5D-5L and EQ-VAS; EuroQol group  Medicines adherence, (MARS-5) | GMCPCP workshop including a half-day role-play session with medical actors. Health Care Assistants (HCAs) were trained in the use of equipment to measure clinical indicators, how to administer the questionnaires and the entering of data onto the online platform (PharmOutcomes) used for recording service data. Participating patients attended up to four consultations (baseline, 2, 4 and 6 months).  The GMCPCP service was established to provide a tailored community pharmacy care plan service to help adult patients with one or more of four qualifying long-term conditions (hypertension, asthma, diabetes and COPD) achieve their health-related goals and better manage their long-term condition(s).  Service duration: one-day training for pharmacists. | Patients who participated in the services showed significant improvements in several key health metrics, including patient activation levels, quality of life, medication adherence, systolic blood pressure, weight, BMI, and HDL cholesterol ratio. Over half of the participants who remained in the programme improved their Patient Activation Measure (PAM) score, with the most substantial progress seen among those starting at the lowest activation levels (1 and 2). NHS service usage costs also decreased significantly from baseline to follow-up across most categories, except for GP home visits, resulting in modest overall cost savings.  The study indicated that the service is well-received by patients and has the potential to enhance health outcomes. The supportive role of pharmacists was crucial in maintaining patient engagement, and interactions with pharmacists helped patients better understand their conditions. However, the study suggested that follow-up sessions could have further strengthened the programmes impact. At a time when healthcare systems face unprecedented demand and a shortage of GPs, who typically manage long-term conditions, this research highlighted the valuable role community pharmacists can play in improving patient care. The authors pointed out that their involvement not only supported better health outcomes but also alleviated pressure on traditional healthcare providers. |
| Sohanpal et al (2016) ^34^ | Qualitative semi-structured in-depth interviews  In person  NHS community pharmacy stop smoking service | Understand views of pharmacy  advisers about smoker recruitment and retention in the  National Health Service community pharmacy stop  smoking programme. | N= 25 stop smoking advisers  (13 pharmacists and 12 support staff) across 29 community pharmacies in 3 inner east London boroughs.  Mean age= Not reported | Perceptions of smoking cessation advisers  Using Theoretical domains framework and COM-B behaviour change model as frameworks for analysis. | NHS community pharmacy stop smoking service includes content on the delivery of behavioural support together with pharmaceutical treatments comprising nicotine replacement therapy (NRT), for example, patches, prescribed medication, for example, varenicline (Champix) or a combination of NRT and prescribed medication to help a smoker quit smoking.  Week 1 – introduction and set planned quit date; stop smoking advisor explains process and gives information about available medication, discussion of what is most suitable, CO reading  Weeks 2-4 – brief meetings to check progress – CO monitoring at week 4  Weeks 5-6 – longer meetings to discuss motivations and relapse prevention  Weeks 6-12 – programme available but no obligated FU  Service duration: 12 weeks | The findings revealed that advisers viewed recruitment as a significant challenge, often due to low public awareness of the service and misconceptions about its effectiveness. Many advisers highlighted the need for better marketing and outreach to attract smokers, particularly those from hard-to-reach groups, such as younger individuals and those from deprived backgrounds. They also noted that competing services, such as those offered by GP practices, further complicated recruitment efforts. Advisers emphasised the importance of building trust and rapport with potential clients to encourage initial engagement.  Retention of clients in the stop smoking service was another key focus of the study. Advisers identified several barriers to retention, including lack of motivation, withdrawal symptoms, and external stressors such as work or family pressures. They also noted that clients often underestimated the difficulty of quitting and were unprepared for the challenges they would face. Advisers stressed the importance of providing personalised support and maintaining regular contact with clients to keep them motivated. Flexible appointment scheduling and follow-up sessions were seen as critical to improving retention rates.  The study also highlighted the role of training and resources in supporting advisers. Many participants felt that additional training in behaviour change techniques, and motivational interviewing would enhance their ability to support clients effectively. Advisers also called for better access to resources, such as informational materials and quit-smoking aids, to help clients stay on track. Some noted that limited time and high workloads constrained their ability to provide individualised care, suggesting a need for more staffing or dedicated time for smoking cessation services.  Advisers also discussed the importance of collaboration with other healthcare providers to improve service delivery. They suggested that closer integration with GP practices, hospitals, and community organisations could help identify and refer more smokers to the service. Additionally, they emphasised the value of peer support networks, where former smokers could share their experiences and encourage current clients. Advisers believed that a more coordinated approach across the healthcare system would strengthen the service’s reach and impact.  In conclusion, the Sohanpal (2016) study highlighted the challenges and opportunities in recruiting and retaining clients in the NHS community pharmacy stop smoking service. Advisers identified low awareness, misconceptions, and competing services as barriers to recruitment, while motivation, withdrawal symptoms, and external stressors hindered retention. The findings suggested that improved marketing, personalised support, and better training and resources could enhance the service’s effectiveness. Collaboration with other healthcare providers and peer support networks were also seen as key to expanding the service’s reach. Overall, the study highlighted the potential for community pharmacies to play a vital role in smoking cessation, provided the identified challenges are addressed. |
| Steed et al (2017) ^26^ | Mixed methods study  In person  Two 150min, skills-based training sessions  focused on communication and behaviour change skills  with between session practice | Develop a complex intervention for  community pharmacy staff to promote uptake of smoking  cessation services and to increase quit rates. | N= 12 stop smoking advisers  Pharmacy workers across 8 community pharmacies in three inner east London boroughs  Mean age= Not reported | Outcomes from five areas of work including interviews and conversation analysis of audio recorded consultations, literature synthesis, realist review of smoking cessation intervention in community pharmacies, theoretical modelling, and piloting in pharmacies. | Two-session face-to-face training programme targeting communication and behaviour change skills with homework tasks, social media support and a paper-based prompt tool.  Pharmacy staff  Service duration: Two 150 minutes skill-based training | Interviews with pharmacy workers after the pilot study suggested that the face-to-face training was well received by those attending and that such training might improve self-efficacy in delivering smoking cessation interventions. However, only 6 of 12 participants attended both training sessions and 4 individuals did not attend any sessions, suggesting a need for changing recruitment processes.  There are possible operational barriers that need to be considered. For example, healthcare professionals may not find it acceptable to blur the boundaries between professional and social life. For this form of intervention to be successful, it may be necessary to give greater consideration to the motives and barriers towards using social media. Other barriers included time and getting locum cover  Findings also showed that explicit attention needs to be given to the implementation of the intervention and integration with routine practice.  Results also highlighted the need for flexibility in how the training sessions are delivered, for example, at a central location or in individual pharmacies. The revised intervention training will be offered at different times of day and on different days of the week and those who are unable to attend will be offered on-site training.  The study also explored the impact of the intervention on smoking cessation outcomes. Participants who received the intervention showed positive changes in their smoking behaviours, including reduced cigarette consumption and increased quit attempts. Pharmacy workers played a crucial role in providing personalised support, which was valued by participants. The intervention’s emphasis on building rapport and tailoring advice to individual needs was seen as a key factor in its success. Despite these positive outcomes, the study acknowledged that long-term quit rates were not significantly higher compared to standard care, suggesting room for further refinement of the intervention.  Feedback from pharmacy workers revealed that the training program improved their knowledge and skills in delivering smoking cessation support. They appreciated the structured approach, and the use of behaviour change techniques, which helped them engage more effectively with clients. However, some workers expressed concerns about the additional time required to deliver the intervention, particularly in busy pharmacy environments. The study highlighted the importance of ongoing training and support to sustain the intervention’s implementation and ensure consistency in service delivery.  Participants in the study reported high levels of satisfaction with the pharmacy-led smoking cessation service. They valued the accessibility and convenience of community pharmacies as a setting for receiving support. Many participants appreciated the non-judgmental and supportive approach of pharmacy workers, which helped them feel more motivated to quit. The study also found that participants who had previously struggled to quit smoking were more likely to engage with the intervention, suggesting that it effectively reached a high-need population. However, some participants noted that follow-up support could be improved to help them maintain long-term abstinence.  In conclusion, the Steed et al. (2017) study demonstrated that a theory-based smoking cessation intervention delivered by community pharmacy workers was feasible and well-received by both staff and participants. The intervention showed promise in supporting short-term behaviour change and increasing quit attempts, though its impact on long-term quit rates was limited. The findings highlighted the importance of adequate training, resources, and follow-up support to enhance the intervention’s effectiveness. The authors mentioned that further research was needed to optimise the intervention and address barriers to implementation. |
| Stewart et al (2020) ^51^ | Multi-site cluster randomised controlled pilot trial  In person  Medicines and Alcohol Consultation (MAC) | Investigate all study procedures to inform progression to the definitive trial. Specific objectives addressed in this paper were to investigate the following trial procedures: the feasibility of the recruitment strategy for CP and patient participants; attrition from the trial during recruitment and at 2-month follow-up; delivery of the MAC practice development programme for intervention CPs; measurement of the proposed trial primary outcomes, including data quality issues associated with alcohol consumption recall bias. | N= 10 community pharmacies  One defined geographic area (1.5 hr of travel time from York, UK)  N= 260  Adult patients who consumed alcohol for at least twice per week.  Mean age= 67 years old; Female= 45% | Recruitment of CPs  Delivery of the MAC practice development programme  The proportion of patients approached for the trial who accepted the initial invitation.  Proportion of patients accepting the invitation  Proportion of eligible patients who consented  Proportion of recruited participants who provided follow-up data (interview or postal questionnaire)  Candidate primary outcome measures for the main trial were total weekly UK units (8 g of ethanol per unit) of alcohol consumption in the 7 days prior to follow-up.  Confidence in medications management measured using the PROMIS Self-Efficacy for Managing Medications and Treatment scale (6 item version) [21].  Candidate secondary clinical outcomes were: quality of life measured by the EQ. 5D-5L.  Adherence measured by ProMAS; anxiety (GAD-7 and depression (PHQ-8) | MAC programme comprised of the following components:  Two practice development training days.  A four-page paper-based MAC guide summarising the structure of MAC and core content within consultations.  A range of learning support resources including case studies, information about interplay between alcohol and specific medications, and practice development exercises.  Individually tailored weekly practice development support site visits or telephone calls by the MAC support team.  Invitation to engage in peer support (e.g., group discussions on WhatsApp)  Service duration: Unclear | Findings indicated that the MAC intervention was feasible to implement, with pharmacists reporting increased confidence in discussing alcohol use. However, some pharmacists noted challenges, such as time constraints and concerns about patient receptiveness, which highlighted the need for additional training and support.  The study found no significant difference in alcohol consumption reduction between the intervention and control groups at the six-month follow-up. This suggested that while the MAC intervention was effective in identifying risky drinking, additional strategies may be needed to support sustained behaviour change. Pharmacists emphasised the importance of ongoing training and resources to enhance their ability to deliver the intervention effectively. The study also highlighted the potential for community pharmacies to play a broader role in public health by addressing alcohol use alongside medication management.  In conclusion, the study demonstrated that the MAC intervention is a feasible and acceptable approach to integrating alcohol discussions into community pharmacy medicine reviews. The intervention significantly improved the identification of risky alcohol consumption, though it did not lead to measurable reductions in alcohol use over time. The findings highlighted the potential for community pharmacies to contribute to alcohol harm reduction efforts, particularly when pharmacists are equipped with the necessary skills and resources. Community pharmacies, with their accessibility and trusted relationships with patients, are well-positioned to play a key role in addressing alcohol-related health issues. |
| Sturrock et al (2017) ^27^ | Mixed methods study involving a patient survey questionnaire and semi-structured interviews with pharmacy staff.  In person  Pilot oral health promotion intervention | The objectives of the pilot were as follows:  (1) explore whether community pharmacies are a suitable venue for a brief oral health intervention  (2) explore any barriers or facilitators for this brief intervention  (3) explore the training needs required for the brief intervention. | N= 1069  Mean age= 52 years old  Female= 65%  N= 5 interviews  From four pharmacies: One pharmacist and four were pharmacy assistants. | Views of the service and to identify emerging concepts to be explored further during the interview process.  Demographic information  Details of the patient’s last dental appointment  Three questions regarding the intervention. | Demonstration of how teeth should be cleaned through the use of a phantom demonstrator head and large toothbrush. Supporting information and advice to back up the intervention from the Oral Health Foundation and details of local dental providers were offered to each patient. A  bag containing 1,500 ppm fluoride toothpaste,  a soft to medium texture small head toothbrush and an information leaflet supporting the advice given during the intervention were given to each patient. Interventions lasted  approximately 5-10 minutes.  Trained pharmacy staff including pharmacists, pharmacy  technicians and pharmacy assistants  Service duration: Not reported | Patients reported positive perceptions of the oral health promotion intervention, with 71.65% noting a significant improvement in their knowledge of oral health practices, describing it as "much better" post-intervention. Additionally, a substantial proportion (68.35%) expressed increased motivation to adopt healthier oral hygiene routines. The intervention successfully identified a subgroup of patients who do not engage in routine dental care: 16.37% (n=175) had not visited a dentist in the past two years, with those aged 65 years or older being the most underrepresented in dental attendance. This demonstrated the pharmacies' potential to reach populations disengaged from traditional dental services.  Qualitative analysis revealed three key themes: intervention feedback, knowledge gaps, and service development needs. Pharmacy staff echoed patients' positive experiences, reporting smooth recruitment and high acceptability of the service. Training programmes were deemed effective in enhancing staff confidence and competence, particularly in recommending appropriate oral health products. However, interviews highlighted that patients often conflated oral health with dental care (e.g., focusing solely on teeth), especially among denture wearers, suggesting a need for broader education on systemic oral health links.  Participants proposed strategies to enhance the intervention’s reach, including collaboration with dental teams to improve interprofessional communication and expanding access to vulnerable groups such as children or housebound individuals. These adaptations, while promising, would likely require additional staff training to address new complexities. Overall, the findings confirmed that oral health promotion in community pharmacies aligned with the goals of Healthy Living Pharmacies (HLPs), offering a viable and accepted approach to improving oral health outcomes at the population level. |
| Weidmann et al (2015) ^35^ | Qualitative interviews  In person  Community pharmacy weight management | Explore the beliefs and experiences of pharmacists and Medicines Counter Assistants (MCAs) in the North-East of Scotland on community pharmacy weight management. | N= 20 MCAs; 31 pharmacists  Registered community pharmacies across North-East of Scotland  Overall characteristics of participants: majority aged 29 years old or below (38%); mostly female (79%); mostly less than 5 years in current role (47%) | Pharmacists’ and MCAs’ beliefs and experiences with delivering weight  management services in community pharmacy. | Community pharmacy weight management including measurement of weight, waist, calculation of BMI, body fat, advice on healthy eating and supply of weight loss products.  Delivered by CPs or Medicines Counter Assistant (MCA)  Service duration: Not reported | Pharmacists and MCAs identified clear benefits of the weight management service, including improved patient-pharmacist interactions and motivation among staff. They reported that the service fostered a sense of purpose and strengthened relationships with customers. Additionally, the structured approach to providing advice and products was perceived as valuable for addressing obesity and related health risks.  However, challenges such as excessive administrative burden and information overload emerged. Qualitative data revealed that too much paperwork and detailed guidelines were overwhelming for staff, particularly those with limited experience. MCAs and pharmacists also highlighted difficulties in balancing weight management duties with routine pharmacy tasks, leading to concerns about time constraints and resource allocation.  Participants emphasised that collaboration with colleagues and the opportunity to engage with patients on health issues were the most helpful aspects of the intervention. Conversely, the least beneficial components were overly complex documentation and the provision of excessive information, which detracted from practical service delivery.  This study highlighted the potential of community pharmacies to contribute to weight management initiatives through staff-patient interactions and structured advice. While pharmacists and MCAs acknowledged the service’s benefits in motivating patients and fostering professional engagement, systemic barriers such as administrative burdens and lack of streamlined resources hindered its effectiveness. Addressing these challenges through targeted training, simplifying documentation, and optimising workflow integration could enhance the sustainability and impact of pharmacy-led weight management programmes. The findings advocated for a balance between service innovation and operational feasibility to maximise public health benefits. |
| Whittaker (2019) ^61^ | Comparison study design (two matched primary care practices)  In person  NHS Health Checks | Compare the uptake of NHS Health Checks for two matched primary care practice populations, one being offered NHS Health Checks within the primary care practice and the other being invited to attend a community pharmacy.  Majority of service users were aged 40 to 55 years old (80%) | N= 264 patients attending primary care practice  N= 234 attending a community pharmacy  Female= 82.92%  1.9 Mixed, 2.1 to 2.9% Asian, and 1.1 to 1.6% Black | Aggregated monitoring (including uptake) and invoice returns received from the practice and community pharmacies setting.  Patient data collected in community pharmacies was transferred to the general practice patient record via secure electronic transfer. | Complete NHS Health Check includes:  Age; gender; ethnicity; smoking status; family history of coronary heart disease; blood pressure, systolic and diastolic; BMI; General practice PA questionnaire; alcohol use score; cholesterol; QRISK2 score; dementia awareness and validated diabetes risk assessment score.  Service duration: Not reported | A total of 1,265 eligible patients received their first invitation to book an NHS health check from the primary care practice where the in-practice service was offered. Among these, 264 individuals (21% uptake) attended a check at the primary care practice between April 1, 2015, and March 31, 2016 , while 234 patients (23% uptake) chose to participate in community pharmacy-based checks during the same period.  Of the 264 primary care attendees, 13 (5%) were identified as having a 10-year risk of ≥20% for developing symptomatic cardiovascular disease (CVD) , compared to 6 individuals (3%) among the 234 community pharmacy participants . No statistically significant difference was observed in the proportion of high-risk individuals between the two settings, indicating that community pharmacies can effectively serve as an alternative to primary care practices for delivering NHS Health Checks.  Findings also showed that 68% of survey respondents (n=66) reported making lifestyle changes, such as increasing exercise (47% ) or improving diet (45% ). However, written advice was provided to only 55% of participants, while 86% received verbal guidance. This disparity suggested a reliance on oral communication over standardised written resources, potentially limiting the depth of patient understanding.  The study found no significant differences in total AUDIT scores (Alcohol Use Disorders Identification Test) between intervention and control groups, nor in changes over time. While smoking cessation advice was frequently offered, only 5% of participants reported reducing alcohol intake or quitting smoking, indicating limited impact on these behaviours.  Participants valued the convenience and accessibility of pharmacy-based health checks, with 79% of survey respondents appreciating the non-judgmental environment. However, privacy concerns were raised by some, particularly regarding discussions of sensitive topics like alcohol use.  Offering NHS Health Checks in a community pharmacy was a feasible alternative to performing checks in general practice. Offering checks opportunistically as well as by appointment would maximise uptake and may reach more patients at greater risk of CVD. |
| Wright et al (2019) ^63^ | Economic modelling analysis  In person  Diabetes screening | Determine the cost per patient with type 2 diabetes appropriately referred to their GP through community pharmacies using the current UK model within different geographical settings and assuming different rates of referral to GPs | N= 172  Service users from six pharmacies in Leicester and five from Surrey.  Females= 57.2% (Leicester); 67.1% (Surrey)  Age= 54.1% (Leicester), and 66.5% (Surrey) were over 50 years old | Pharmacy location  Age group  Gender and ethnicity of patient  Risk assessment score derived from the customer report form.  HbA1c result  Average number of individuals seen within each pharmacy was calculated to inform service costs over a one-year period. | Pragmatic community pharmacy-led service  Service duration: Not reported | The study found that the cost per diabetes screening test in community pharmacy settings was marginally higher than in medical practice-based studies. However, the proportion of patients identified as at high risk of diabetes in both Leicester (54.1% over 50 years old) and Surrey (66.5% over 50 years old) pharmacies was comparable to results from a Leicester medical practice-based population screen. This suggests that community pharmacy-based diabetes screening services can achieve outcomes similar to those of traditional medical practices.  The cost per appropriately referred individual to general practice for confirmatory testing ranged from £7,638 to £18,828, varying by geographic location and patient motivation to follow up with their GP. Additionally, the cost per confirmed diabetes diagnosis referral was 33% lower in Leicester than in Surrey, attributed to the higher prevalence of undiagnosed diabetes in Leicester. This highlighted the cost-effectiveness advantage of targeting pharmacy-based screening programmes in areas with greater prevalence of diabetes.  The findings emphasised that community pharmacy-led diabetes risk assessments and screenings were comparable in cost and diagnostic accuracy to medical practice-based approaches. To optimise cost-effectiveness, the study recommended prioritising service implementation in regions with higher diabetes prevalence and ensuring structured follow-up mechanisms to encourage at-risk patients to self-refer to medical practices for confirmatory testing.  All in all, community pharmacies offer a feasible and cost-effective alternative to medical practices for diabetes screening, particularly when deployed in areas with high prevalence of diabetes. Strategic placement of services and interventions to improve patient follow-up can enhance their public health impact while maintaining economic efficiency. |
| Wright et al (2015) ^66^ | Before and after study and economic evaluation  In person  Community pharmacy-based chronic obstructive pulmonary disease support service. | Aim is to describe the effectiveness of a community pharmacy–based COPD service delivered from a wide variety of community pharmacies and to  provide an initial indication as to the cost-effectiveness of the  intervention. | **N**= 137 service users  Service users from 34 pharmacies belonging to the four companies in the Wirral in the North West of England.  Male= 51%  Smokers= 38.2%  Mean age= Not reported | Demographics including age, gender, height, weight, body mass index and smoking status.  Activities undertaken within the initial consultation were recorded.  At baseline:  EQ-5D (T) (quality of life)  Morisky assessment of adherence  Participants were also asked at baseline and at every subsequent visit to report the following items of resource use:  Routine GP visits for COPD  Exacerbations treated by GP  Hospital admissions for severe COPD exacerbations (and associated number of days per admission, if applicable)  Other hospital admissions (and associated number of days per admission, if applicable)  A&E visits  Rescue packs obtained  Days off sick. | The service involved supporting patients to stop smoking (if appropriate)  Also involved helping patients to recognise symptoms of exacerbations and enabling them to respond, e.g., through rescue packs. The service aimed to improve medicines adherence, through the provision of advice on how to use inhalers and ensuring that patients were using the most appropriate inhaler device.  General lifestyle advice was also provided where necessary.  Referral letter to the GP to obtain a COPD rescue pack. Where recommendation was identified, these were also communicated to the patient’s GP. An action plan was agreed with each patient and monitored in follow-up patient visits that were arranged at the patient’s convenience.  Service duration: Not reported | Key outcomes demonstrated statistically significant improvements in quality of life, measured by the EQ-5D questionnaire, with a mean change of +0.029 (95% CI 0.005 to 0.052). Additionally, COPD Assessment Test (CAT) scores decreased significantly, reflecting better disease control.  Secondary outcomes revealed an increase in the use of rescue packs (e.g., inhalers) and a reduction in smoking rates among participants. However, the study lacked a control group, making it challenging to isolate the intervention’s impact. 51% of participants were male, and the service’s implementation highlighted challenges such as inconsistent attendance and unreported service duration, which may have influenced results.  The economic evaluation indicated cost-effectiveness, though specifics were not detailed. Limitations included potential selection bias, as participants were self-referred or identified by pharmacists, possibly skewing the sample toward more motivated individuals. The absence of long-term follow-up data also hindered assessments of sustained outcomes.  In conclusion, the study demonstrated that community pharmacy-based COPD support services can improve quality of life and disease management in COPD patients, with 51% male participants and measurable reductions in CAT scores. While the intervention showed promise in promoting adherence and smoking cessation, its lack of a control group and unclear service duration limit definitive conclusions. Further research with longitudinal designs and cost analyses is needed to confirm scalability and long-term benefits. |
