# Supplementary material for: Integrating cardiovascular healthcare screening into a community pharmacy vaccination service: a scoping review to identify opportunities for patient engagement and service expansion
Source: BMJ Open. 2026 Mar 23;16(3):e108381. doi: 10.1136/bmjopen-2025-108381 (PMC13034389; doi:10.1136/bmjopen-2025-108381)
Supplement: online supplemental table 2 [file bmjopen-16-3-s004.docx]

| **Table 2. What are service users’ experiences of community pharmacist-led services?** | |
| --- | --- |
| **First Author,**  **Year** | **Key themes** |
| Atkin (2020) ^28^ | Key themes identified included:  Clarity on purpose: Many patients were unclear about the purpose of Medication Use Reviews (MURs), viewing them as informal "chats" rather than structured health consultations, and did not remember the advice given.  Preference for GPs: While some appreciated pharmacists' accessibility and time, others preferred discussing healthy lifestyle concerns with GPs, perceiving them as more authoritative or better qualified, particularly for complex issues.  Impersonal interactions: Some patients found pharmacy services transactional, especially when MURs were done quickly, reinforcing a perception of pharmacies as commercial rather than healthcare spaces.  Mixed comfort levels: A minority felt more at ease with pharmacists, reporting positively about their approachability and time, though others hesitated to disclose sensitive issues (e.g., alcohol use) due to fear of judgment or lack of follow-up. The commercial environment of pharmacies, with its emphasis on targets and efficiency, took away from the focus on providing meaningful behavioural interventions, and produced an environment whereby patients often felt they were merely "answering questions" rather than collaborating in care.  Limited behavioural support: Healthy living advice during MURs was often superficial, with pharmacists prioritising medication adherence over in-depth discussions about lifestyle changes.  Fragmented care: Patients with multifaceted needs (e.g., mental health, weight management) received generic advice via leaflets or referrals to GPs, and did not receive in depth support within the pharmacy setting. |
| Brown (2014) (b) ^15^ | The intervention was generally well-received, with service users finding it acceptable to discuss alcohol consumption with pharmacists and not perceiving it as embarrassing. Identified themes included the acceptability of AUDIT screening as a tool for prompting reflection on alcohol use, with participants mentioning its utility in objectively assessing drinking behaviours and fostering open discussions with pharmacists. Privacy concerns also emerged as a significant barrier, with users expressing discomfort discussing sensitive topics like alcohol consumption in pharmacy settings.  Other key themes identified included:  Receptiveness to advice: While many appreciated the advice given and found it useful, a significant portion felt they did not need guidance on drinking.  Appropriateness of pharmacists: The majority viewed pharmacists as suitable professionals to conduct alcohol screening and provide brief advice.  Varied engagement: Some clients engaged meaningfully, particularly when tools like the "drink wheel" helped visualize alcohol units, while others declined participation due to perceived irrelevance or sensitivity. |
| Corlett (2015) ^16^ | Key themes identified included:  Convenience & accessibility: Users valued the speed and ease of accessing on-site tests without appointments.  Expectations vs reality: Most found the service met or exceeded their expectations, with some surprised by the depth of information provided, though a minority felt advice was too basic.  Process & communication: While many appreciated clear explanations and educational tools (e.g., interactive risk calculators), others noted interruptions or insufficient detail about results.  Understanding results: Participants generally grasped their health risks but desired clearer explanations of specific metrics (e.g., blood pressure scores).  Behavioural impact: Few reported major lifestyle changes, though some reduced alcohol, improved diets, or increased exercise. Barriers like time constraints and location limited sustained action.  Areas for improvement: Interruptions during consultations and requests for more personalised, in-depth discussions were reported. |
| Dhital (2022) ^36^ | Key themes identified included:  Access barriers & unmet needs: Participants reported inconsistent access to relapse prevention medication, with GPs often unwilling to prescribe and clinics providing inadequate follow-up support. Many turned to peer groups (e.g., AA) as alternatives.  Knowledge gaps about Acamprosate: While some credited acamprosate for reducing cravings and prolonging abstinence, others doubted its efficacy. Widespread confusion persisted about side effects, drug interactions, and dosing schedules, exacerbated by unclear guidance.  Burden of medication adherence: The regimen (6 tablets/day) was frequently described as disruptive and stigmatising, leading to resentment and missed doses, particularly for those managing multiple medications.  Support preferences: Participants overwhelmingly favoured pharmacist-led telephone support for medication adherence, valuing structured, empathetic check ins. However, concerns arose about pharmacists’ specialist knowledge and the impersonal nature of phone consultations.  Rejection of contingency management (CM): Cash incentives for treatment engagement were widely criticised as unethical, potentially triggering addictive behaviours, and misaligned with recovery values. Some suggested non-monetary rewards (e.g., family experiences) if CM were implemented. |
| Fitzgerald (2015) ^43^ | Key areas identified included by survey:  Limited awareness of alcohol guidelines: Nearly half of respondents were unaware of recommended alcohol consumption limits for men and women, highlighting a significant knowledge gap.  Moderate support for pharmacist involvement: While 56.4% agreed pharmacists could advise on safer drinking, this was lower than support for their role in managing drug misuse (74.4%) or smoking (68.3%).  Demographic influences on perceptions: Men, university-educated individuals, and those from less deprived areas were more likely to endorse pharmacists providing alcohol advice.  High support for specific advisory roles: Over 70% backed pharmacists offering practical guidance (e.g., unit measurement, health impacts, referrals), though fewer (70%) supported discussions about personal drinking levels.  Privacy and trust concerns: While 68.7% trusted pharmacists to maintain confidentiality, 64.3% worried about lack of privacy in pharmacies, and 77.5% preferred discussing alcohol with their doctors. |
| Holland-Hart (2021) ^53^ | The main themes reported included the acceptability, feasibility of the service, and campaign promotion activities. Service users found the pharmacy-based service to be an acceptable initiative, appreciating the fast-track nature that enabled quicker referrals and diagnoses compared to traditional GP routes. They valued the good rapport with pharmacists, which made discussing symptoms comfortable. However, concerns about the feasibility of the service emerged due to confusion at hospitals regarding the new referral process, uncertainty about result timelines, and communication gaps between healthcare professionals. To improve campaign promotion, users suggested making promotional efforts more visible and widespread, using targeted multi-channel campaigns, and ensuring messages were simple and clear while emphasising early diagnosis benefits without focusing excessively on lung cancer, which might discourage some individuals. Overall, participants appreciated the service's accessibility and potential to reduce GP workload but highlighted the need for clearer communication and broader awareness efforts to enhance its effectiveness. |
| Krska (2014) ^19^ | Main themes and survey findings identified included  Patients comfort with pharmacist-led discussions: 80% felt comfortable discussing alcohol with pharmacists.  Strong preference for privacy & confidentiality: 96% emphasised privacy as critical, with concerns about conversations being overheard in open pharmacy areas. Many preferred private consultation rooms over open counters.  Support for proactive but non-intrusive services: While 66% accepted pharmacists initiating alcohol discussions, affluent areas were more resistant. Users preferred non-judgmental, request-based interactions.  Positive reception of screening & referrals: Screening (e.g., AUDIT) was well-received, especially when conducted discreetly. Direct referrals to specialists were preferred over signposting to avoid drop-offs.  Service accessibility & promotion: Users valued walk-in services because of their convenience but highlighted the need for clear signage and tailored promotional materials (e.g., posters in pharmacies/GPs).  Staff competence & approachability: Friendly, trained staff and private consultation spaces enhanced trust. Some questioned pharmacists’ expertise in alcohol advice. |
| Mackridge (2015) ^21^ | Main themes identified included:  Positive reception and rapport: Many participants appreciated the service, highlighting their existing relationship with pharmacy staff as a key factor in their comfort and willingness to engage.  Perceived appropriateness for individuals at risk: While acknowledging the service’s value, some users felt it was more suited for those at higher risk of drinking habits, and so distanced themselves from this group.  Privacy and comfort: Satisfaction with privacy appeared to be high.  Impact on awareness and behaviour: The service prompted some users to reconsider their alcohol consumption, discuss it with others, and even reduce intake, with a few reporting significant lifestyle changes.  Preference for GP Settings: A minority believed alcohol-related discussions were better suited to GP surgeries rather than pharmacies. |
| Madden (2020) ^30^ | The key themes identified included:  Mixed experiences with alcohol service, mixed perceptions of pharmacists' roles, appreciation for medicines reviews, acceptability of alcohol discussions under certain conditions, and self-perception of risk versus actual risk.  Many patients had been asked about alcohol by various health professionals, often in terms of units, but some struggled to relate unit measures to their own drinking. While some viewed pharmacists as only medication dispensers, others recognised their expertise, especially after positive interactions. Patients generally valued medicines reviews for reassurance and advice, though they did not recall much what was delivered in the session. Most were open to alcohol discussions if conducted routinely, confidentially, and non-judgmentally, but some doubted pharmacists had the time or skills for such conversations. A key barrier was the perception that alcohol discussions were irrelevant to them, as many with risky drinking levels (as assessed via AUDIT-C scores 5–10) saw themselves as "moderate" drinkers without a "problem," distancing themselves from the need for intervention. Trust, ongoing relationships, and relevancy were all important factors in the patients’ willingness to engage. |
| Madden (2021) ^31^ | The key themes identified included:  Reframing alcohol as a drug, legitimacy of pharmacist-led discussions, preferred communication styles, and barriers to engagement.  Participants expressed appreciation of viewing alcohol as a drug that interacts with medications, as this provided insights into their health risks, though some resisted the term "drug" due to associated negative connotations. Most agreed pharmacists were well-placed to discuss alcohol if the conversation was medication-focused, but some doubted public awareness of pharmacists' expertise. A non-judgmental, coaching-style approach was preferred, with clear, personalised explanations, as participants valued autonomy in their decision-making. Concerns included feeling targeted, repetitive advice from multiple health professionals, and doubts about pharmacists' communication skills. Participants also highlighted gaps in understanding the purpose of medication reviews and wanted better preparation for such discussions. Overall, they sought relevant, sensitive conversations that balanced facts with individual choice. |
| Poole (2019) ^32^ | The main themes identified included:  Experiential realisation and social processes of change. The experiential realisation theme highlights how physical assessments (e.g., step tests, grip strength) produced teachable moments by challenging men’s self-perceptions of fitness, often leading to unexpected discomfort and a recognition of the need for lifestyle changes. Positive interactions with knowledgeable pharmacy staff reinforced motivation, while technical issues or perceived incompetence undermined confidence in results. The social process of change theme highlights how lifestyle changes were influenced by household dynamics, with dietary and exercise choices often negotiated with partners or family. Self-monitoring tools like pedometers helped men track progress, and social support, whether through family encouragement, friendly competition, or shared activities, played a key role in sustaining changes. Additionally, men’s lifestyle improvements often had a ripple effect, inspiring healthier behaviours in family and friends. |
| Price (2022) ^22^ | The main themes identified included: Quality of service and device satisfaction, health and financial benefits, effectiveness of e-cigarettes for quitting, mental health and stress influences, adjustment challenges, and concerns about vaping.  Participants highly praised the detailed advice and quality of the provided e-cigarettes, which many found otherwise unaffordable. They reported significant health improvements (e.g., better breathing) and financial savings from reduced tobacco use. While e-cigarettes were generally seen as effective, particularly due to their hand to mouth action resembling smoking, some reported a weaker "hit" compared to tobacco. Mental health struggles, especially stress, were key barriers to quitting, with some relapsing during difficult periods like the COVID-19 pandemic. Initial adjustment involved trial and error with different flavours and overcoming temporary side  effects like coughing. Despite the success stories, some participants expressed concerns about long-term vaping risks, perceiving it as an unknown compared to established tobacco harms. Overall, the intervention was well-received, with many praising it for helping reduce or quit smoking. |
| Quirk (2016) ^33^ | The main themes identified included: Motivations for participation, impact of screening and feedback, value of brief interventions and materials, and perceived behavioural changes.  Participants joined the trial for self-assessment ("finding out where I stand"), altruism, or due to trust in their pharmacist's non-judgmental approach. The AUDIT screening process had mixed effects, and some were surprised to learn they drank more than realised, while others felt reassured their consumption was less severe than expected. Brief interventions were appreciated for their empathetic, conversational tone, though some felt the content was mismatched to their drinking level. Printed materials (e.g., unit calculators) were highly valued for practical guidance. About half of participants reported no change in drinking habits, often because they didn’t perceive it to be a problem, while others reported increased awareness or reduced consumption, attributing this to pharmacist discussions or informational leaflets. The pharmacist's role as a trusted, approachable figure was crucial for engagement and positive outcomes. |
| Saramunee (2015) ^24^ | The key themes identified included:  Low current usage but conditional willingness, trust and familiarity barriers, perceptions of pharmacist competency, and service awareness gaps.  While few respondents had previously used pharmacy-based health services (with blood pressure checks being most common), 40% expressed willingness to use health checks, though advisory services (e.g., smoking/drinking/weight advice) saw lower interest. Willingness was higher among women, frequent pharmacy users, and those with fair/good health, but discouraged by distrust due to staff turnover, concerns about confidentiality, and doubts about pharmacists’ advisory skills. Focus group participants highlighted that established GP relationships, self-perceived invincibility (among middle-aged groups), and socioeconomic factors (e.g., healthier lifestyles in affluent areas) further reduced demand. Poor awareness of available services and unclear integration with broader healthcare pathways were also reported as barriers, alongside set views of pharmacies as solely medication dispensers. These findings show the need for improved trust-building, clearer role definition, and targeted outreach to shift public perceptions and increase engagement with pharmacy public health services. |
| Saramunee (2016) ^44^ | The key themes identified included: Preference for convenience and familiarity, trust in confidentiality, range of promotional influences, and mixed views on service promotion.  Survey respondents strongly preferred pharmacies near their home (84.7%) or GP (67.8%), with extended hours (e.g., Saturdays at 63.6%), and they valued continuity and preferred the same pharmacy (56.2%) where staff know them well (34.4%). Participants reported a strong trust in pharmacists' and their confidentiality (89.6%), especially among older, retired, and less-educated groups. Promotional preferences leaned heavily on personal recommendations from healthcare professionals (89.4%) or family/friends (86.5%), with posters in clinics/pharmacies and healthcare websites also known to have an impact. Younger, University educated, and frequent pharmacy users were more receptive to diverse promotional methods, while older adults and infrequent users were less so. Opinions on promotion were divided with some advocating for increased awareness (e.g., via word-of-mouth, social media), while others criticised it as unprofessional or unnecessary, emphasising service quality and doctor referrals as more important than advertisement. These insights highlight the importance of location, trust, and personalised promotion in enhancing pharmacy service engagement. |
| Seston (2020) ^25^ | The key themes identified included goal-setting collaboration, supportive pharmacist relationships, improved health outcomes and self-management, and service accessibility and satisfaction.  Patients valued setting personalised goals (e.g., weight loss, smoking cessation, stress reduction) with pharmacist guidance, though some felt goals were imposed. Supportive, knowledgeable pharmacists who provided tailored advice and encouragement were crucial for engagement and progress, because this fostered trusting relationships. Participants reported significant improvements in health metrics (e.g., blood pressure, cholesterol) and self-management skills, with many achieving their goals. The service enhanced perceptions of the pharmacists' role/expertise and was thought to be easier to access than GP appointments. High satisfaction was reported, though some desired longer-term follow-up. |
| Savickas (2020) ^45^ | The key themes identified included high satisfaction and value, appreciation for professional yet approachable care, increased awareness and education, and convenience and accessibility.  Most participants (99%) rated the experience positively and supported annual screening, with many complimenting the pharmacists' friendly, professional demeanour and clear, lay friendly explanations about AF. The service was valued for improving healthcare access through rapid diagnosis and reassurance, while also contributing to preventative medicine research. A criticism mentioned included appointment duration, but overall feedback was overwhelmingly positive, highlighting the effectiveness of a pharmacist-led approach in delivering accessible, and informative care. |
| Sturrock (2017) ^27^ | The main themes identified included high satisfaction and perceived knowledge gain, positive behavioural intentions, and acceptance of pharmacies as advice venues. An overwhelming majority (71.65%) reported significantly improved oral health knowledge after the intervention, with 65.58% reporting that they will definitely plan to change their dental care habits. Most patients (64.20%) strongly agreed that pharmacies are appropriate settings for oral health advice, though a small minority (3.27%) disagreed. The intervention was particularly impactful for older adults (>65 years), who showed the highest rates of delayed dental visits (29.07%). High patient engagement was evident, with 72.59% accepting referrals to local dental practices, demonstrating the intervention's effectiveness in bridging gaps in oral health awareness and access to services. |
