# Supplementary material for: Integrating cardiovascular healthcare screening into a community pharmacy vaccination service: a scoping review to identify opportunities for patient engagement and service expansion
Source: BMJ Open. 2026 Mar 23;16(3):e108381. doi: 10.1136/bmjopen-2025-108381 (PMC13034389; doi:10.1136/bmjopen-2025-108381)
Supplement: online supplemental table 3 [file bmjopen-16-3-s005.docx]

| **Table 3. What are pharmacists’ experiences of delivery such interventions?** | |
| --- | --- |
| **First Author,**  **Year** | **Key themes** |
| Atkin (2020) ^28^ | Key themes included the pressure to meet pharmacy business targets, particularly with Medicines Use Reviews (MURs), which were often perceived as a contractual obligation rather than a patient-centered service. Pharmacists struggled with competing demands on their time and physical space, juggling dispensing responsibilities, walk-in consultations, and other services (e.g., vaccinations, sexual health clinics), which prevented rapport-building with patients and consequently the quality of consultations. The MUR process itself was described as formulaic, with pharmacists prioritising medication adherence over holistic healthy living discussions due to their discomfort in conversations regarding personal behaviours (e.g., smoking, alcohol) coupled with a lack of time and training to hold such discussions. The organisational culture tended to promote efficiency – completing consultations quickly. Additionally, patients often viewed pharmacists as secondary to GPs for health advice and healthy living. |
| Brown (2014) (a) ^14^ | Key themes identified included:  Success factors: Motivated teams, strong pharmacist leadership, and good patient rapport were crucial for HLP success, alongside PCT support through training and resources.  Implementation challenges: Staffing pressures, training needs, and initial resistance to role changes (from retail to healthcare) posed as significant barriers, particularly in smaller pharmacies.  Motivations for engagement: Pharmacists were driven by professional duty, a desire to expand services, and competitive pressure, viewing HLPs as a way to enhance patient care.  Staff development: HLP participation improved staff confidence, communication skills, and job satisfaction, with HLCs (Healthy Living Champions) feeling empowered to influence patient health behaviours.  Business and community impact: HLPs increased footfall, revenue, and patient loyalty through word-of-mouth referrals, whilst fostering collaboration with GPs and creating a local "pharmacy community."  Workload concerns: Despite benefits, some were concerned about unsustainable workloads, emphasising the need for adequate staffing and fair remuneration for expanded services.  Overall, HLPs were viewed positively but required careful resource management to sustain their benefits. |
| Brown (2014) (b) ^15^ | Key themes identified included:  Implementation barriers: Pharmacists reported time constraints, excessive paperwork, and competing priorities as obstacles to delivering alcohol interventions, with some feeling overwhelmed by administrative tasks.  Intervention introduction: Approaches to bring up alcohol discussions varied – some used neutral prompts (e.g., referencing university studies), while others relied on posters or tools like "drink wheels," though chain pharmacies often restricted promotional materials.  Client engagement challenges: Pharmacists struggled with reluctance from clients, particularly older women and younger teens, fearing embarrassment or damaging trust. Many patients sought quick consultations (e.g., for emergency contraception), as opposed to extended discussions.  Perceived impact: Those who engaged with patients reported positive outcomes, such as patients’ increased awareness of drinking levels supported by use of visual tools. However, low participation rates limited opportunities to improve upon their skills.  Role expansion ambivalence: While many pharmacists welcomed expanded health promotion roles, others felt overburdened. Some questioned targeting emergency contraception patients, and suggested alternative groups (e.g., older adults or medication review patients) for alcohol screening. |
| Holland-Hart (2021) ^53^ | The main themes identified included acceptability, feasibility, and campaign promotion. Sub-themes included barriers to implementation, such as concerns over their authority to refer patients for chest X-rays, their confusion around timing and communication with other healthcare professionals, and completion of lengthy complex paperwork. Facilitators included the fast-track nature of the service, easy access without appointments, and the potential to reduce GP workload while improving early detection. Pharmacists also highlighted the importance of clear, in person training, alongside service expansion to include younger people and non-smokers alike. Regarding campaign promotion, pharmacists emphasised the need for more visible and widespread advertising, using simple messages and targeted campaigns in community venues, pharmacy bags, and even through trained delivery drivers. |
| Krska (2014) ^19^ | Main themes identified included:  Implementation barriers: Pharmacists highlighted workload pressures, lack of private spaces, and being uncomfortable in proactively initiating alcohol discussions.  Staffing shortages and busy environments limited service delivery, though involvement of support staff was seen to address the issue.  Training and confidence: Pharmacists emphasised the need for training to improve knowledge, communication skills, and integration with wider services.  Some pharmacists doubted their expertise in alcohol-specific interventions, preferring reactive (patient-initiated) over proactive approaches.  Service design & referral preferences: Stakeholders agreed on using AUDIT-C for pre-screening by support staff, followed by private pharmacist consultations for full AUDIT.  Direct referral to specialist services was preferred over signposting to GPs.  Promotion and accessibility: Posters in pharmacies/GP surgeries were preferred for raising awareness, but targeting specific demographics was debated and views were mixed.  Facilitators included long opening hours, walk-in access, and visibility of services. |
| Lemanska (2019) ^20^ | Main themes identified included:  Feasibility and accessibility: Pharmacists found the intervention practical to deliver, with community pharmacies being convenient locations for participants. Many believed that the web-based system facilitated the transfer of participants well between pharmacies when required.  Training & confidence: Pharmacy teams successfully acquired the necessary skills through training and demonstrated confidence in delivering the assessments and lifestyle advice components of the intervention.  Participant engagement: The model showed good acceptability with only 15% withdrawal rate during the intervention. High follow-up rates (86% at 1-week, 71% at 6-weeks) indicated strong participant engagement.  Assessment practicalities: While most physical tests (grip strength, sit-to-stand) were feasible, the Siconolfi step test presented challenges due to physical space constraints and participant safety concerns.  Outcome measurement: Accelerometery for measuring physical activity proved feasible (96% baseline compliance) although there was 24% attrition at follow-ups. Significant short-term improvements were reported in physical activity and cardiovascular risk factors.  Safety considerations: Pharmacists effectively identified and managed safety risks during physical assessments, with appropriate referrals made when participants exceeded safe heart rate or blood pressure limits. |
| Mackridge (2015) ^21^ | The main themes identified included: Staff discomfort and coping strategies, perceived appropriateness and rapport, organisational and systemic barriers, and potential for service integration.  Staff often felt uncomfortable questioning customers about alcohol use, leading them to employ strategies such as humour, rephrasing questions, in order to ease tension. Despite this, pharmacists viewed community pharmacies (CPs) as suitable settings for alcohol advice due to already established rapport with customers. However, barriers such as funding, workload constraints, restrictive eligibility criteria, and lack of referral pathways resulted in inconsistent delivery of services. Staff also reported challenges in identifying new service users after initial screenings. On a positive note, some pharmacists observed that involvement in IBA services fostered proactive customer engagement and highlighted opportunities to link alcohol advice with other health services like smoking cessation or weight management, though this type of integration was not routine in practice. |
| Price (2022) ^22^ | The main themes identified included: Positive perceptions of effectiveness, age-related success differences, programme facilitators and barriers, and pharmacists' role as community health promoters. Pharmacists viewed the intervention as highly effective, particularly for middle-aged and older smokers, although younger users were more likely to continue dual use. Key facilitators included widespread advertising, the appeal of free e-cigarettes, the relaxed pharmacy setting, and supportive consultations using carbon monoxide monitoring for motivation. However, barriers such as participant disengagement, short intervention timelines, supply issues, and lack of GP referrals were reported. Pharmacists also highlighted their unique position as accessible community health advisors, helping destigmatise e-cigarettes and fostering trust with service users. The intervention was believed to raise the profile of smoking cessation, though challenges like stress (exacerbated by COVID-19) and reliability of the product impacted on long-term success. |
| Sturrock (2017) ^27^ | The main themes identified included positive engagement and impact, enhanced knowledge and awareness, and opportunities for service expansion.  Pharmacy staff reported strong enthusiasm for the intervention, reporting high patient satisfaction and ease of recruitment, with many patients appreciating the advice and returning with positive feedback. The training effectively improved staff knowledge, particularly regarding fluoride content in toothpaste and broader oral health issues beyond just teeth. Staff identified key gaps in patient understanding, such as proper brushing techniques and denture care, which allowed for more targeted counselling. Looking ahead, participants suggested expanding the service to include children and housebound patients, improving signposting to dental practices, and strengthening interprofessional communication with dentists to ensure continuity of care and follow-up. Overall, the intervention was viewed as highly successful with potential for broader implementation. |
