# Supplementary material for: Integrating cardiovascular healthcare screening into a community pharmacy vaccination service: a scoping review to identify opportunities for patient engagement and service expansion
Source: BMJ Open. 2026 Mar 23;16(3):e108381. doi: 10.1136/bmjopen-2025-108381 (PMC13034389; doi:10.1136/bmjopen-2025-108381)
Supplement: Supplementary file 6 [file bmjopen-2025-108381.reviewer_comments.pdf]

PEER REVIEW HISTORY

BMJ Open publishes all reviews undertaken for accepted manuscripts. Reviewers are asked to complete a checklist review form and are provided with free text boxes to elaborate on their assessment. These free text comments are reproduced below.

ARTICLE DETAILS

Title (Provisional)

Integrating cardiovascular healthcare screening into a community pharmacy vaccination service: A scoping review to identify opportunities for patient engagement and service expansion

Authors

Tang, Jason; Mansell, Gemma; Merks, Piotr; Jones, Alison; Badenhorst, Jay; Koziol, Mark; Sandhu, Daljit; Langley, Chris

---

VERSION 1 - REVIEW

**Reviewer**	**1**
**Name**	**Konya , Judit**
**Affiliation**	**University of Exeter**
**Date**	**13-Oct-2025**
**COI**	**I recently completed the following project:**

https://www.spcr.nihr.ac.uk/research/projects/early-cancer-diagnosis-and-community-pharmacies-in-deprived-areas .
The first paper, a systematic review is published and is a similar structure.
https://bjgp.org/content/early/2025/09/12/BJGP.2025.0204
I don't think this is a conflict, but best declared here.

With great interest I read the submitted manuscript, and I wish to praise you for completing the work. It is admittedly challenging to present the findings of the review when the outcomes and methods of the included papers are so diverse and I appreciate how much work must have gone into writing the paper.

I wish to raise a few minor points, and I feel by addressing these, the readability of the paper will further improve.

1. Abstract and identifying relevant studies. Please provide justification for the date range - why November 2013?

2. Page 3 line 42-45. Please clarify this paragraph avoiding starting the paragraph with "This service" and "these" as it is not clear what service it refers to, without having to re-read the previous paragraph.

3. Page 5: please clarify the research question and objectives, they are currently presented mixed.

4. Page 5 Inclusion and Exclusion criteria. Under "non-pharmacist-led" intervention do you mean pharmacist or pharmacy staff? As not all staff in pharmacies will be pharmacists and this is reflected in the included studies.

5. Page 5 Inclusion criteria 3: Please explain what do you mean under "brief interactive sessions".

6. Presentation of the results answering research questions/objectives.

I wonder if a slight restructure would result in clearer presentation of the findings. This suggestion related back to my 3. point raised above.

In the results section, you answer five research questions. Please make sure these align with the research questions/objectives. The sections under each question would be easier to follow if they started with a summary paragraph, such as on page 7 line 13-24. Here it is clear which included studies address what, as the references are all listed. Page 7 lines 20-24 also clearly summarises the following paragraphs between page 7 line 26-page 8 line 39, however it is not clear how page 8 line 41-60 relates to page 7 line 9-24.

A similar summary paragraph listing the relevant included papers' citations would be helpful under each research question.

7. Page 10 line 16-26. Not clear if the Behavioural change is related to the second research question? Simple font formatting could resolve this.

8. Looking at the list of included studies (Table 1) I wonder if it could be clearer how are some of publications (for example Holland-Hart and Sturrock) address the inclusion criteria? Similarly, included papers assessing the NMS service when it is not explicit what medication the service is related to or the patients involved. I am aware that a great deal of discussion is involved within the team regarding inclusion/exclusion, however I feel this should be reflected more clearly either in the paper itself or Table 1.

**Reviewer**	**2**
**Name**	**Scicchitano, Pietro**
**Affiliation**	**ASL Bari, Cardiology**
**Date**	**20-Nov-2025**
**COI**	**None**

- authors should discuss the role of care manager in this setting. They can consider the paper from Ciccone MM et al. Vasc Health Risk Manag. 2010 May 6;6:297-305.

**Reviewer**	**3**

Name Isabella , Tan,

Affiliation University of New South Wales

Date 24-Dec-2025

COI None

The authors present a detailed scoping review on cardiovascular screening / behavioral interventions implemented in community pharmacies. I have the following comments:

- The introduction/methods could clarify how cardiovascular screening/behavioral change interventions integrated into other pharmacy-led services could be adopted into vaccination services. The scoping review itself was not limited to vaccination services, thus the title could be revised to better reflect the scoping review aim and results (e.g. "Cardiovascular-related interventions in community pharmacist-led services...")?

- The term "cardiovascular outcomes" often refer to conditions such as heart failure, major adverse cardiovascular events, etc. For this scoping review, it may be more appropriate to use the term to "cardiovascular risk factors" (e.g. on page 5, line 53)?

- For Supplementary Table 1, it may help to summarize results and conclusions into bullet points, and have number of participants and other characteristics in separate columns. It may also be helpful, if possible, to categorize the type of service within which the screening/interventions were integrated (e.g. vaccination service, blood pressure screening service). The authors can consider having a summary table in the main manuscript as reference for readers.

- The abstract stated five studies described successful integration of lifestyle interventions within vaccination services, but I was unable to locate these studies in Supplementary Table 1 or the manuscript text. Did the authors mean successful integration within pharmacies, as per line 20 on page 7 of the manuscript?

- The question "What cardiovascular interventions can be delivered as part of pharmacist-led vaccination services?" may be better rephrased as "What cardiovascular interventions have been delivered..." As suggested above, it would be informative to then categorize the types of services within which those interventions were delivered. There can then be a subsection on "Feasibility and acceptability of integration of cardiovascular-related interventions in pharmacist-led services".

- One of the major challenges with screening interventions is referral to care and follow-up for ongoing treatment. Whilst noted for individual studies in Supplementary Table 1, it would be informative to include this as part of the results (e.g. mechanisms for referral, referral rates, follow-up rates etc) and discussion.

- Page 9, lines 17-18, it may be helpful to number the key themes identified for clarity.

- Page 10, line 16, Heading for "Behavioual change" should match the previous themes (i.e. italic and not bolded)

- Please rename supplementary tables as Supplementary Table 1, Supplementary Table 2, Supplementary Table 3 (or as per naming convention suggested in the journal's Author Guidelines)

VERSION 1 - AUTHOR RESPONSE

Reviewer 1 comments

1. Abstract and identifying relevant studies. Please provide justification for the date range - why November 2013?

*We selected this date range because, from around 2013 onwards, there was a significant shift in the role of community pharmacies in the UK, in that, pharmacies increasingly expanded beyond dispensing medicines to also include delivery of public health and preventative services, which included health screening and lifestyle interventions. Limiting our search in this way, not only made our review more manageable, but ensured that our findings are likely relevant to the community pharmacy context today. We have now clarified this in the Methods section on p.5.

2. Page 3 line 42-45. Please clarify this paragraph avoiding starting the paragraph with "This service" and "these" as it is not clear what service it refers to, without having to re-read the previous paragraph.

*Sorry – we have now amended for clarity, specifying the service that is being referred to on p.3.

3. Page 5: please clarify the research question and objectives, they are currently presented mixed.

*We have now clarified the research question from the objectives by stating clearly the overarching research question, and the key questions used to address it (p.5). It now reads:

"The primary objective of this scoping review was: 'to explore what cardiovascular interventions can be delivered as part of community pharmacist-led vaccination services?' To address this overarching research question, the review explored the following key questions…."

4. Page 5 Inclusion and Exclusion criteria. Under "non-pharmacist-led" intervention do you mean pharmacist or pharmacy staff? As not all staff in pharmacies will be pharmacists and this is reflected in the included studies.

*Thank you. We agree and have now revised to "studies focused exclusively on non-pharmacy-based interventions" to avoid misinterpretation (p.6).

5. Page 5 Inclusion criteria 3: Please explain what do you mean under "brief interactive sessions".

*We have now clarified what we mean by ‘brief interactive sessions’ in parenthesis within the inclusion, defined as some form of engagement between the pharmacy staff and service users as opposed to, for example, ‘passive’ advice on a poster within the pharmacy waiting area (p.5).

6. Presentation of the results answering research questions/objectives.
I wonder if a slight restructure would result in clearer presentation of the findings. This suggestion related back to my 3. point raised above. In the results section, you answer five research questions. Please make sure these align with the research questions/objectives.

*We have now amended the research questions (as above to address query 3) so that they are presented more clearly and each addressed in turn, in order, within our findings.

The sections under each question would be easier to follow if they started with a summary paragraph, such as on page 7 line 13-24. Here it is clear which included studies address what, as the references are all listed. Page 7 lines 20-24 also clearly summarises the following paragraphs between page 7 line 26-page 8 line 39, however it is not clear how page 8 line 41-60 relates to page 7 line 9-24.
A similar summary paragraph listing the relevant included papers' citations would be helpful under each research question.

*Thank you for this helpful suggestion. We have amended the opening line of the paragraph on page 8 to improve the flow. It now reads: “Not all findings, however, from this group of studies, addressing this research question, were positive”. Additionally, from the outset, we have also included for each RQ the relevant included papers’ citations before discussing their findings (pp.9, 11, 13).

7. Page 10 line 16-26. Not clear if the Behavioural change is related to the second research question? Simple font formatting could resolve this.

*We have now amended so that it reads more clearly as identified themes (pp.9-10).

8. Looking at the list of included studies (Table 1) I wonder if it could be clearer how are some of publications (for example Holland-Hart and Sturrock) address the inclusion criteria? Similarly, included papers assessing the NMS service when it is not explicit what medication the service is related to or the patients involved. I am aware that a great deal of discussion is involved within the team regarding inclusion/exclusion, however I feel this should be reflected more clearly either in the paper itself or Table 1.

*Thank you for this comment. All studies included in the review met our eligibility criteria and were assessed by two independent reviewers. We recognise that some study descriptions were broad or lacked detailed reporting. However, eligibility was confirmed at full-text stage through careful review of each paper and any included supplementary

materials to ensure they met each inclusion criteria. If still desired, we would be happy to revisit each included study and explicitly specify which phrases or descriptions were used to confirm eligibility.

Reviewer 2 comments

1. Authors should discuss the role of care manager in this setting. They can consider the paper from Ciccone MM et al. Vasc Health Risk Manag. 2010 May 6;6:297-305.

*Thank you for the suggestion. We acknowledge the important role that care managers has in cardiovascular risk management. However, focusing on this specific role and their contribution was beyond the scope of this review as we focused on pharmacy-based interventions delivered within community pharmacy settings in the UK.

Reviewer 3 comments

1. The introduction/methods could clarify how cardiovascular screening/behavioral change interventions integrated into other pharmacy-led services could be adopted into vaccination services. The scoping review itself was not limited to vaccination services, thus the title could be revised to better reflect the scoping review aim and results (e.g. "Cardiovascular-related interventions in community pharmacist-led services...")?

*Rather than changing the title, we have clarified in the introduction that the scoping review was not restricted to vaccination-based studies, but instead examined more broadly cardiovascular-related interventions delivered across community pharmacy settings to identify transferable components and delivery features that could support integration into vaccination services (p.4).

2. The term "cardiovascular outcomes" often refer to conditions such as heart failure, major adverse cardiovascular events, etc. For this scoping review, it may be more appropriate to use the term to "cardiovascular risk factors" (e.g. on page 5, line 53)?

*Thank you for highlighting this. We have now replaced this term to “cardiovascular risk factors” throughout (pp. 5,6).

3. For Supplementary Table 1, it may help to summarize results and conclusions into bullet points, and have number of participants and other characteristics in separate columns. It may also be helpful, if possible, to categorize the type of service within which the screening/interventions were integrated (e.g. vaccination service, blood pressure screening service). The authors can consider having a summary table in the main manuscript as reference for readers.

*Thank you for this suggestion. Table 1 was structured using the current headings and columns to provide a clear and comprehensive overview of each study within its context. Given the substantial heterogeneity in intervention content and delivery, we decided not to structure the information using bullet points or categorical groupings, as this could result in

the loss of important contextual detail. We therefore prefer to retain the current table format as this would allow readers to examine individual studies, with key differences and patterns synthesised narratively in the main text. We believe that this approach improves clarity as it retains the contextual differences between interventions or services and avoids giving the impression that some of the studies and interventions are directly comparable.

4. The abstract stated five studies described successful integration of lifestyle interventions within vaccination services, but I was unable to locate these studies in Supplementary Table 1 or the manuscript text. Did the authors mean successful integration within pharmacies, as per line 20 on page 7 of the manuscript?

*Thank you for picking this up. Yes, that is correct that these five studies identified describe integration of lifestyle interventions within pharmacy-based settings rather than with vaccination services. We have now clarified this in the abstract (p.2).

5. The question "What cardiovascular interventions can be delivered as part of pharmacist-led vaccination services?" may be better rephrased as "What cardiovascular interventions have been delivered..." As suggested above, it would be informative to then categorize the types of services within which those interventions were delivered. There can then be a subsection on "Feasibility and acceptability of integration of cardiovascular-related interventions in pharmacist-led services".

*Thank you. Although the included studies describe cardiovascular-related interventions that have been implemented within pharmacist-led services, the review question was intentionally framed using “can be delivered” to reflect the broader aim of identifying interventions with potential for integration into vaccination services, rather than limiting the review to interventions already integrated with vaccination delivery. As outlined previously, these interventions were delivered across a range of pharmacist-led service contexts and were synthesised narratively rather than categorised extensively by service type so that we could retain contextual detail and avoid implying homogeneity. As a result, feasibility and acceptability are addressed across the results and discussion sections, rather than presented separately.

6. One of the major challenges with screening interventions is referral to care and follow-up for ongoing treatment. Whilst noted for individual studies in Supplementary Table 1, it would be informative to include this as part of the results (e.g. mechanisms for referral, referral rates, follow-up rates etc) and discussion.

*Thank you for pointing out this important issue of onward referral and follow up for ongoing treatment. In response, we have added a short section within the results to summarise referral mechanisms, their uptake, and follow up reported across relevant studies (p.9). We have also included a short paragraph in the discussion to highlight the importance of referral and to encourage more consistent reporting of referral and follow up outcomes, where possible, in future studies (p.15).

7. Page 9, lines 17-18, it may be helpful to number the key themes identified for clarity.

*Thank you for this helpful suggestion. We have now numbered the key themes (pp. 9-10).

8. Page 10, line 16, Heading for "Behavioual change" should match the previous themes (i.e. italic and not bolded)

*Sorry for this error – now changed (p.10)

9. Please rename supplementary tables as Supplementary Table 1, Supplementary Table 2, Supplementary Table 3 (or as per naming convention suggested in the journal's Author Guidelines)

*Sorry – tables now renamed (pp. 9,11)

VERSION 2 - REVIEW

**Reviewer**	**3**
**Name**	**Isabella , Tan,**
**Affiliation**	**University of New South Wales**
**Date**	**26-Feb-2026**
**COI**	

Thank you to the authors for the considered responses and for this insightful scoping review. I have no further comments, other than suggesting removal of the numbering of the key themes on pp9-10, as other themes in the subsequent sections were not numbered. My original suggestion was to number them such that they could be cross-referenced to Supplementary Tables 2 and 3. However, I understand this may be too cumbersome.
